# Associations Between Exercise Training, Physical Activity, Sedentary Behaviour and Mortality: An Umbrella Review of Meta‐Analyses

**DOI:** 10.1002/jcsm.13772

**Published:** 2025-03-05

**Authors:** Masoud Rahmati, Hyeri Lee, Hayeon Lee, Jaeyu Park, Djandan Tadum Arthur Vithran, Yusheng Li, Abdolreza Kazemi, Laurent Boyer, Guillaume Fond, Lee Smith, Nicola Veronese, Pinar Soysal, Elena Dragioti, Samuele Cortese, Jiseung Kang, Dong Keon Yon, Marco Solmi

**Affiliations:** ^1^ CEReSS‐Health Service Research and Quality of Life Center, Assistance Publique‐Hopitaux de Marseille Aix‐Marseille University Marseille France; ^2^ CRSMP, Center for Mental Health and Psychiatry Research – PACA Marseille France; ^3^ Department of Physical Education and Sport Sciences, Faculty of Literature and Human Sciences Lorestan University Khoramabad Iran; ^4^ Department of Physical Education and Sport Sciences, Faculty of Literature and Humanities Vali‐E‐Asr University of Rafsanjan Rafsanjan Iran; ^5^ Center for Digital Health, Medical Science Research Institute, Kyung Hee University Medical Center Kyung Hee University College of Medicine Seoul Republic of Korea; ^6^ Department of Orthopedics, Xiangya Hospital Central South University Changsha China; ^7^ Centre for Health, Performance and Wellbeing Anglia Ruskin University Cambridge UK; ^8^ Saint Camillus International University of Health Sciences Rome Italy; ^9^ Department of Geriatric Medicine, Faculty of Medicine Bezmialem Vakif University Istanbul Turkey; ^10^ Pain and Rehabilitation Centre, and Department of Medical and Health Sciences Linkoping University Linkoping Sweden; ^11^ Research Laboratory Psychology of Patients, Families & Health Professionals, Department of Nursing, School of Health Sciences University of Ioannina Ioannina Greece; ^12^ Developmental EPI (Evidence synthesis, Prediction, Implementation) Lab, Centre for Innovation in Mental Health, School of Psychology, Faculty of Environmental and Life Sciences University of Southampton Southampton UK; ^13^ Clinical and Experimental Sciences (CNS and Psychiatry), Faculty of Medicine University of Southampton Southampton UK; ^14^ Hampshire and Isle of Wight Healthcare NHS Foundation Trust Southampton UK; ^15^ Hassenfeld Children's Hospital at NYU Langone New York University Child Study Center New York New York USA; ^16^ DiMePRe‐J‐Department of Precision and Regenerative Medicine‐Jonic Area University of Bari “Aldo Moro” Bari Italy; ^17^ Department of Anesthesia, Critical Care and Pain Medicine Massachusetts General Hospital Boston Massachusetts USA; ^18^ Division of Sleep Medicine Harvard Medical School Boston Massachusetts USA; ^19^ School of Health and Environmental Science, College of Health Science Korea University Seoul South Korea; ^20^ Department of Pediatrics Kyung Hee University College of Medicine Seoul Republic of Korea; ^21^ On Track: The Champlain First Episode Psychosis Program, Department of Mental Health The Ottawa Hospital Ottawa Ontario Canada; ^22^ Clinical Epidemiology Program, Ottawa Hospital Research Institute (OHRI) University of Ottawa Ottawa Ontario Canada; ^23^ Department of Child and Adolescent Psychiatry Charité Universitätsmedizin Berlin Germany; ^24^ Department of Psychiatry University of Ottawa Ottawa Ontario Canada

**Keywords:** All‐cause mortality, Cancer mortality, Cardiovascular disease mortality, Exercise training, Physical activity, Sedentary behavior

## Abstract

**Background:**

Numerous studies support the association of exercise training, physical activity (PA) and sedentary behaviour (SB) with both mortality and morbidity outcomes. The results across studies have been inconsistent, and no umbrella reviews have yet been conducted on this topic.

**Methods:**

We conducted an umbrella review of meta‐analyses of observational studies by screening articles in PubMed/MEDLINE, EMBASE and Web of Science databases from inception to 30 April 2024. Quality appraisal of each included meta‐analysis was done using the AMSTAR 2 tool, with evidence certainty evaluated based on statistical significance, study size, heterogeneity, small‐study effects, prediction intervals (PI) and potential biases.

**Results:**

Frothy‐eight meta‐analyses were included (AMSTAR 2 ratings: high 25, moderate 10, low 2 and critically low 11). No evidence was highly suggestive or convincing. Suggestive evidence linked any PA and SB to lower and higher risks of all‐cause, cardiovascular and cancer mortality. Suggestive evidence indicated a significant association between self‐reported and device‐measured total PA (equivalent odds ratio [eOR] 0.78 [0.70–0.86] and eHR = 0.50 [0.38–0.65], respectively), self‐reported leisure time PA (eHR = 0.73 [0.66–0.80]), device‐measured daily steps (eHR = 0.44 [0.35–0.56]) and aerobic plus resistance training (eHR = 0.60 [0.56–0.64]) with lower all‐cause mortality. Weak evidence supported links between self‐reported and device‐measured SB and higher mortality (eHR = 1.3 [1.22–1.38] and eHR = 2.16 [1.09–4.28], respectively). Suggestive evidence was noted for the association between self‐reported leisure time PA (eHR = 0.74 [0.69–0.80]) and resistance training (eHR = 0.82 [0.81–0.84]) with cardiovascular mortality. Suggestive evidence was also found for the association between self‐reported leisure time PA (eHR = 0.87 [0.83–0.91]) with cancer mortality. Associations between self‐reported running time and mortality from all causes, cardiovascular diseases (CVD) and cancer did not reach statistical significance nor did the association between low skeletal muscle mass and all‐cause mortality. Meta‐regression analyses showed that physical activity reduces mortality risk, with age reducing the protective effects against all‐cause, CVD and cancer mortality. We also found that combined exercise training (aerobic plus resistance) most effectively reduces all‐cause and CVD mortality.

**Conclusions:**

Converging evidence supports that physical activity and sedentary behaviour are associated with lower and higher rates of all‐cause, cardiovascular and cancer mortality. More high‐quality prospective studies are needed for a better understanding of the associations between running time and also TV‐viewing time and health‐related outcomes.

## 1 Introduction

Cardiovascular diseases (CVD) and cancers are the leading global causes of mortality and morbidity, contributing to 32% and 17% of all reported deaths worldwide in 2019, respectively [1]. Lack of physical activity (PA) and prolonged sedentary behaviour (SB) are two key modifiable risk factors that have been associated with higher hazards of mortality from all causes, from CVD and from cancers. Considering the existence of strong evidence documenting that physical inactivity (defined as not meeting PA recommendations as 150 min of moderate to vigorous PA per week) has been attributed to more than five million premature deaths a year worldwide [2], few people meet the recommended levels of PA [3]. Consequently, many countries and scientific authorities have released PA guidelines or have emphasized the potential benefits of reducing prolonged SB [4, 5]. The updated 2020 WHO guidelines build upon the previous 2010 recommendations, reinforcing the message that engaging in any amount of PA is beneficial, with increased levels of activity linked to better health outcomes. In addition to reemphasizing the need to minimize SB, these guidelines stress the importance of regularly incorporating both aerobic and resistance training. These guidelines serve as a foundation for shaping national health policies in alignment with the WHO Global Action Plan on Physical Activity 2018–2030 and support the enhancement of systems for tracking progress towards both national and global health targets [4].

Most epidemiological evidence on PA and health is based on studies that assessed the mortality and morbidity rates according to different forms of movement behaviours represented as total PA (TPA) or further classified into distinct categories of intensity‐based PA, that is, vigorous‐intensity PA (VPA), moderate‐to‐vigorous‐intensity PA (MVPA), light‐intensity PA (LPA), daily step counts and prolonged SB. Previous meta‐analyses on the association between these forms of PA and health outcomes concluded that a higher level of PA was significantly associated with lower mortality from all causes, CVD and cancers. Hermelink et al. [6] found positive associations between SB and cancer incidence and mortality, whereas de Rezende et al. [7] reported that PA reduces cancer risks and overall cancer mortality. However, these reviews did not evaluate the combined effects of PA and SB on all‐cause mortality or CVD, nor did they differentiate between self‐reported and device‐measured PA and SB. Additionally, Kraus et al. [8] and Fukushima et al. [9] focused primarily on structured forms of PA, such as TPA and LTPA, without considering other forms of PA and SB and did not address the credibility of evidence in their dose–response analyses.

Overall, these findings indicate that PA‐related umbrella reviews are mostly restricted to cancer or mortality outcomes. There is currently no umbrella review that encompasses various forms of PA, including TPA, LTPA, walking time, running time, daily steps, resistance training, aerobic training and combined aerobic plus resistance training, along with SB and cardiorespiratory fitness (CRF), with a focus on mortality from all causes, CVD and cancer. This study, however, goes beyond examining general PA and SB by including the effects of different types of exercise interventions, such as aerobic training, resistance training and their combination, on mortality outcomes. By evaluating the impacts of both general PA and specific exercise modalities, we aim to provide a more comprehensive understanding of how various forms of PA contribute to health outcomes, which has not been systematically reviewed in previous umbrella reviews. Therefore, the purpose of this study was to synthesize published meta‐analyses on the association of all domains of PA and SB with mortality from all causes, CVD and cancer.

## 2 Methods

### 2.1 Searches and Inclusion Criteria

We conducted an umbrella review of meta‐analyses of observational studies that reported on outcomes associated with different levels and types of PA and SB and all‐cause mortality, CVD mortality or any cancer mortality.

We followed an a priori protocol (PROSPERO; ref. No. CRD42024519058) and adhered to PRIOR and PRISMA 2020 guidelines (adapting PRISMA to the abstract of an umbrella review; Tables S1 and S2) [10, 11, 12, 13, 14, 15]. Two authors independently screened the references retrieved systematically by searching PubMed/MEDLINE, EMBASE and Web of Science databases up to 30 April 2024, without language restrictions, and extracted data into a spreadsheet. We also manually searched the Cochrane Library. To identify additional studies, we also conducted extensive manual searches of the reference lists of the retrieved review articles. The search key is available in the supplementary methods. We excluded systematic reviews without a meta‐analysis, pooled analyses of studies identified without a systematic search and individual studies. Two researchers (M.R. and D.K.Y.) independently searched the existing literature and extracted data for each eligible article. For each study, the title, abstract and keywords were reviewed for inclusion, and any ambiguity was resolved through discussion by a third researcher (H.L.) [16, 17, 18, 19, 20].

The outcome of interest was the association of different domains of PA and SB on all‐cause mortality, CVD mortality or any cancer mortality in previously available meta‐analyses that only included observational studies. TPA, LTPA, running time, step counts and resistance training, as well as SB and TV‐viewing time, were considered as different domains of PA. We also included studies that reported the association between CRF and health outcomes. Additionally, we included pooled analysis studies that conducted a systematic search and meta‐analysis.

### 2.2 Data Extraction and Quality Assessment

Extracted information from meta‐analyses and individual studies included in meta‐analyses were first author, year of publication, design of included studies, number of included studies in the meta‐analysis, the exposure and comparison definitions, the outcomes and their effect size and dispersion measure (when adjusted and unadjusted effect sizes were reported, we selected the most adjusted ones). Data extraction procedures were done by two independent researchers (M.R. and D.K.Y.). The methodological quality of each included meta‐analysis was assessed independently by two researchers (M.R. and D.K.Y.) using A Measurement Tool to Assess Systematic Reviews Version 2 (AMSTAR 2) [21]. In cases of disagreement, a consensus was reached through discussion with a third investigator (H.L.).

### 2.3 Data Analysis

For each association from observational studies (i.e., between different domains of PA and SB and outcomes), we extracted the effect sizes of individual studies reported in each meta‐analysis, recalculating the pooled effect sizes and 95% confidence intervals, using random effects models. To reduce inappropriate Type 1 errors, we reanalysed each eligible association under the random effects model using DerSimonian and Laird method (if included studies were equal or more than 10) [22] and Hartung‐Knapp‐Sidik‐Jonkman (if less than 10) [23]. We also tested the heterogeneity with the *I* ^2^ statistics and *p* curve was assessed to detect potential p‐hacking with values > 50% [24]. Moreover, to estimate the possible range in which the effect sizes of future studies were anticipated to fall, 95% prediction intervals for the summary random effect sizes were computed [25]. Prediction intervals were calculated using both the estimated between‐study heterogeneity variance given from *tau* ^2^ as well as the standard error of the pooled effect [26]. We then analysed small study effect bias, investigating whether smaller studies produced larger effect sizes compared with larger studies [27]. We considered the presence of small study effect when two conditions were met: First, the Egger regression asymmetry test indicated publication bias (*p* value ≤ 0.10), and second, the random effects summary effect size surpassed the effect size of the largest study contributing to that particular association [27, 28]. Sensitivity analyses were conducted to include influence analysis and Baujat plots. Studies rated as *low* or *critically low* in the AMSTAR 2 quality assessment were excluded from these analyses. Influence analyses were subsequently carried out to examine the effect of individual studies on the pooled estimate by systematically excluding one study at a time and re‐estimating the overall effect size [29]. A Baujat plot was generated to present each study's contribution to heterogeneity and publication bias, facilitating the identification of potential outliers [29, 30, 31]. Furthermore, to assess the robustness of the findings, meta‐analyses were repeated on the reduced dataset using the Hartung–Knapp–Sidik–Jonkman random effects model, enabling a direct comparison of results. Lastly, we assessed significance bias using an updated approach to identify the publication selection of statistically significant findings through observable excess statistical significance [32, 33]. We performed calculations for the test of excess statistical significance and the proportion of statistical significance, ensuring proper control for Type I errors and achieving high statistical power and to evaluate whether the expected number of studies (*E*) differs from the actual observed number of studies (*O*) with statistically significant results (*p* < 0.05) included in each meta‐analysis [33]. To evaluate the potential impact of age and different measurement method (self‐reported and device‐measured) on the relationship between physical activity and mortality, a random‐effects meta‐regression analyses were conducted. The dependent variable was the Fisher *z*‐transformed correlation coefficient, and age or measurement method was used as the moderator (independent variable) in the analysis, employing the restricted maximum likelihood (REML) approach. We also performed four sets of subgroup analyses by different follow‐up period (< 10 years vs. ≥ 10 years), different measurement method (self‐reported vs. device‐measured), different exercise training modality (resistance training versus aerobic training or resistance training plus aerobic training) and dose–response relationship between physical activity and mortality (high vs. inactive, moderate vs. inactive, and low vs. inactive). All analyses were conducted in R (Version 4.2.2; R Foundation, Vienna, Austria), Review Manager (Version 5.4; The Nordic Cochrane Centre, Copenhagen, Denmark) or Comprehensive Meta‐analysis (Version 3.3; Biostat Inc., Englewood, NK), and a two‐sided *p* value < 0.05 was considered significant [19, 34].

### 2.4 Assessment of the Credibility of Evidence

Associations between different levels of PA and SB and the risk of all‐cause mortality, CVD mortality or any cancer mortality were classified into five levels of evidence strength in accordance with grading schemes applied in previously published umbrella reviews [6, 26, 28, 35] as convincing (*p* < 10^−6^, > 30 000 cases, *p* < 0.05 of the largest study in meta‐analysis, *I* ^2^ < 50%, no small study effect; prediction interval excludes the null value; no excess significance bias), highly suggestive (*p* < 10^−6^, > 1000 cases, *p* < 0.05 of the largest study in meta‐analysis), suggestive (*p* < 10^−3^, > 1000 cases), weak (*p* < 0.05) or no association (*p* > 0.05). Evidence of strong statistical significance using random‐effects meta‐analyses at *p* value < 50%, absence of small study effects (Egger *p* value > 0.10) and 95% PI were excluded from the null [28].

## 3 Result

**FIGURE 1:**
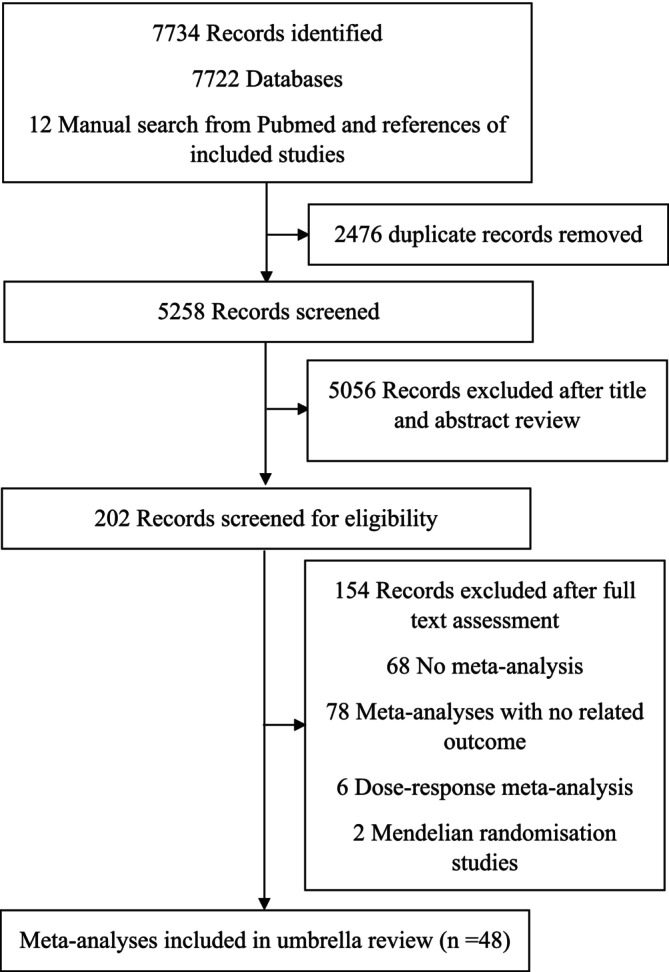
Study selection flow. References of excluded studies after full text assessment available in Table S3.

Starting from 5258 records after duplicate removal, we excluded 5057 studies at title and abstract screening stage and 154 at full‐text level, resulting in 48 publications being included. All excluded studies after full‐text assessment, with reason for exclusion, are listed in Table S3, and the article selection flow is represented in Figure 1. The eligible meta‐analyses of prospective cohorts were published between 2008 and 2023. The quality of included meta‐analyses according to AMSTAR 2 was high in 25 meta‐analyses, moderate in 10, low in 2 and critically low in 11 (Table 1). Overall, 1018 unique meta‐analytical associations were identified reporting on the association between self‐reported TPA, device‐measured TPA, self‐reported LTPA, self‐reported walking time, self‐reported running time, device‐measured daily steps, resistance training, aerobic training, aerobic plus resistance training, device‐measured CRF, CRF per one‐MET increase in CRF, self‐reported SB, device‐measured SB, self‐reported TV‐viewing and low skeletal muscle mass with mortality from all causes, CVD and cancer in healthy population.

**TABLE 1:** General characteristics of included studies.

Study	*k*	Population (age)	Mean years follow‐up	Exposure	Comparison	Outcome	Main results (HR ([95% CI])	Quality
Arem et al.^a^ [36]	6	21–98	14.2	Self‐reported TPA	High vs. inactive population Moderate vs. inactive population Low vs. inactive population High vs. inactive population Moderate vs. inactive population Low vs. inactive population High vs. inactive population Moderate vs. inactive population Low vs. inactive population	All‐cause mortality CVD mortality Cancer mortality	0.72 (0.68–0.76) 0.71 (0.68–0.73) 0.73 (0.71–0.75) 0.58 (0.56–0.61) 0.59 (0.57–0.63) 0.67 (0.65–0.70) 0.74 (0.71–0.77) 0.75 (0.72–0.79) 0.79 (0.75–0.82)	Critically low
Banach et al. [ 37 ]	17	≥ 18	7.1	Device‐measured daily steps	High vs. very low Moderate vs. very low Low vs. very low Per 1000 steps increment High vs. very low Moderate vs. very low Low vs. very low Per 500 steps increment	All‐cause mortality CVD mortality	0.33 (0.25–0.43) 0.45 (0.33–0.60) 0.51 (0.47–0.56) 0.85 (0.81–0.91) 0.33 (0.28–0.44) 0.51 (0.42–0.62) 0.84 (0.73–0.97) 0.93 (0.91–0.95)	Moderate
Biswas et al. [ 38 ]	41	18–90	9.1	Self‐reported SB	High vs. low ST	All‐cause mortality CVD mortality Cancer mortality	1.24 (1.09–1.41) 1.18 (1.09–1.26) 1.17 (1.1–1.24)	High
Blond et al. [39]	48	60	2	Self‐reported running time Device‐measured running time Self‐reported TPA Self‐reported LTPA	Higher levels of PA vs. the recommended level (30 vs. 7.5 MET h/week)	All‐cause mortality CVD mortality Cancer mortality All‐cause mortality CVD mortality Cancer mortality All‐cause mortality CVD mortality Cancer mortality All‐cause mortality CVD mortality Cancer mortality	0.83 (0.80–0.85) 0.81 (0.77–0.85) 0.89 (0.86–0.92) 0.47 (0.34–0.65) 0.80 (0.71–0.90) 0.93 (0.79–1.11) 0.88 (0.85–0.91) 0.76 (0.66–0.88) 0.88 (0.78–0.98) 0.90 (0.77–1.05) 0.82 (0.68–1.00) 0.93 (0.79–1.11)	High
Chastin et al. [3]	8	54	9.4	Device‐measured TPA	Light PA vs. inactive population	All‐cause mortality	0.71 (0.62–0.73)	Moderate
Chastin et al.^a^ [40]	6	54	9.4	Device‐measured TPA	MVPA vs. inactive population Light PA vs. inactive population	All‐cause mortality	0.63 (0.55–0.71) 0.5 (0.42–0.62)	Moderate
Chau et al. [ 41 ]	6	18–74	5.3	Self‐reported/device‐measured SB	High vs. low SB	All‐cause mortality	1.34 (1.28–1.40)	Moderate
Cheng et al. [ 42 ]	44	65	10	Self‐reported LTPA	High vs. low‐intensity LTPA Moderate vs. low‐intensity LTPA	CVD mortality	0.73 (0.69–0.77) 0.77 (0.74–0.81)	Critically low
Ekelund et al. [ 43 ]	16	NA	10.05	Self‐reported SB Self‐reported TV‐viewing time	High vs. low SB High vs. low TV‐viewing time High vs. low SB High vs. low TV‐viewing time High vs. low SB High vs. low TV‐viewing time	All‐cause mortality CVD mortality Cancer mortality	1.27 (1.22–1.32) 1.44 (1.34–1.56) 1.74 (1.60–1.90) 2.26 (1.93–2.66) 1.22 (1.13–1.31) 1.26 (1.10–1.45)	Critically low
Ekelund et al. [ 44 ]	8	62.6	5.8	Device‐measured TPA Device‐measured SB	High vs. low TPA High vs. moderate to vigorous TPA High vs. low SB	All‐cause mortality	0.27 (0.23–0.32) 0.52 (0.43–0.61) 2.63 (1.94–3.56)	High
Ekelund et al. [ 45 ]	9	NA	10.2	Self‐reported SB Self‐reported TV‐viewing time	High vs. low SB High vs. low TV‐viewing time High vs. low SB High vs. low TV‐viewing time	CVD mortality Cancer mortality	1.32 (0.21–1.43) 1.59 (1.38–1.80) 1.21 (1.14–1.28) 1.29 (1.10–1.51)	Critically low
Garcia et al. [ 46 ]	191	NA	> 3	Self‐reported LTPA	High vs. very low LTPA Moderate vs. very low LTPA Low vs. very low LTPA High vs. very low LTPA Moderate vs. very low LTPA Low vs. very low LTPA High vs. very low LTPA Moderate vs. very low LTPA Low vs. very low LTPA	All‐cause mortality CVD mortality Cancer mortality	0.66 (0.62–0.70) 0.69 (0.65–0.73) 0.77 (0.73–0.80) 0.65 (0.60–0.71) 0.71 (0.66–0.77) 0.81 (0.77–0.85) 0.82 (0.77–0.86) 0.85 (0.81–0.89) 0.90 (0.88–0.93)	High
Grøntved and Hu [ 47 ]	8	20–89	8.61	Self‐reported TV viewing	TV‐viewing time (2 h/day)	All‐cause mortality	1.13 (1.07–1.18)	High
Hamer and Chida [ 48 ]	11	≥ 20	11.3	Self‐reported walking time	High vs. very low	All‐cause mortality	0.68 (0.59–0.78)	Moderate
Han et al. [ 49 ]	34	18–90	15	Device‐measured CRF	Per one‐MET increase in CRF High vs. low CRF Moderate vs. low CRF	All‐cause mortality CVD mortality Cancer mortality All‐cause mortality CVD mortality Cancer mortality All‐cause mortality CVD mortality Cancer mortality	0.88 (0.83–0.93) 0.87 (0.83–0.91) 0.93 (0.91–0.96) 0.47 (0.39–0.56) 0.49 (0.42–0.56) 0.57 (0.46–0.70) 0.67 (0.61–0.74) 0.60 (0.51–0.69) 0.76 (0.69–0.84)	Low
Hupin et al. [ 50 ]	9	60	9.8	Self‐reported TPA	High vs. very low TPA Moderate vs. very low TPA Low vs. very low TPA	All‐cause mortality	0.65 (0.61–0.70) 0.72 (0.65–0.80) 0.78 (0.71–0.87)	Critically low
Jayedi et al. [51]	7	≥ 20	7.7	Device‐measured daily steps	Per 1000 steps increment	All‐cause mortality	0.88 (0.83–0.93)	High
Kelly et al. [52]	18	NA	NR	Self‐reported walking time Self‐reported cycling time	International PA recommendations (11.25 MET. Hours per week)	All‐cause mortality All‐cause mortality	0.89 (0.83–0.96) 0.90 (0.87–0.94)	High
Kodama et al. [ 53 ]	33	37–57	10.43	Device‐measured CRF	Per one‐MET increase in CRF	All‐cause mortality CVD mortality	0.87 (0.84–0.90) 0.85 (0.82–0.88)	High
Ku et al. [ 54 ]	11	60.7	6.2	Device‐measured TPA	High vs. very low TPA low vs. very moderate vs. very low TPA Low TPA	All‐cause mortality	0.56 (0.44–0.71) 0.68 (0.59–0.79) 0.71 (0.62–0.82)	High
Liew et al. [ 55 ]	20	40–85	6.05	Device‐measured TPA Device‐measured SB	High vs. low TPA High vs. moderate‐to‐vigorous TPA High vs. very low TPA High vs. low SB High vs. low TPA High vs. moderate‐to‐vigorous TPA High vs. very low TPA High vs. low SB	All‐cause mortality CVD mortality	0.42 (0.34–0.53) 0.43 (0.35–0.53) 0.58 (0.43–0.80) 1.58 (1.19–2.09) 0.29 (0.18–0.47) 0.37 (0.25–0.55) 0.62 (0.41–0.93) 1.89 (1.09, 3.29)	Moderate
Liu et al. ^a^ [56]	9	54.7	14.45	Self‐reported LTPA	High vs. very low LTPA Moderate vs. very low LTPA Low vs. very low LTPA High vs. very low LTPA Moderate vs. very low LTPA Low vs. very low LTPA High vs. very low LTPA Moderate vs. very low LTPA Low vs. very low LTPA	All‐cause mortality CVD mortality Cancer mortality	0.86 (0.82–0.91) 0.86 (0.81–0.92) 0.85 (0.81–0.90) 0.84 (0.77–0.92) 0.86 (0.78–0.94) 0.83 (0.78–0.89) 0.93 (0.89–0.98) 0.94 (0.87–1.00) 0.92 (0.88–0.95)	Critically low
Löllgen et al. [57]	38	30	14.45	Self‐reported TPA	Highly active vs. inactive population Moderately active vs. inactive population	All‐cause mortality	0.80 (0.66–0.97) 0.78 (0.61–1.00)	Low
Merom et al. ^a^ [ 58 ]	11	**≥** 40	9.7	Self‐reported walking time Self‐reported dancing time	Moderate walking vs. inactive population Low walking vs. inactive population Moderate dancing vs. inactive population Low dancing vs. inactive population	CVD mortality	0.47 (0.29–0.75) 0.86 (0.65–1.13) 0.35 (0.27–0.45) 0.81 (0.69–0.95)	Critically low
Momma et al. [59]	16	**≥** 18	25.2	Self‐reported Resistance training	Aerobic only RT vs. inactive population RT plus AT vs. inactive population	All‐cause mortality CVD mortality Cancer mortality	0.75 (0.67–0.84) 0.85 (0.79–0.93) 0.60 (0.54–0.67) 0.71 (0.61–0.81) 0.83 (0.73–0.93) 0.54 (0.41–0.70) 0.89 (0.72–1.10) 0.88 (0.80–0.97) 0.72 (0.53–0.98)	High
Moore et al. ^a^ [ 60 ]	6	21–90	10	Self‐reported LTPA	High vs. inactive population Moderate vs. inactive population Low vs. inactive population	All‐cause mortality	0.59 (0.57–0.61) 0.61 (0.59–0.63) 0.68 (0.66–0.69)	Critically low
Nascimento et al. [ 61 ]	12	NA	15.5	Self‐reported resistance training and LTPA	RT vs. inactive population LTPA vs. inactive population RT plus LTPA vs. inactive population	Cancer mortality	0.83 (0.73–0.94) 0.89 (0.72–1.10) 0.72 (0.53–0.98)	Moderate
Nocon et al. [ 62 ]	33	12.6–94	12	Self‐reported CRF Device‐measured CRF	High vs. low CRF High vs. low CRF High vs. low CRF High vs. low CRF	All‐cause mortality CVD mortality	0.71 (0.66–0.76) 0.59 (0.53–0.65) 0.70 (0.66–0.74) 0.43 (0.33–0.57)	Critically low
O'Donovan et al. ^a^ [ 63 ]	11	58.6	8.8	Self‐reported LTPA	High vs. very low LTPA Moderate vs. very low LTPA Low vs. very low LTPA	All‐cause mortality CVD mortality Cancer mortality	0.65 (0.58–0.73) 0.70 (0.60–0.82) 0.69 (0.65–0.74) 0.59 (0.48–0.73) 0.60 (0.45–0.82) 0.63 (0.55–0.72) 0.79 (0.66–0.94) 0.82 (0.63–1.06) 0.86 (0.77–0.96)	High
Paluch et al. [ 64 ]	15	≥ 18	7.1	Device‐measured daily steps	High vs. very low Moderate vs. very low Low vs. very low	All‐cause mortality	0.39 (0.32–0.48) 0.47 (0.40–0.56) 0.56 (0.47–0.65)	High
Pedisic et al. [ 65 ]	6	≥ 18	20.25	Self‐reports running time	High vs. no running	All‐cause mortality CVD mortality Cancer mortality	0.73 (0.68–0.79) 0.70 (0.49–0.98) 0.77 (0.68–0.87)	High
Qiu et al. [ 66 ]	25	39.9–71.1	16.4	Self‐reported CRF	Per 1‐MET higher of CRF	All‐cause mortality CVD mortality	0.83 (0.78–0.88) 0.83 (0.80–0.86)	High
Ramakrishnan et al. [ 67 ]	15	≥ 18	8.25	Device‐measured TPA	High vs. low TPA	All‐cause mortality	0.33 (0.25–0.43)	Moderate
Rojer et al. [ 68 ]	12	≥ 60	2–9.8	Device‐measured SB Device‐measured daily steps	High vs. low SB High vs. low	All‐cause mortality	2.44 (1.82–3.25) 3.09 (2.33–4.11)	High
Runacres et al. [69]	44	≥ 60	5.9	Elite athletes	General population	All‐cause mortality CVD mortality Cancer mortality	0.67 (0.59–0.75) 0.73 (0.62–0.85) 0.75 (0.63–0.89)	High
Saeidifard et al. [70]	11	≥ 18	8.8	Self‐reported resistance training and LTPA	RT vs. inactive population LTPA vs. inactive population RT plus LTPA vs. inactive population RT vs. inactive population RT plus LTPA vs. inactive population RT vs. inactive population RT plus LTPA vs. inactive population	All‐cause mortality CVD mortality Cancer mortality	0.79 (0.69–0.91) 0.59 (0.45–0.76) 0.60 (0.49–0.72) 0.83 (0.68–1.03) 0.43 (0.27–0.70) 0.81 (0.54–1.20) 0.90 (0.75–1.08)	Moderate
Samitz et al. [ 71 ]	33	56.4	10.7	Self‐reported TPA Self‐reported LTPA Self‐reported OPA	High vs. low TPA High vs. low LTPA High vs. low OPA	All‐cause mortality	0.85 (0.77–0.94) 0.74 (0.70–0.77) 0.83 (0.71–0.97)	High
Schmidand Leitzmann [ 72 ]	6	NA	16.4	Device‐measured CRF	High vs. low CRF Moderate vs. low CRF	Cancer mortality	0.80 (0.67–0.97) 0.55 (0.47–0.65)	High
Shailendra et al. [ 73 ]	10	≥18	1–7	Self‐reported resistance training and LTPA	RT vs. inactive population LTPA vs. inactive population RT plus LTPA vs. inactive population RT vs. inactive population LTPA vs. inactive population RT plus LTPA vs. inactive population RT vs. inactive population LTPA vs. inactive population RT plus LTPA vs. inactive population	All‐cause mortality CVD mortality Cancer mortality	0.82 (0.72–0.93) 0.75 (0.67–0.84) 0.60 (0.54–0.66) 0.82 (0.74–0.91) 0.71 (0.61–0.81) 0.54 (0.41–0.70) 0.84 (0.75–0.94) 0.89 (0.72–1.10) 0.72 (0.53–0.98)	High
Sheng et al. [ 74 ]	16	≥ 40	6.4	Device‐measured daily steps	High vs. low	All‐cause mortality CVD mortality	0.31 (0.23–0.42) 0.41 (0.25–0.67)	High
Stamatakis et al. [ 75 ]	11	≥ 30	9.2	Self‐reported resistance training	RT vs. inactive population	All‐cause mortality Cancer mortality	0.77 (0.69–0.87) 0.69 (0.56–0.86)	Critically low
Stens et al. [76]	12	≥ 18	6.5	Device‐measured daily steps	High vs. low Moderate vs. low	All‐cause mortality	0.50 (0.42–0.60) 0.64 (0.56–0.72)	High
Sun et al. [ 77 ]	10	NA	10	Self‐reported TV viewing	High vs. low TV‐viewing time	All‐cause mortality	1.33 (1.20–1.47)	High
Takagi et al. [ 78 ]	15	37.3–64.8	9.52	Self‐reported TV viewing	High vs. low TV‐viewing time	CVD mortality	1.32 (1.12–1.55)	Moderate
Wang et al. [ 79 ]	16	43.9–93.5	3–14.4	Muscle wasting	Low vs. normal muscle mass	All‐cause mortality	1.57 (1.25–1.96)	High
Wilmot et al. [ 80 ]	18	≥ 18	12	Self‐reported SB	High vs. low SB	All‐cause mortality CVD mortality	1.49 (1.14–2.03) 1.90 (1.36–2.66)	High
Woodcock et al. [81]	22	20–88	11.9	Self‐reported TPA Self‐reported walking time	11 vs. 0 MET‐h/week	All‐cause mortality	0.81 (0.76–0.85) 0.89 (0.82–0.96)	Critically low
Zhou et al. [82]	49	38.7–93.5	2.5–32	Muscle wasting	Low vs. normal muscle mass	All‐cause mortality CVD mortality Cancer mortality	1.36 (1.59–1.75) 1.29 (1.05–1.58) 1.75 (1.02–1.27)	High

Abbreviations: CI; coefficient intervals; CRF, cardiorespiratory fitness; CVD, cardiovascular disease; HR, hazard ratio; *k*, number of included studies; LTPA, leisure‐time physical activity; MET, metabolic equivalent task; NA, not applicable; NR, not reported; OPA, occupational physical activity; PA, physical activity; RT, resistance training; SB, sedentary behaviour; TPA, total physical activity.

^a^ Showing pooled analysis studies.

### 3.1 Summary of Associations

Of the 35 examined meta‐analytical associations, 28 (80%) had a nominally statistically significant finding (*p* < 0.05) under the random‐effects models, but none of those reached a *p* value of 10^−6^ or less. All meta‐analytical associations had more than 1000 cases for continuous outcomes. Twenty‐five meta‐analytical associations (72%) exhibited large heterogeneity (*I* ^2^ > 50%), and only 13 of them (38%) had a 95% PI that excluded the null value. Additionally, small study effects were found for only four meta‐analytical associations (11%), and excess significance bias was found for seven studies. None of the associations reached a convincing (Class I) or highly suggestive (Class II) level of evidence. For the remaining associations, 11 (31.5%) showed suggestive evidence (Class III). These included associations between self‐reported LTPA and mortality from all‐causes, CVD and cancers; self‐reported TPA and mortality from all‐causes; device‐measured TPA and daily steps and mortality from all‐causes; results from pooled studies and mortality from all‐causes, CVD and cancers; resistance training and mortality from CVD; and aerobic plus resistance training and mortality from all‐causes.

Seventeen associations (48.5%) showed weak evidence (class IV). These included associations between self‐reported SB and mortality from all‐causes, CVD and cancers; device‐measured SB and mortality from all‐causes; self‐reported walking time and mortality from all‐causes; self‐reported TV viewing and mortality from cancers; resistance training and mortality from all‐causes and cancers; aerobic training and mortality from all‐causes; aerobic plus resistance training and mortality from CVD and cancers; device‐measured CRF and mortality from all‐causes, CVD and cancers; CRF per one‐MET increase in CRF and mortality from all‐causes and CVD; and PA and the incidence of cardiovascular disease.

Finally, seven associations (20%) had no evidence (not significant). These included associations between self‐reported running time and mortality from all‐causes, CVD and cancers; low skeletal muscle mass and mortality from all‐causes; device‐measured daily steps per 1000‐step increment and mortality from all causes; and self‐reported TV viewing and mortality from all‐causes and CVD. The detail for the classification of the level of evidence is presented in the Supporting Information (Table S4).

### 3.2 All‐Cause Mortality

**FIGURE 2:**
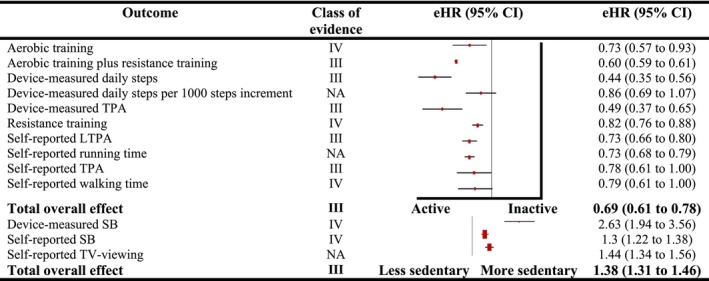
Observational meta‐analytical associations between different domains of physical activity and sedentary behaviour with mortality from all causes in general population. LTPA, leisure‐time physical activity; NA, not applicable; SB, sedentary behaviour; TPA, total physical activity. Class of evidence: suggestive (III) and weak (IV).

Overall, suggestive evidence indicates that any form of PA and SB was significantly associated with lower and higher hazards of all‐cause mortality (eHR = 0.69 [95% confidence interval 0.61–0.78], *p* = 0.00001, and eHR = 1.38 [1.31–1.46], *p* = 0.00001) (Figure 2). The association between self‐reported TPA (eHR = 0.78 [0.70–0.86]), device‐measured TPA (eHR = 0.47 [0.35–0.63]), self‐reported LTPA (eHR = 0.73 [0.66–0.80]), device‐measured daily steps (eHR = 0.44 [0.35–0.56]) and aerobic plus resistance training (eHR = 0.60 [0.56–0.64]) with all‐cause mortality was supported by suggestive evidence. Weak evidence was noted for the association between self‐reported SB (eHR = 1.3 [1.22–1.38]), device‐measured SB (eHR = 2.16 [1.09–4.28]), self‐reported walking time (eHR = 0.79 [0.61–1.00]), resistance training (eHR = 0.82 [0.76–0.88]), aerobic training (eHR = 0.73 [0.57–0.93]), device‐measured CRF (eHR = 0.58 [0.38–0.89]) and CRF per one‐MET increase in CRF (eHR = 0.87 [0.82–0.93]) with all‐cause mortality. The evidence did not reach to the significant level for the association between self‐reported running time (eHR = 0.78 [0.35–1.76]), device‐measured daily steps per 1000 steps increment (eHR = 0.86 [0.69–1.07]), self‐reported TV‐viewing (eHR = 1.27 [0.27–5.94]) and low skeletal muscle mass (eHR = 1.40 [0.67–2.92]) with all‐cause mortality. The summary estimates in the meta‐analyses of the pooled studies indicated suggestive evidence for the association between TPA and LTPA with all‐cause mortality (eHR = 0.70 [0.64–0.76]) (Tables S5 and S6).

### 3.3 Cardiovascular Disease Mortality

**FIGURE 3:**
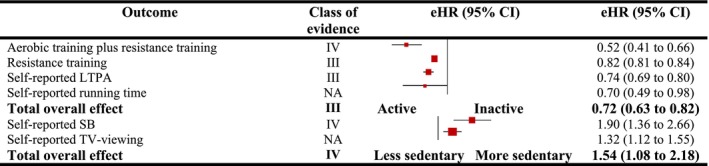
Observational meta‐analytical associations between different domains of physical activity and sedentary behaviour with mortality from CVD in general population. LTPA, leisure‐time physical activity; NA, not applicable; SB, sedentary behaviour. Class of evidence: suggestive (III) and weak (IV).

Our pooled analysis showed suggestive and weak evidence implicating that any form of PA and SB was significantly associated with lower and higher hazards of CVD mortality (eHR = 0.72 [0.63–0.82], *p* = 0.00001, and eHR = 1.54 [1.08–2.18], *p* = 0.02) (Figure 3). Evidence was suggestive for the associations between self‐reported LTPA (eHR = 0.74 [0.69–0.80] and resistance training (eHR = 0.82 [0.81–0.84]) with CVD mortality. Weak evidence was found for the associations between self‐reported SB (eHR = 1.47 [1.04–2.07]), aerobic plus resistance training (eHR = 0.52 [0.41–0.66]), device‐measured CRF (eHR = 0.51 [0.34–0.77]) and CRF per one‐MET increase in CRF (eHR = 0.86 [0.74–0.99]) with CVD mortality. Weak evidence was also noted for the associations PA and the incidence of cardiovascular disease (eHR = 0.55 [0.31–1.00]). The evidence did not reach to the significant level for the association between self‐reported running time (eHR = 0.81 [0.62–1.05]) and self‐reported TV‐viewing (eHR = 1.68 [0.85–3.30]) with CVD mortality. Additionally, the evidence was suggestive in the meta‐analyses of the pooled studies for the association between TPA, LTPA, walking time and dancing time with CVD mortality (eHR = 0.69 [0.61–0.78]) (Table S5).

### 3.4 Cancer Mortality

**FIGURE 4:**
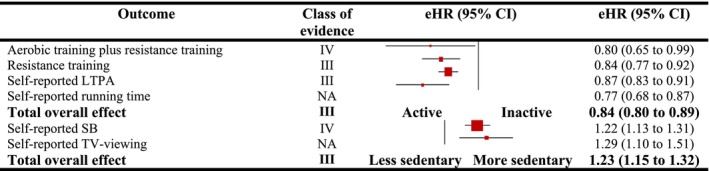
Observational meta‐analytical associations between different domains of physical activity and sedentary behaviour with mortality from cancer in general population. LTPA, leisure‐time physical activity; NA, not applicable; SB, sedentary behaviour. Class of evidence: suggestive (III) and weak (IV).

The random‐effect model showed that any form of PA and SB was significantly associated with lower and higher hazards of cancer mortality (eHR = 0.84 [0.80–0.89], *p* = 0.00001, and eHR = 1.23 [1.15–1.32], *p* = 0.00001) (Figure 4). Suggestive evidence was found for the association between self‐reported LTPA (eHR = 0.87 [0.83–0.91]) with cancer mortality. However, weak evidence was noted for the associations between self‐reported SB (eHR = 1.47 [1.04–2.07]), self‐reported TV‐viewing (eHR = 1.27 [1.10–1.48]), resistance training (eHR = 0.84 [0.77–0.92]), aerobic plus resistance training (eHR = 0.80 [0.65–0.99]) and device‐measured CRF (eHR = 0.67 [0.49–0.90]) with cancer mortality. The evidence did not reach to the significant level for the association between self‐reported running time (eHR = 0.84 [0.34–2.07]) with cancer mortality. The evidence was also suggestive in the meta‐analyses of the pooled studies for the association between TPA and LTPA with cancer mortality (eHR = 0.84 [0.78–0.90]) (Table S5).

### 3.5 The Role of Age on the Relationship Between Physical Activity and Mortality

Meta‐regression analyses were conducted to examine the moderating role of age on the relationship between physical activity and mortality due to all‐cause mortality, CVD and cancer. The analysis revealed a significant negative association between age and the magnitude of the protective effect of physical activity on mortality across all types of mortality in individuals who engage in physical activity, as observed in the meta‐analyses of the pooled studies. Specifically, for all‐cause mortality, the slope was −0.042 (95% CI: −0.059 to −0.026, *p* < 0.00001, Figure S1), indicating that for each 1‐year increase in age, the protective effect of physical activity on all‐cause mortality risk decreases by approximately 4.2%. Similarly, for CVD mortality, the slope was −0.079 (95% CI: −0.010 to −0.050, *p* < 0.00001, Figure S2), suggesting that for each year increase in age, the protective effect of physical activity on CVD mortality risk decreases by 7.9%. Lastly, for cancer mortality, the slope was −0.025 (95% CI: −0.049 to −0.002, *p* < 0.03, Figure S3), showing that for each year increase in age, the protective effect of physical activity on cancer mortality risk decreases by approximately 2.5%. These findings suggest that age is an effect modifier, where physical activity has a greater protective effect on mortality in younger adults compared to older adults. As people age, the likelihood of mortality increases even among those who engage in physical activity, which reduces the observed difference in mortality risk between physically active and inactive individuals. These results emphasize the importance of maintaining physical activity across the lifespan, particularly in older adults, as a strategy to mitigate the risk of mortality from various causes.

### 3.6 Dose–Response Relationship Between Physical Activity and Mortality

Subgroup analysis based on the dose–response relationship indicated that higher level of LTPA were significantly associated with lower hazards of all‐cause mortality (high vs. inactive: eHR = 0.73 [0.66–0.80], *I* ^2^ = 80%, *p* < 0.0001; moderate vs. inactive: eHR = 0.69 [0.65–0.73], *p* < 0.0001; low vs. inactive: eHR = 0.77 [0.73–0.81], *p* < 0.0001; Figure S4) and CVD mortality (high vs. inactive: eHR = 0.71 [0.66–0.77], *I* ^2^ = 55%, *p* < 0.0001; moderate vs. inactive: eHR = 0.74 [0.69–0.81], *p* < 0.0001; low vs. inactive: eHR = 0.81 [0.77–0.85], *p* < 0.0001; Figure S5). We observed a trend indicating that higher levels of LTPA are associated with a lower hazards of cancer mortality (high vs. inactive: eHR = 0.84 [0.79–0.90], *I* ^2^ = 14%, *p* < 0.0001; moderate vs. inactive: eHR = 0.85 [0.81–0.89], *p* < 0.0001; low vs. inactive: eHR = 0.90 [0.87–0.93], *p* < 0.0001; Figure S6). Subgroup analysis based on the dose–response relationship showed that higher level of device‐measured TPA (Figure S7), but not self‐reported TPA (Figure S8), was significantly associated with lower hazards of all‐cause mortality (device‐measured TPA; high vs. inactive: eHR = 0.38 [0.27–0.53], *I* ^2^ = 89%, *p* < 0.0001; moderate vs. inactive: eHR = 0.54 [0.35–0.85], *I* ^2^ = 92%, *p* = 0.008; low vs. inactive: eHR = 0.67 [0.57–0.80], *I* ^2^ = 27%, *p* < 0.0001; self‐reported TPA; high vs. inactive: eHR = 0.79 [0.66–0.94], *I* ^2^ = 96%, *p* < 0.0001; moderate vs. inactive: eHR = 0.73 [0.66–0.80], *I* ^2^ = 0%, *p* < 0.0001; low vs. inactive: eHR = 0.78 [0.70–0.87], *p* < 0.0001).

Subgroup analysis based on the dose–response relationship in pooled analysis studies revealed no association between higher level of PA and all‐cause mortality (high vs. inactive: eHR = 0.68 [0.58–0.80], *I* ^2^ = 97%, *p* < 0.0001; moderate vs. inactive: eHR = 0.71 [0.63–0.81], *I* ^2^ = 97%, *p* < 0.0001; low vs. inactive: eHR = 0.71 [0.64–0.77], *I* ^2^ = 95%, *p* < 0.0001; Figure S9), CVD mortality (high vs. inactive: eHR = 0.66 [0.50–0.88], *I* ^2^ = 96%, *p* = 0.004; moderate vs. inactive: eHR = 0.64 [0.49–0.84], *I* ^2^ = 94%, *p* = 0.001; low vs. inactive: eHR = 0.73 [0.63–0.84], *I* ^2^ = 91%, *p* < 0.0001; Figure S10) and cancer mortality (high vs. inactive: eHR = 0.82 [0.68–0.98], *I* ^2^ = 96%, *p* = 0.030; moderate vs. inactive: eHR = 0.83 [0.69–1.00], *I* ^2^ = 93%, *p* = 0.060; low vs. inactive: eHR = 0.85 [0.76–0.96], *I* ^2^ = 94%, *p* = 0.009; Figure S11).

However, subgroup analysis based on the different measurement method indicated that device‐measured TPA was significantly associated with lower hazards of all‐cause mortality, compared to self‐reported TPA (device‐measured TPA: eHR = 0.47 [0.36–0.63], *I* ^2^ = 94%, *p* < 0.0001; self‐reported TPA: eHR = 0.78 [0.69–0.87], *I* ^2^ = 93%, *p* < 0.0001; Figure S12). Meta‐regression analysis was conducted to examine the moderating role of the measurement method on the relationship between physical activity and mortality. The analysis revealed a significant interaction between the measurement method and the physical activity‐mortality association. Specifically, for all‐cause mortality, the slope for the interaction between self‐reported and device‐measured physical activity was −0.47 (95% CI: −0.55 to −0.37, *p* < 0.0001; Figure S13), suggesting that device‐measured physical activity has a stronger association with reduced mortality compared to self‐reported activity. This indicates that the method of measuring physical activity significantly moderates the relationship between physical activity and mortality risk. These findings underscore the importance of using accurate and objective measurement methods in studies examining the health benefits of physical activity, particularly in relation to mortality outcomes.

### 3.7 The Relationship Between Different Types of Exercise Training and Mortality

We performed another set of subgroup analysis to compare the relationship between resistance training, aerobic training and aerobic plus resistance training with mortality. We found that combined exercise training (aerobic plus resistance training) was the most appropriate form of exercise for reducing the hazards of all‐cause mortality (aerobic plus resistance training: eHR = 0.60 [0.56–0.64], *I* ^2^ = 0%, *p* < 0.0001; resistance training: eHR = 0.82 [0.77–0.86], *I* ^2^ = 0%, *p* < 0.0001; aerobic training: eHR = 0.73 [0.66–0.80], *I* ^2^ = 32%, *p* < 0.0001; Figure S14) and also CVD mortality (aerobic plus resistance training: eHR = 0.52 [0.43–0.63], *I* ^2^ = 0%, *p* < 0.0001; resistance training: eHR = 0.83 [0.77–0.89], *I* ^2^ = 0%, *p* < 0.0001; Figure S15). However, we did not find any significant difference between resistance training and combined exercise training for reducing the hazards of cancer mortality (aerobic plus resistance training: eHR = 0.81 [0.69–0.95], *I* ^2^ = 16%, *p* = 0.010; resistance training: eHR = 0.84 [0.79–0.89], *I* ^2^ = 5%, *p* < 0.0001; Figure S16).

### 3.8 Heterogeneity and Sensitivity Analyses

To explore the potential effects of different follow‐up durations on the relationship between physical activity and mortality, we performed a set of subgroup analyses and compared the hazards for follow‐up periods of less than 10 years and equal to or greater than 10 years. Only for the association between self‐reported SB with CVD mortality, higher follow‐up duration was associated with higher hazards of mortality (< 10 years: eHR = 1.18 [1.10–1.26]; ≥ 10 years: eHR = 1.59 [1.26–2.01]; Figure S17). There were no significant differences in the relationship between self‐reported LTPA and mortality (due to all‐cause mortality, CVD and cancer), self‐reported TPA and all‐cause mortality, self‐reported SB and mortality (due to all‐cause mortality, CVD and cancer) and in the meta‐analyses of pooled studies and mortality (due to all‐cause mortality, CVD and cancer) across different follow‐up durations (Figures S18–S27).

To evaluate the robustness of overall effect sizes, we conducted several analyses for sensitivity. First, for outcomes with heterogeneity exceeding 50%, we conducted *p*‐curve analysis, and the overall pattern of results remained consistent, indicating no substantial evidence of p‐hacking (Figures 28–37). This analysis further supports the conclusion that PA, SB, exercise training and mortality from all‐causes, CVD and cancers are not predominantly influenced by studies with exceptionally large effect sizes or small *p* values. Second, we removed studies with low and critically low quality and recalculated the overall effect sizes based on the remaining studies. The overall effect sizes for the following analysis remained stable: relationship between self‐reported LTPA and CVD mortality (before sensitivity analysis: eHR = 0.74 [0.69–0.80], *p* < 0.0001, suggestive evidence; after sensitivity analysis: eHR = 0.73 [0.65–0.83], *p* = 0.002, suggestive evidence; Figure S38), relationship between device‐measured TPA and all‐cause mortality (before sensitivity analysis: eHR = 0.49 [0.37–0.65], *p* = 0.0003, suggestive evidence; after sensitivity analysis: eHR = 0.52 [0.40–0.67], *p* = 0.001, suggestive evidence; Figure S39), relationship between self‐reported SB and all‐cause mortality (before sensitivity analysis: eHR = 1.3 [1.22–1.38], *p* = 0.01, weak evidence; after sensitivity analysis: eHR = 1.33 [1.22–1.45], *p* = 0.005, suggestive evidence; Figure S40), relationship between device‐measured SB and all‐cause mortality (before sensitivity analysis: eHR = 2.16 [1.09–4.28], *p* = 0.04, weak evidence; after sensitivity analysis: eHR = 2.53 [1.57–4.07], *p* = 0.026, weak evidence; Figure S41), relationship between PA and all‐cause mortality in pooled analysis studies (before sensitivity analysis: eHR = 0.70 [0.64–0.76], *p* < 0.0001, suggestive evidence; after sensitivity analysis: eHR = 0.64 [0.55–0.74], *p* < 0.001, suggestive evidence; Figure S42), relationship between PA and CVD mortality in pooled analysis studies (before sensitivity analysis: eHR = 0.69 [0.61–0.78], *p* < 0.0001, suggestive evidence; after sensitivity analysis: eHR = 0.62 [0.56–0.67], *p* < 0.002, suggestive evidence; Figure S43), relationship between PA and cancer mortality in pooled analysis studies (before sensitivity analysis: eHR = 0.84 [0.78–0.90], *p* = 0.0006, suggestive evidence; after sensitivity analysis: eHR = 0.84 [0.75–0.94], *p* = 0.021, weak evidence; Figure S44), relationship between resistance training and all‐cause mortality (before sensitivity analysis: eHR = 0.82 [0.76–0.88], *p* = 0.03, weak evidence; after sensitivity analysis: eHR = 0.83 [0.76–0.91], *p* = 0.012, weak evidence; Figure S45) and relationship between resistance training and cancer mortality (before sensitivity analysis: eHR = 0.84 [0.77–0.92], *p* = 0.005, weak evidence; after sensitivity analysis: eHR = 0.85 [0.81–0.90], *p* = 0.002, suggestive evidence; Figure S46).

However, the relationship between self‐reported TPA and all‐cause mortality (before sensitivity analysis: eHR = 0.78 [0.70–0.86], *p* = 0.0008, suggestive evidence; after sensitivity analysis: eHR = 0.88 [0.77–1.00], *p* = 0.051; Figure S47) changed to a nonsignificant level after the sensitivity analysis. The relationship between self‐reported SB and CVD mortality (before sensitivity analysis: eHR = 1.90 [1.36–2.66], *p* = 0.03, weak evidence; after sensitivity analysis: eHR = 1.32 [0.83–2.12], *p* = 0.126; Figure S48) also became nonsignificant. Similarly, the relationship between self‐reported SB and cancer mortality (before sensitivity analysis: eHR = 1.22 [1.13–1.21], *p* = 0.049, weak evidence; after sensitivity analysis: eHR = 1.19 [0.96–1.47], *p* = 0.06; Figure S49) turned nonsignificant. Finally, the relationship between self‐reported walking time and all‐cause mortality (before sensitivity analysis: eHR = 0.79 [0.61–1.00], *p* = 0.0008, suggestive evidence; after sensitivity analysis: eHR = 0.88 [0.77–1.00], *p* = 0.051; Figure S50) also became nonsignificant after the sensitivity analysis, which removed studies of low and critically low quality. Third, to facilitate the identification of potential outliers, a Baujat plot was generated to present each study's contribution to heterogeneity and publication bias. The results indicated that no study contributed significantly to heterogeneity or publication bias (Figures S51–S63).

## 4 Discussion

This umbrella review graded the credibility and certainty of evidence on the association between different domains of PA and SB with mortality from all causes, CVD and cancer, encompassing observational evidence.

Overall, PA and SB had lower and higher associations with mortality from all causes, CVD and cancers. Evidence from observational studies (high credibility and suggestive certainty) show an association between self‐reported TPA, device‐measured TPA, self‐reported LTPA and self‐reported SB with all‐cause mortality and an association between self‐reported LTPA and resistance training with CVD mortality. Observational evidence with high credibility and a certainty suggests an association between device‐measured SB, resistance training, aerobic training, aerobic plus resistance training, device‐measured CRF and CRF per one‐MET increase in CRF with all‐cause mortality; an association between self‐reported SB, aerobic plus resistance training, device‐measured CRF and CRF per one‐MET increase in CRF with CVD mortality; and an association between self‐reported SB, self‐reported TV‐viewing, resistance training, aerobic plus resistance training and device‐measured CRF with cancer mortality.

Importantly, 7.2% and 7.6% of all‐cause and CVD mortality were attributed to physical inactivity, respectively [83]. Using a population‐attributable fraction formula to estimate the direct public health‐care costs of noncommunicable diseases (NCDs) for 2020–30 has shown that if the prevalence of physical inactivity does not change, 499.2 million new cases of preventable major NCDs would occur globally by 2030 and the global cost of inaction on physical inactivity would reach approximately $47.6 billion per year [84]. According to the Global Burden of Disease 2019 study, LPA was associated with 198.42 (95% uncertainty interval 108.16–360.32) disability adjusted life years per 100 000 individuals and 11.1 (95% uncertainty interval 5.66–19.51) death rates per 100 000 individuals globally [85]. The analysis of burden of disease and life expectancy in 2012 has been indicated that physical inactivity causes 6%–10% of the major NCDs including coronary heart disease, type 2 diabetes, and breast and colon cancers. More importantly, by elimination of physical inactivity, the global life expectancy might be expected to increase by 0.68 years [2].

There is limited biological mechanistic information about the health benefits of different domains of PA. It has been documented that PA influences blood pressure, lipid levels, glucose tolerance and body mass index. Moreover, PA can directly affect the function and structure of the vascular system and may help reduce cardiovascular risk. PA can also improve endothelial cell function, reduce plaque progression, stabilizing the induced plaques, decrease myocardial oxygen demand and alleviate thrombosis [84, 86, 87, 88].

In our analysis, we observed a notable difference between the studies using self‐reported questionnaires and those utilizing objective tools such as accelerometers for measuring PA levels. Specifically, studies relying on questionnaires reported much larger effect sizes and more favourable health benefits compared to those using objective tools. This discrepancy could be attributed to biases associated with self‐reported data, such as recall bias or social desirability bias, which might overestimate the associations between PA and health outcomes. Given these findings, we recommend that future studies in this area carefully consider the differences between self‐reported and objective measurement methods when analysing the impact of PA and SB on health outcomes. Employing both types of measurement tools in parallel could provide a more robust understanding of these relationships and help mitigate the biases introduced by self‐reporting.

### 4.1 Implications and Future Research

Given that CVD and cancers are the leading causes of mortality [1], the findings of our umbrella review may have important implications for public health and provide support for the current recommended public health guidelines. Public health policy makers and researchers should consider this evidence synthesis when making policy decisions on PA recommendation and can inform future guideline development by also recognizing the role of PA for reducing the risk of premature death. Future PA guidelines are needed to translate current evidence synthesis findings into clinical practice, while involving stakeholders. We only investigated the associations of PA with mortality from all‐causes, CVD and cancers. Thus, more evidence synthesis studies are needed to determine the associations between PA and SB with cause specific mortality and for other chronic morbidities such as type 2 diabetes, obesity and hypertension, which may manifest at a younger age. Finally, data on the association between PA and SB with mortality from low‐ and middle‐income countries are currently unavailable.

### 4.2 Strengths and Limitations

This umbrella review is the first to pool meta‐analyses of observational studies on the association between domains of PA and SB with mortality from all causes, CVD and cancer, accounting for PA measurement tools and rating findings' credibility based on established criteria. We also separated studies based on the two current approaches for the measurement of PA. It has been documented that the ability of questionnaires and accelerometers to capture PA differs, and the relationship between PA and mortality was weaker in questionnaire‐based studies than in accelerometer based studies [89].

The results of this umbrella review should be interpreted in light of its limitations. First, the evidence from observational studies was heterogeneous with regards of the comparison methods. Our analyses were based mainly on the comparison between most active and least active groups. However, in some observational included studies they compared the recommended (by public health authorities) level of PA with higher or lower levels. A limitation of this approaches is that the criteria for measurement and classification of PA across studies are heterogeneous and might not be comparable. Second, another reason to be cautious is that umbrella reviews neglect evidence from individual studies that have not been previously pooled in meta‐analyses. Third, some confounding factors could drive associations in observational findings. However, we have applied stringent criteria, as confirmed by downgrading convincing evidence to nonsignificant on the association between PA and mortality outcomes. To control the effects of confounding factors in this umbrella review, we applied quantitative criteria to grade evidence from observational evidence accounted for selection and publication bias, excess of significance driven by small studies with larger effect sizes than the largest study in the meta‐analysis or marginal statistical significance driven by large sample sizes. Additionally, we discussed findings from observational evidence in the context of converging evidence from different sources of evidence considering many different aspects of PA and SB. Fourthly, excess of significance bias testing might have been underpowered in meta‐analyses that have included a small number of studies, which could arguably apply to all meta‐analyses included in the present umbrella review, yet a specific threshold of number of studies to set logical power of excess of significance bias has not been established. Fifthly, we could have included meta‐analyses by prioritizing their quality rather than the quantity of studies. However, that would have introduced a selection bias, resulting in the exclusion of a substantial body of evidence. Sixth, to harmonize effect sizes, we computed the corresponding hazard ratio as a measure of strength of the association. However, this harmonization process comes at the cost of losing information on time‐to‐event analyses. It is important to note that any association should be considered more in depth considering the frequency of each outcome and the follow‐up duration and time to event occurrence in each of the included studies. Moreover, the number of mortalities observed over the overall population of included studies does not reflect the prevalence of outcomes of interest. Seventh, it is crucial to highlight that the findings of this umbrella review are intended to inform future guidelines. These guidelines should take into consideration various additional factors, including cost‐effectiveness considerations, clinical relevancies, dose–response relationship between different levels of activity and mortality outcomes and long‐term effects of PA and SB where evidence is currently insufficient. Eighthly, the prospective studies we included did not provide the detailed subgroup data based on age, sex, socioeconomic status or preexisting health conditions, which restricted our capacity to investigate how associations between exercise training, PA, SB and mortality outcomes might vary across different populations. Future research should aim to address this gap by incorporating more granular demographic and health‐related information, enabling subgroup analyses that could provide more targeted insights for public health recommendations and interventions. Ninth, another limitation of this study is the absence of restricted cubic spline modelling for dose–response relationships, which could have provided a more nuanced understanding of the association between physical activity, sedentary behaviour and mortality outcomes. Finally, this study did not examine morbidity outcomes, quality of life measures or specific disease incidence rates. Future research should aim to explore these aspects in order to provide a more comprehensive understanding of the health impacts of PA, SB and different types of exercise interventions.

## 5 Conclusions

Converging evidence supports that PA and SB are associated with lower and higher rates of all‐cause, cardiovascular and cancer mortality. Although PA and SB as risk factors for premature mortality outcomes have been extensively studied for decades, only 11 of the 34 (32%) associations reported here were supported by suggestive evidence. Importantly, weak evidence was noted for 16 of the 34 (47%) associations. More high‐quality prospective studies are needed for a better understanding of the associations between PA and SB and mortality outcomes.

## Conflicts of Interest

The authors declare no conflicts of interest.

## Supporting information


**Table S1** PRIOR checklist.
**Table S2** PRISMA 2020 abstract checklist adapted for umbrella reviews.
**Table S3** Studies excluded, with reason for exclusion.
**Table S4** Quality assessment and publication bias evaluation of included study using AMSTAR 2.
**Table S5** Finding across meta‐analyses of observational studies on health outcomes of physical activity and sedentary behavior.
**Table S6** Finding across individual meta‐analyses of included observational studies.
**Figure S1** Meta‐regression analysis for the association between physical activity and all‐cause mortality based on the moderating role of age.
**Figure S2** Meta‐regression analysis for the association between physical activity and CVD mortality based on the moderating role of age.
**Figure S3** Meta‐regression analysis for the association between physical activity and cancer mortality based on the moderating role of age.
**Figure S4** Forest plot of the association between different level of LTPA and all‐cause mortality.
**Figure S5** Forest plot of the association between different level of LTPA and CVD mortality.
**Figure S6** Forest plot of the association between different level of LTPA and cancer mortality.
**Figure S7** Forest plot of the association between different level of device‐measured TPA and cancer mortality.
**Figure S8** Forest plot of the association between different level of self‐reported TPA and cancer mortality.
**Figure S9** Forest plot of the association between different level of PA and all‐cause mortality in pooled analysis studies.
**Figure S10** Forest plot of the association between different level of PA and CVD mortality in pooled analysis studies.
**Figure S11** Forest plot of the association between different level of PA and cancer mortality in pooled analysis studies.
**Figure S12** Forest plot of the association between different measurement methods and all‐cause mortality in pooled analysis studies.
**Figure S13** Meta‐regression analysis for the association between total physical activity and cancer mortality based on the moderating role of measurement methods.
**Figure S14** Forest plot of the association between different exercise training and all‐cause mortality.
**Figure S15** Forest plot of the association between different exercise training and CVD mortality.
**Figure S16** Forest plot of the association between different exercise training and cancer mortality.
**Figure S17** Forest plot of the association between self‐reported SB with CVD mortality based on different follow‐up duration.
**Figure S18** Forest plot of the association between self‐reported LTPA with all‐cause mortality based on different follow‐up duration.
**Figure S19** Forest plot of the association between self‐reported LTPA with CVD mortality based on different follow‐up duration.
**Figure S20** Forest plot of the association between self‐reported LTPA with cancer mortality based on different follow‐up duration.
**Figure S21** Forest plot of the association between self‐reported TPA with all‐cause mortality based on different follow‐up duration.
**Figure S22** Forest plot of the association between self‐reported SB with all‐cause mortality based on different follow‐up duration.
**Figure S23** Forest plot of the association between self‐reported SB with CVD mortality based on different follow‐up duration.
**Figure S24** Forest plot of the association between self‐reported SB with cancer mortality based on different follow‐up duration.
**Figure S25** Forest plot of the association between self‐reported PA with all‐cause mortality based on different follow‐up duration in pooled analysis studies.
**Figure S26** Forest plot of the association between self‐reported PA with CVD mortality based on different follow‐up duration in pooled analysis studies.
**Figure S27** Forest plot of the association between self‐reported PA with cancer mortality based on different follow‐up duration in pooled analysis studies.
**Figure S28** P‐curve analysis for self‐reported leisure‐time physical activity (LTPA).
**Figure S29** P‐curve analysis for Self‐reported total physical activity (TPA).
**Figure S30** P‐curve analysis for device‐measured total physical activity (TPA).
**Figure S31** P‐curve analysis for self‐reported sedentary behavior (SB).
**Figure S32** P‐curve analysis for device‐measured sedentary behavior (SB).
**Figure S33** P‐curve analysis for self‐reported walking time.
**Figure S34** P‐curve analysis for self‐reported running time.
**Figure S35** P‐curve analysis for self‐reported TV‐viewing.
**Figure S36** P‐curve analysis for pooled studies.
**Figure S37** P‐curve analysis for device‐measured Cardiorespiratory fitness.
**Figure S38** Influence analysis for self‐reported LTPA and all‐cause mortality.
**Figure S39** Influence analysis for device‐measured TPA and all‐cause mortality.
**Figure S40** Influence analysis for self‐reported SB and all‐cause mortality.
**Figure S41** Influence analysis for device‐measured SB and all‐cause mortality.
**Figure S42** Influence analysis for PA and all‐cause mortality in pooled analysis studies.
**Figure S43** Influence analysis for PA and CVD mortality in pooled analysis studies.
**Figure S44** Influence analysis for PA and cancer mortality in pooled analysis studies.
**Figure S45** Influence analysis for resistance training and all‐cause mortality.
**Figure S46** Influence analysis for resistance training and cancer mortality.
**Figure S47** Influence analysis for self‐reported TPA and all‐cause mortality.
**Figure S48** Influence analysis for self‐reported SB and CVD mortality.
**Figure S49** Influence analysis for self‐reported SB and cancer mortality.
**Figure S50** Influence analysis for self‐reported walking time and all‐cause mortality.
**Figure S51** Baujat plot for self‐reported LTPA and all‐cause mortality.
**Figure S52** Baujat plot for device‐measured TPA and all‐cause mortality.
**Figure S53** Baujat plot for self‐reported SB and all‐cause mortality.
**Figure S54** Baujat plot for device‐measured SB and all‐cause mortality.
**Figure S55** Baujat plot for PA and all‐cause mortality in pooled analysis studies.
**Figure S56** Baujat plot for PA and CVD mortality in pooled analysis studies.
**Figure S57** Baujat plot for PA and cancer mortality in pooled analysis studies.
**Figure S58** Baujat plot for resistance training and all‐cause mortality.
**Figure S59** Baujat plot for resistance training and cancer mortality.
**Figure S60** Baujat plot for self‐reported TPA and all‐cause mortality.
**Figure S61** Baujat plot for self‐reported SB and CVD mortality.
**Figure S62** Baujat plot for self‐reported SB and cancer mortality.
**Figure S63** Baujat plot for self‐reported walking time and all‐cause mortality.
