# Supplementary material for: Associations Between Exercise Training, Physical Activity, Sedentary Behaviour and Mortality: An Umbrella Review of Meta‐Analyses
Source: J Cachexia Sarcopenia Muscle. 2025 Mar 5;16(2):e13772. doi: 10.1002/jcsm.13772 (PMC11880915; doi:10.1002/jcsm.13772)
Supplement: Supplementary file 1 — Table S1 PRIOR checklist. Table S2 PRISMA 2020 abstract checklist adapted for umbrella reviews. Table S3 Studies excluded, with reason for exclusion. Table S4 Quality assessment and publication bias evaluation of included study using AMSTAR 2. Table S5 Finding across meta‐analyses of observational studies on health outcomes of physical activity and sedentary behavior. Table S6 Finding across individual meta‐analyses of included observational studies. Figure S1 Meta‐regression analysis for the association between physical activity and all‐cause mortality based on the moderating role of age. Figure S2 Meta‐regression analysis for the association between physical activity and CVD mortality based on the moderating role of age. Figure S3 Meta‐regression analysis for the association between physical activity and cancer mortality based on the moderating role of age. Figure S4 Forest plot of the association between different level of LTPA and all‐cause mortality. Figure S5 Forest plot of the association between different level of LTPA and CVD mortality. Figure S6 Forest plot of the association between different level of LTPA and cancer mortality. Figure S7 Forest plot of the association between different level of device‐measured TPA and cancer mortality. Figure S8 Forest plot of the association between different level of self‐reported TPA and cancer mortality. Figure S9 Forest plot of the association between different level of PA and all‐cause mortality in pooled analysis studies. Figure S10 Forest plot of the association between different level of PA and CVD mortality in pooled analysis studies. Figure S11 Forest plot of the association between different level of PA and cancer mortality in pooled analysis studies. Figure S12 Forest plot of the association between different measurement methods and all‐cause mortality in pooled analysis studies. Figure S13 Meta‐regression analysis for the association between total physical activity and cancer mortality based on the moderatin [file JCSM-16-e13772-s001.docx]

**Associations between exercise training, physical activity, sedentary behavior and all-cause, cardiovascular disease, and cancer mortality: An umbrella review of meta-analyses**

Masoud Rahmati ^1,2,3,4¶^*, Hyeri Lee ^5¶^, Hayeon Lee ^5^, Jaeyu Park ^5^, Djandan Tadum Arthur Vithran^¶^ ^6^, Yusheng Li ^6^, Abdolreza Kazemi^4^, Laurent Boyer ^1,2^, Guillaume Fond ^1,2^, Lee Smith ^7^, Nicola Veronese^8^, Pinar Soysal ^9^, Elena Dragioti ^10,11^, Samuele Cortese ^12,13,14,15,16^, Jiseung Kang ^17,18,19^, Dong Keon Yon ^5,20^*, Marco Solmi ^21,22,23,24^

^1^ CEReSS-Health Service Research and Quality of Life Center, Assistance Publique-Hopitaux de Marseille, Aix-Marseille University, Marseille, France. (MR: masoud.rahmati@univ-amu.fr; LB: laurent.boyer@ap-hm.fr; GF: [guillaume.fond@gmail.com](mailto:guillaume.fond@gmail.com)).

^2^ CRSMP, Center for Mental Health and Psychiatry Research – PACA, Marseille, France.

^3^ Department of Physical Education and Sport Sciences, Faculty of Literature and Human Sciences, Lorestan University, Khoramabad, Iran.

^4^ Department of Physical Education and Sport Sciences, Faculty of Literature and Humanities, Vali-E-Asr University of Rafsanjan, Rafsanjan, Iran. (RK: [rkazemi22@yahoo.com](mailto:rkazemi22@yahoo.com))

^5^ Center for Digital Health, Medical Science Research Institute, Kyung Hee University Medical Center, Kyung Hee University College of Medicine, Seoul, Republic of Korea, (Hyeri Lee: [hyerry97@naver.com](mailto:hyerry97@naver.com), Hayeon Lee: [wwhy28@khu.ac.kr](mailto:wwhy28@khu.ac.kr), JP: wodb980@naver.com),

^6^ National Clinical Research Center for Geriatric Disorders, Xiangya Hospital, Central South University, Changsha, China (YL: [liyusheng@csu.edu.cn](mailto:liyusheng@csu.edu.cn))

^7^ Centre for Health, Performance and Wellbeing, Anglia Ruskin University, Cambridge, UK ([lee.smith@aru.ac.uk](mailto:lee.smith@aru.ac.uk))

^8^ Saint Camillus International University of Health Sciences, Rome, Italy. ([nicola.veronese@unipa.it](mailto:nicola.veronese@unipa.it))

^9^ Department of Geriatric Medicine, Faculty of Medicine, Bezmialem Vakif University, Istanbul, Turkey ([dr.pinarsoysal@hotmail.com](mailto:dr.pinarsoysal@hotmail.com))

^10^ Pain and Rehabilitation Centre, and Department of Medical and Health Sciences, Linköping University, Linköping, Sweden

^11^ Research Laboratory Psychology of Patients, Families & Health Professionals, Department of Nursing, School of Health Sciences, University of Ioannina, Ioannina, Greece ([dragioti@uoi.gr](mailto:dragioti@uoi.gr))

^12^ Centre for Innovation in Mental Health, School of Psychology, Faculty of Environmental and Life Sciences, University of Southampton, Southampton, UK

^13^ Clinical and Experimental Sciences (CNS and Psychiatry), Faculty of Medicine, University of Southampton, Southampton, UK

^14^ Solent NHS Trust, Southampton, UK

^15^ Hassenfeld Children’s Hospital at NYU Langone, New York University Child Study Center, New York City, New York, USA

^16^ DiMePRe-J-Department of Precision and Regenerative Medicine-Jonic Area, University of Bari "Aldo Moro", Bari, Italy ([samuele.cortese@gmail.com](mailto:samuele.cortese@gmail.com))

^17^ Department of Anesthesia, Critical Care and Pain Medicine, Massachusetts General Hospital, Boston, MA, USA ([wltmd1006@gmail.com](file:///C:\Users\alber\Downloads\wltmd1006@gmail.com))

^18^ Division of Sleep Medicine, Harvard Medical School, Boston, Massachusetts, USA

^19^ School of Health and Environmental Science, College of Health Science, Korea University, Seoul, South Korea

^20^ Department of Pediatrics, Kyung Hee University College of Medicine, Seoul, Republic of Korea, ([yonkkang@gmail.com](mailto:yonkkang@gmail.com)).

^21^ On Track: The Champlain First Episode Psychosis Program, Department of Mental Health, The Ottawa Hospital, Ontario, Canada

^22^ Clinical Epidemiology Program, Ottawa Hospital Research Institute (OHRI), University of Ottawa, Ontario, Canada

^23^ Department of Child and Adolescent Psychiatry, Charité Universitätsmedizin, Berlin, Germany

^24^ Department of Psychiatry, University of Ottawa, Ottawa, ON, Canada (marco.solmi83@gmail.com)

^¶^ Masoud Rahmati, Hyeri Lee, and Djandan Tadum Arthur Vithran are joint first authors.

*** Corresponding authors:**

Masoud Rahmati, Department of Physical Education and Sport Sciences, Faculty of Literature and Human Sciences, Lorestan University, Khoramabad 6816785468, Iran; masoud.rahmati@univ-amu.fr

Dong Keon Yon, MD, PhD, FACAAI, FAAAAI, ATSF, Department of Pediatrics, Kyung Hee University College of Medicine, 23 Kyungheedae-ro, Dongdaemun-gu, Seoul, 02447, South Korea; yonkkang@gmail.com

**Supplementary Materials**

**Contents**

[**eMethods** 6](#_Toc187414476)

[**Supplementary table 1.** PRIOR checklist 7](#_Toc187414477)

[**Supplementary table 2.** PRISMA 2020 abstract checklist adapted for umbrella reviews 11](#_Toc187414478)

[**Supplementary table 3.** Studies excluded, with reason for exclusion. 12](#_Toc187414479)

[**Supplementary table 4.** Quality assessment and publication bias evaluation of included study using AMSTAR 2 16](#_Toc187414480)

[**Supplementary table 5.** Finding across meta-analyses of observational studies on health outcomes of physical activity and sedentary behavior. 19](#_Toc187414481)

[**Supplementary table 6.** Finding across individual meta-analyses of included observational studies. 26](#_Toc187414482)

[**Supplementary references** 32](#_Toc187414483)

[**Figure 1.** Meta-regression analysis for the association between physical activity and all-cause mortality based on the moderating role of age. 39](#_Toc187414484)

[**Figure 2.** Meta-regression analysis for the association between physical activity and CVD mortality based on the moderating role of age. 40](#_Toc187414485)

[**Figure 3.** Meta-regression analysis for the association between physical activity and cancer mortality based on the moderating role of age. 41](#_Toc187414486)

[**Figure 4.** Forest plot of the association between different level of LTPA and all-cause mortality. 42](#_Toc187414487)

[**Figure 5.** Forest plot of the association between different level of LTPA and CVD mortality. 43](#_Toc187414488)

[**Figure 6.** Forest plot of the association between different level of LTPA and cancer mortality. 44](#_Toc187414489)

[**Figure 7.** Forest plot of the association between different level of device-measured TPA and cancer mortality. 45](#_Toc187414490)

[**Figure 8.** Forest plot of the association between different level of self-reported TPA and cancer mortality. 46](#_Toc187414491)

[**Figure 9.** Forest plot of the association between different level of PA and all-cause mortality in pooled analysis studies. 47](#_Toc187414492)

[**Figure 10.** Forest plot of the association between different level of PA and CVD mortality in pooled analysis studies. 48](#_Toc187414493)

[**Figure 11.** Forest plot of the association between different level of PA and cancer mortality in pooled analysis studies. 49](#_Toc187414494)

[**Figure 12.** Forest plot of the association between different measurement methods and all-cause mortality in pooled analysis studies. 50](#_Toc187414495)

[**Figure 13.** Meta-regression analysis for the association between total physical activity and cancer mortality based on the moderating role of measurement methods. 51](#_Toc187414496)

[**Figure 14.** Forest plot of the association between different exercise training and all-cause mortality. 52](#_Toc187414497)

[**Figure 15.** Forest plot of the association between different exercise training and CVD mortality. 53](#_Toc187414498)

[**Figure 16.** Forest plot of the association between different exercise training and cancer mortality. 54](#_Toc187414499)

[**Figure 17.** Forest plot of the association between self-reported SB with CVD mortality based on different follow-up duration. 55](#_Toc187414500)

[**Figure 18.** Forest plot of the association between self-reported LTPA with all-cause mortality based on different follow-up duration. 56](#_Toc187414501)

[**Figure 19.** Forest plot of the association between self-reported LTPA with CVD mortality based on different follow-up duration. 57](#_Toc187414502)

[**Figure 20.** Forest plot of the association between self-reported LTPA with cancer mortality based on different follow-up duration. 58](#_Toc187414503)

[**Figure 21.** Forest plot of the association between self-reported TPA with all-cause mortality based on different follow-up duration. 59](#_Toc187414504)

[**Figure 22.** Forest plot of the association between self-reported SB with all-cause mortality based on different follow-up duration. 60](#_Toc187414505)

[**Figure 23.** Forest plot of the association between self-reported SB with CVD mortality based on different follow-up duration. 61](#_Toc187414506)

[**Figure 24.** Forest plot of the association between self-reported SB with cancer mortality based on different follow-up duration. 62](#_Toc187414507)

[**Figure 25.** Forest plot of the association between self-reported PA with all-cause mortality based on different follow-up duration in pooled analysis studies. 63](#_Toc187414508)

[**Figure 26.** Forest plot of the association between self-reported PA with CVD mortality based on different follow-up duration in pooled analysis studies. 64](#_Toc187414509)

[**Figure 27.** Forest plot of the association between self-reported PA with cancer mortality based on different follow-up duration in pooled analysis studies. 65](#_Toc187414510)

[**Figure 28.** P-curve analysis for self-reported leisure-time physical activity (LTPA). 66](#_Toc187414511)

[**Figure 29.** P-curve analysis for Self-reported total physical activity (TPA). 69](#_Toc187414512)

[**Figure 30.** P-curve analysis for device-measured total physical activity (TPA). 70](#_Toc187414513)

[**Figure 31.** P-curve analysis for self-reported sedentary behavior (SB). 71](#_Toc187414514)

[**Figure 32.** P-curve analysis for device-measured sedentary behavior (SB). 72](#_Toc187414515)

[**Figure 33.** P-curve analysis for self-reported walking time. 73](#_Toc187414516)

[**Figure 34.** P-curve analysis for self-reported running time. 74](#_Toc187414517)

[**Figure 35.** P-curve analysis for self-reported TV-viewing. 76](#_Toc187414518)

[**Figure 36.** P-curve analysis for pooled studies. 78](#_Toc187414519)

[**Figure 37.** P-curve analysis for device-measured Cardiorespiratory fitness. 81](#_Toc187414520)

[**Figure 38.** Influence analysis for self-reported LTPA and all-cause mortality. 84](#_Toc187414521)

[**Figure 39.** Influence analysis for device-measured TPA and all-cause mortality. 87](#_Toc187414522)

[**Figure 40.** Influence analysis for self-reported SB and all-cause mortality. 89](#_Toc187414523)

[**Figure 41.** Influence analysis for device-measured SB and all-cause mortality. **오류! 책갈피가 정의되어 있지 않습니다.**](#_Toc187414524)

[**Figure 42.** Influence analysis for PA and all-cause mortality in pooled analysis studies. 96](#_Toc187414525)

[**Figure 42.** Influence analysis for PA and CVD mortality in pooled analysis studies. 99](#_Toc187414526)

[**Figure 44.** Influence analysis for PA and cancer mortality in pooled analysis studies. 102](#_Toc187414527)

[**Figure 45.** Influence analysis for resistance training and all-cause mortality. 105](#_Toc187414528)

[**Figure 46.** Influence analysis for resistance training and cancer mortality. 108](#_Toc187414529)

[**Figure 47.** Influence analysis for self-reported TPA and all-cause mortality. 111](#_Toc187414530)

[**Figure 48.** Influence analysis for self-reported SB and CVD mortality. 114](#_Toc187414531)

[**Figure 49.** Influence analysis for self-reported SB and cancer mortality. 117](#_Toc187414532)

[**Figure 50.** Influence analysis for self-reported walking time and all-cause mortality. 120](#_Toc187414533)

[**Figure 51.** Baujat plot for self-reported LTPA and all-cause mortality. 123](#_Toc187414534)

[**Figure 52.** Baujat plot for device-measured TPA and all-cause mortality.](#_Toc187414535) [124](#_Toc187414536)

[**Figure 53.** Baujat plot for self-reported SB and all-cause mortality. 125](#_Toc187414537)

[**Figure 54.** Baujat plot for device-measured SB and all-cause mortality. 126](#_Toc187414538)

[**Figure 55.** Baujat plot for PA and all-cause mortality in pooled analysis studies. 127](#_Toc187414539)

[**Figure 56.** Baujat plot for PA and CVD mortality in pooled analysis studies. 128](#_Toc187414540)

[**Figure 57.** Baujat plot for PA and cancer mortality in pooled analysis studies. 129](#_Toc187414541)

[**Figure 58.** Baujat plot for resistance training and all-cause mortality. 130](#_Toc187414542)

[**Figure 59.** Baujat plot for resistance training and cancer mortality. 131](#_Toc187414543)

[**Figure 60.** Baujat plot for self-reported TPA and all-cause mortality. 132](#_Toc187414544)

[**Figure 61.** Baujat plot for self-reported SB and CVD mortality. 133](#_Toc187414545)

[**Figure 62.** Baujat plot for self-reported SB and cancer mortality. 134](#_Toc187414546)

[**Figure 63.** Baujat plot for self-reported walking time and all-cause mortality. 135](#_Toc187414547)

# **eMethods**

**Search key**

The medical subject heading terms and keywords were used in the search include (“physical activity” OR "sedentary behavior" OR "sitting time" OR " TV-viewing time" OR "cardiorespiratory fitness" OR "daily steps" OR "all-cause mortality" OR " cardiovascular disease mortality" OR "cancer mortality") AND (“meta-analysis” OR “systematic review”), using truncated terms for all fields.

# **Supplementary table 1.** PRIOR checklist

| **Section Topic** | **#** | **Item** | **Location reported** |
| --- | --- | --- | --- |
| **TITLE** |  |  |  |
| Title | 1 | Identify the report as an overview of reviews. | Pg 1 |
| **ABSTRACT** |  |  |  |
| Abstract | 2 | Provide a comprehensive and accurate summary of the purpose, methods, and results of the overview of reviews. | Pg 4-5 |
| **INTRODUCTION** |  |  |  |
| Rationale | 3 | Describe the rationale for conducting the overview of reviews in the context of existing knowledge. | Pg 6 |
| Objectives | 4 | Provide an explicit statement of the objective(s) or question(s) addressed by the overview of reviews. | Pg 7 |
| **METHODS** |  |  |  |
| Eligibility criteria | 5a | Specify the inclusion and exclusion criteria for the overview of reviews. If supplemental primary studies were included, this should be stated, with a rationale. | Pg 8 |
|  | 5b | Specify the definition of ‘systematic review’ as used in the inclusion criteria for the overview of reviews. | Pg 8 |
| Information sources | 6 | Specify all databases, registers, websites, organizations, reference lists, and other sources searched or consulted to identify systematic reviews and supplemental primary studies (if included).  Specify the date when each source was last searched or consulted. | Pg 8 |
| Search strategy | 7 | Present the full search strategies for all databases, registers and websites, such that they could be reproduced. Describe any search filters and limits applied. | Pg 8 |
| Selection process | 8a | Describe the methods used to decide whether a systematic review or supplemental primary study (if included) met the inclusion criteria of the overview of reviews. | Pg 8 |
|  | 8b | Describe how overlap in the populations, interventions, comparators, and/or outcomes of systematic reviews was identified and managed during study selection. | Pg 8 |
| Data collection process | 9a | Describe the methods used to collect data from reports. | Pg 8 |
|  | 9b | If applicable, describe the methods used to identify and manage primary study overlap at the level of the comparison and outcome during data collection. For each outcome, specify the method used to illustrate and/or quantify the degree of primary study overlap across systematic reviews. | Pg 8 |
|  | 9c | If applicable, specify the methods used to manage discrepant data across systematic reviews during data collection. | Pg 8 |
| Data items | 10 | List and define all variables and outcomes for which data were sought. Describe any assumptions made and/or measures taken to identify and clarify missing or unclear information. | Pg 8 |
| Risk of bias assessment | 11a | Describe the methods used to assess risk of bias or methodological quality of the included systematic reviews. | Pg 8 |
|  | 11b | Describe the methods used to collect data on (from the systematic reviews) and/or assess the risk of bias of the primary studies included in the systematic reviews. Provide a justification for instances where flawed, incomplete, or missing assessments are identified but not re-assessed. | Pg 8 |
|  | 11c | Describe the methods used to assess the risk of bias of supplemental primary studies (if included). | Pg 8 |
| Synthesis methods | 12a | Describe the methods used to summarize or synthesize results and provide a rationale for the choice(s). | Pg 8-9 |
|  | 12b | Describe any methods used to explore possible causes of heterogeneity among results. | Pg 9 |
|  | 12c | Describe any sensitivity analyses conducted to assess the robustness of the synthesized results. | Pg 9 |
| Reporting bias assessment | 13 | Describe the methods used to collect data on (from the systematic reviews) and/or assess the risk of bias due to missing results in a summary or synthesis (arising from reporting biases at the levels of the systematic reviews, primary studies, and supplemental primary studies, if included). | Pg 8-9 |
| Certainty assessment | 14 | Describe the methods used to collect data on (from the systematic reviews) and/or assess certainty (or confidence) in the body of evidence for an outcome. | Pg 9 |
| **RESULTS** |  |  |  |
| Systematic review and supplemental primary study selection | 15a | Describe the results of the search and selection process, including the number of records screened, assessed for eligibility, and included in the overview of reviews, ideally with a flow diagram. | Pg 10 |
|  | 15b | Provide a list of studies that might appear to meet the inclusion criteria, but were excluded, with the main reason for exclusion. | Appendix |
| Characteristics of systematic reviews and supplemental primary studies | 16 | Cite each included systematic review and supplemental primary study (if included) and present its characteristics. | Pg 10-11 |
| Primary study overlap | 17 | Describe the extent of primary study overlap across the included systematic reviews. | Pg 10-11 |
| Risk of bias in systematic reviews, primary studies, and supplemental primary studies | 18a | Present assessments of risk of bias or methodological quality for each included systematic review. | Pg 10-11 |
|  | 18b | Present assessments (collected from systematic reviews or assessed anew) of the risk of bias of the primary studies included in the systematic reviews. | Pg 10-11 |
|  | 18c | Present assessments of the risk of bias of supplemental primary studies (if included). | Pg 10-11 |
| Summary or synthesis of results | 19a | For all outcomes, summarize the evidence from the systematic reviews and supplemental primary studies (if included). If meta-analyses were done, present for each the summary estimate and its precision and measures of statistical heterogeneity. If comparing groups, describe the direction of the effect. | Pg 10-11 |
|  | 19b | If meta-analyses were done, present results of all investigations of possible causes of heterogeneity. | Pg 10-11 |
|  | 19c | If meta-analyses were done, present results of all sensitivity analyses conducted to assess the robustness of synthesized result | Pg 10-11 |
| Reporting biases | 20 | Present assessments (collected from systematic reviews and/or assessed a new) of the risk of bias due to missing primary studies, analyses, or results in a summary or synthesis (arising from reporting biases at the levels of the systematic reviews, primary studies, and supplemental primary studies, if included) for each summary or synthesis assessed. | Pg 10-11 |
| Certainty of evidence | 21 | Present assessments (collected or assessed anew) of certainty (or confidence) in the body of evidence for each outcome. | Pg 10-11 |
| **DISCUSSION** |  |  |  |
| Discussion | 22a | Summarize the main findings, including any discrepancies in findings across the included systematic reviews and supplemental primary studies (if included). | Pg 12 |
|  | 22b | Provide a general interpretation of the results in the context of other evidence. | Pg 12-13 |
|  | 22c | Discuss any limitations of the evidence from systematic reviews, their primary studies, and supplemental primary studies (if included) included in the overview of reviews. Discuss any limitations of the overview of reviews methods used. | Pg 13-14 |
|  | 22d | Discuss implications for practice, policy, and future research (both systematic reviews and primary research). Consider the relevance of the findings to the end users of the overview of reviews, e.g., healthcare providers, policymakers, patients, among others. | Pg 13 |
| **OTHER INFORMATION** | | |  |
| Registration and protocol | 23a | Provide registration information for the overview of reviews, including register name and registration number, or state that the overview of reviews was not registered. | Pg 8 |
|  | 23b | Indicate where the overview of reviews protocol can be accessed, or state that a protocol was not prepared. | Pg 8 |
|  | 23c | Describe and explain any amendments to information provided at registration or in the protocol. Indicate the stage of the overview of reviews at which amendments were made. | Pg 8 |
| Support | 24 | Describe sources of financial or non-financial support for the overview of reviews, and the role of the funders or sponsors in the overview of reviews. | Pg 15 |
| Competing interests | 25 | Declare any competing interests of the overview of reviews' authors. | Pg 15 |
| Author information | 26a | Provide contact information for the corresponding author. | Pg 1 |
|  | 26b | Describe the contributions of individual authors and identify the guarantor of the overview of reviews. | Pg 15 |
| Availability of data and other materials | 27 | Report which of the following are available, where they can be found, and under which conditions they may be accessed: template data collection forms; data collected from included systematic reviews and supplemental primary studies; analytic code; any other materials used in the overview of reviews. | Pg 15 |

# **Supplementary table 2.** PRISMA 2020 abstract checklist adapted for umbrella reviews

| **Section Topic** | **#** | **Item** | **Reported?** |
| --- | --- | --- | --- |
| **Title** |  |  |  |
| Title | **1** | Identify the report as a UR. | Y |
| **Background** |  |  |  |
| Objectives | **2** | Provide an explicit statement of the main objective(s) or question(s) the UR addresses. | Y |
| **Methods** |  |  |  |
| Eligibility criteria | **3** | Specify the inclusion and exclusion criteria for the UR. | Y |
| Information sources | **4** | Specify the information sources (e.g. databases, registers) used to identify studies and the date when each was last searched. | Y |
| Quality | **5** | Specify the methods used to assess quality in the included reviews/meta-analyses. | Y |
| Synthesis of results | **6** | Specify the methods used to present and synthesise results, including exploring biases. | Y |
| **Results** |  |  |  |
| Included studies | **7** | Give the total number of included comparisons, reviews, studies, and describe reviews’ quality | Y |
| Synthesis of results | **8** | Present results for main outcomes or of most credible evidence, preferably indicating the number of included studies and participants for each. If meta-analysis was done, report the summary estimate and confidence/credible interval. If comparing groups, indicate the direction of the effect (i.e. which group is favoured). | Y |
| **Discussion** |  |  |  |
| Limitations of evidence | **9** | Provide a brief summary of the limitations of the evidence included in the UR (e.g. review quality, inconsistency and imprecision). | Y |
| Interpretation | **10** | Provide a general interpretation of the results and important implications. | Y |
| **Other** |  |  |  |
| Funding | **11** | Specify the primary source of funding for the UR. | Y |
| Registration | **12** | Provide the register name and registration number. | Y |

# **Supplementary table 3.** Studies excluded, with reason for exclusion.

| **Author, year** | **Reason for exclusion** |
| --- | --- |
| Abioye, 2015^1^ | No outcome of interest |
| Al-Zalabani, 2016^2^ | No outcome of interest |
| Aune, 2017^3^ | No outcome of interest |
| Aune, 2021^4^ | No outcome of interest |
| Bahls, 2021^5^ | Mendelian randomization study |
| Ballard-Barbash, 2012^6^ | No meta-analysis |
| Bao, 2008^7^ | No outcome of interest |
| Barone, 2016^8^ | No meta-analysis |
| Barry, 2014^9^ | No outcome of interest |
| Batty, 2022^10^ | No meta-analysis |
| Bauman, 2004^11^ | No meta-analysis |
| Behrens, 2014^12^ | No outcome of interest |
| Behrens, 2015^13^ | No outcome of interest |
| Belvederi Murri, 2020^14^ | No meta-analysis |
| Bennett, 2015^15^ | No outcome of interest |
| Berlin, 1990^16^ | No outcome of interest |
| Berzigotti, 2016^17^ | No meta-analysis |
| Blair, 1999^18^ | No meta-analysis |
| Buffart, 2014^19^ | No outcome of interest |
| Canabrava, 2019^20^ | No meta-analysis |
| CHA, 2023^21^ | No outcome of interest |
| Cust, 2007^22^ | No meta-analysis |
| Chen, 2014^23^ | No outcome of interest |
| Colpani, 2018^24^ | No outcome of interest |
| Cooper, 2010^25^ | No outcome of interest |
| Cunningham, 2020^26^ | No outcome of interest |
| de Souto Barreto, 2019^27^ | No outcome of interest |
| Desnoyers, 2016^28^ | No meta-analysis |
| Diep, 2010^29^ | No outcome of interest |
| Dhote, 2000^30^ | No meta-analysis |
| Eaton, 1992^31^ | No outcome of interest |
| Ekelund, 2018^32^ | No meta-analysis |
| Ekelund, 2023^33^ | No meta-analysis |
| Erlichman, 2002^34^ | No meta-analysis |
| Farris, 2015^35^ | No outcome of interest |
| Friedenreich, 2011^36^ | No meta-analysis |
| Ferrario, 2018^37^ | No outcome of interest |
| Fogelholm, 2010^38^ | No outcome of interest |
| Ford, 2012^39^ | No meta-analysis |
| Gallanagh, 2011^40^ | No meta-analysis |
| Garcia-Hermoso, 2020^41^ | No outcome of interest |
| Geidl, 2020^42^ | No outcome of interest |
| Gonçalves, 2014^43^ | No meta-analysis |
| Gonzalez-Jaramillo, 2022^44^ | No meta-analysis |
| Hackshaw, 2015^45^ | No meta-analysis |
| Hall, 2020^46^ | No meta-analysis |
| Harriss, 2009^47^ | No outcome of interest |
| Hershey, 2022^48^ | No meta-analysis |
| Katzmarzyk, 2003^49^ | No meta-analysis |
| Keum, 2014^50^ | Dose-response meta-analysis |
| Khan, 2015^51^ | No outcome of interest |
| Kokkinos, 2012^52^ | No meta-analysis |
| Kraus, 2019^53^ | No meta-analysis |
| Kyu, 2016^54^ | No outcome of interest |
| Ku, 2018^55^ | Dose-response meta-analysis |
| Je, 2013^56^ | No outcome of interest |
| Jiménez-Pavón, 2019^57^ | No outcome of interest |
| Jarczok, 2022^58^ | No outcome of interest |
| Lacombe, 2019^59^ | No outcome of interest |
| Lahart, 2015^60^ | No outcome of interest |
| Lavie, 2015^61^ | No meta-analysis |
| Lagerros, 2004^62^ | No meta-analysis |
| Lemez, 2015^63^ | No meta-analysis |
| Lee, 2001^64^ | No meta-analysis |
| Lee, 2003^65^ | No meta-analysis |
| Lee, 2017^66^ | No meta-analysis |
| Lee, 2020^67^ | No outcome of interest |
| Leitzmann, 2015^68^ | No meta-analysis |
| Liang, 2022^69^ | No outcome of interest |
| Li, 2012^70^ | No outcome of interest |
| Li, 2013^71^ | No outcome of interest |
| Li, 2016^72^ | No outcome of interest |
| Liu, 2011^73^ | No outcome of interest |
| Liu, 2016^74^ | No outcome of interest |
| Loef, 2012^75^ | No outcome of interest |
| Lopez, 2013^76^ | No meta-analysis |
| Lopez, 2020^77^ | No outcome of interest |
| Lynch, 2010^78^ | No meta-analysis |
| Lynch, 2017^79^ | No meta-analysis |
| MacKinnon, 2018^80^ | No outcome of interest |
| Maisonneuve, 2015^81^ | No meta-analysis |
| Martins, 2021^82^ | No meta-analysis |
| McTiernan, 2019^83^ | No meta-analysis |
| Meneses-Echávez, 2015^84^ | No outcome of interest |
| Michaud, 2001^85^ | No meta-analysis |
| Milton, 2014^86^ | No meta-analysis |
| Monninkhof, 2007^87^ | No meta-analysis |
| Moore, 2010^88^ | No meta-analysis |
| Naci, 2013^89^ | No outcome of interest |
| Nicolson, 2021^90^ | No outcome of interest |
| Noguchi, 2015^91^ | No outcome of interest |
| Oguma, 2001^92^ | No meta-analysis |
| Oliveira, 2023^93^ | No outcome of interest |
| Olsen, 2007^94^ | No outcome of interest |
| Oguma, 2004^95^ | No outcome of interest |
| O'Rorke, 2010^96^ | No outcome of interest |
| Pham, 2012^97^ | No meta-analysis |
| Patterson, 2018^98^ | Dose-response meta-analysis |
| Pandey, 2015^99^ | No outcome of interest |
| Perreault, 2017^100^ | No outcome of interest |
| Prince, 2021^101^ | No meta-analysis |
| Ponticelli, 2021^102^ | No meta-analysis |
| Pozuelo-Carrascosa, 2021^103^ | No outcome of interest |
| Proper, 2011^104^ | No meta-analysis |
| Rahmati, 2022^105^ | No outcome of interest |
| Ramsey, 2021^106^ | No meta-analysis |
| Reimers, 2012^107^ | No meta-analysis |
| Ristow, 2022^108^ | No outcome of interest |
| Robsahm, 2013^109^ | No outcome of interest |
| Rocha, 2022^110^ | No outcome of interest |
| Rossi, 2012^111^ | No outcome of interest |
| Samad, 2015^112^ | No outcome of interest |
| Sattelmair, 2011^113^ | No outcome of interest |
| Sergentanis, 2015^114^ | No outcome of interest |
| Schmid, 2014^115^ | No outcome of interest |
| Schmid, 2016^116^ | No outcome of interest |
| Schnohr, 2009^117^ | No meta-analysis |
| Singh, 2014^118^ | No outcome of interest |
| Sittichai, 2022^119^ | No outcome of interest |
| Sofi, 2008^120^ | No outcome of interest |
| Spence, 2009^121^ | No meta-analysis |
| Sun, 2012^122^ | No outcome of interest |
| Tajabadi, 2019^123^ | No meta-analysis |
| Tardon, 2005^124^ | No outcome of interest |
| Taylor, 2020^125^ | No meta-analysis |
| Teramoto, 2010^126^ | No meta-analysis |
| Thorp, 2011^127^ | No meta-analysis |
| Tolley, 2021^128^ | No meta-analysis |
| Van Uffelen, 2010^129^ | No meta-analysis |
| Vermaete, 2013^130^ | No outcome of interest |
| Volaklis, 2015^131^ | No meta-analysis |
| Voskuil, 2007^132^ | No meta-analysis |
| Wahid, 2016^133^ | No outcome of interest |
| Wendel-Vos, 2004^134^ | No outcome of interest |
| Wang, 2022^135^ | No outcome of interest |
| Warburton, 2010^136^ | No meta-analysis |
| Warburton, 2017^137^ | No meta-analysis |
| Wu, 2023^138^ | Dose-response meta-analysis |
| Vogel, 2009^139^ | No meta-analysis |
| Xie, 2022^140^ | No outcome of interest |
| Xu, 2019^141^ | Dose-response meta-analysis |
| Yang, 2022^142^ | No meta-analysis |
| Yerrakalva, 2015^143^ | No meta-analysis |
| Zelenović, 2021^144^ | No meta-analysis |
| Zelenović, 2022^145^ | No meta-analysis |
| Zhang, 2013^146^ | No outcome of interest |
| Zhang, 2016^147^ | No outcome of interest |
| Zhang, 2022^148^ | No meta-analysis |
| Zhao, 2019^149^ | Dose-response meta-analysis |
| Zheng, 2015^150^ | No meta-analysis |
| Zhong, 2013^151^ | No outcome of interest |
| Zhong, 2016^152^ | No outcome of interest |
| Zhou, 2014^153^ | No outcome of interest |
| Zhuo, 2021^154^ | Mendelian randomization study |

# **Supplementary table 4.** Quality assessment and publication bias evaluation of included study using AMSTAR 2

| **Author** | **Q1** | **Q2** | **Q3** | **Q4** | **Q5** | **Q6** | **Q7** | **Q8** | **Q9** | **Q10** | **Q11** | **Q12** | **Q13** | **Q14** | **Q15** | **Q16** | **All** |
| --- | --- | --- | --- | --- | --- | --- | --- | --- | --- | --- | --- | --- | --- | --- | --- | --- | --- |
| Arem, 2015* | Yes | No | Yes | No | No | No | No | No | Yes | No | No | No | No | Yes | Yes | No | Critically low |
| Banach, 2023 | Yes | No | Yes | Yes | Yes | Yes | Yes | Yes | Yes | No | Yes | Yes | Yes | Yes | No | No | Moderate |
| Biswas, 2015 | Yes | No | Yes | Yes | No | Yes | Yes | Yes | Yes | No | Yes | Yes | Yes | Yes | Yes | No | High |
| Blond, 2020 | Yes | Yes | Yes | Yes | Yes | Yes | Yes | Yes | Yes | Yes | Yes | Yes | Yes | Yes | Yes | Partial Yes | High |
| Chastin, 2019 | Yes | No | Yes | Yes | Yes | Yes | No | Yes | Yes | Yes | Yes | Yes | Yes | Yes | Yes | No | Moderate |
| Chastin, 2021* | No | Yes | Yes | Yes | Yes | Yes | Yes | Yes | No | Yes | Yes | No | No | Yes | No | Partial Yes | Moderate |
| Chau, 2013 | Yes | No | Yes | Yes | Yes | Yes | Yes | Yes | Yes | Yes | Yes | Yes | No | Yes | Yes | Partial Yes | Moderate |
| Cheng, 2018 | Yes | No | Yes | Yes | Yes | Yes | No | No | No | No | Yes | No | No | Yes | Yes | No | Critically low |
| Ekelund, 2016 | No | No | No | Yes | Yes | Yes | Yes | Yes | Yes | No | Yes | No | No | Yes | No | No | Critically low |
| Ekelund, 2019 (1) | Yes | Yes | Yes | Yes | Yes | Yes | Yes | Yes | Yes | No | Yes | Yes | Yes | Yes | No | No | High |
| Ekelund, 2019 (2) | Yes | No | Yes | Yes | No | No | No | Yes | No | No | No | Yes | Yes | Yes | No | Partial Yes | Critically low |
| Garcia, 2023 | Yes | Yes | Yes | Yes | Yes | Yes | Yes | Yes | Yes | Yes | Yes | Yes | Yes | Yes | Yes | Partial Yes | High |
| Grøntved, 2011 | Yes | Yes | Yes | Yes | Yes | Yes | Yes | Yes | Yes | Yes | Yes | No | No | Yes | Yes | Partial Yes | High |
| Hamer, 2008 | Yes | No | Yes | Yes | No | No | No | Yes | Yes | Yes | Yes | Yes | Yes | Yes | Yes | Partial Yes | Moderate |
| Han, 2022 | Yes | No | Yes | Yes | Yes | Yes | Yes | Yes | Yes | Yes | Yes | Yes | No | No | Yes | Partial Yes | Low |
| Hupin, 2015 | Yes | No | Yes | Yes | Yes | Yes | No | No | No | No | Yes | No | No | Yes | No | No | Critically low |
| Jayedi,2021 | Yes | No | Yes | Yes | Yes | Yes | Yes | Yes | Yes | No | Yes | Yes | Yes | Yes | Yes | No | High |
| Kelly, 2014 | Yes | Yes | Yes | Yes | Yes | Yes | Yes | Yes | Yes | No | Yes | Yes | Yes | Yes | Yes | No | High |
| Kodama, 2009 | Yes | No | Yes | Yes | Yes | Yes | Yes | Yes | Yes | Yes | Yes | Yes | Yes | Yes | Yes | Partial Yes | High |
| Ku, 2019 | Yes | No | Yes | Yes | Yes | Yes | Yes | Yes | Yes | Yes | Yes | Yes | Yes | No | Yes | Partial Yes | High |
| Liew, 2023 | Yes | No | Yes | Yes | Yes | Yes | Yes | Yes | Yes | No | Yes | Yes | Yes | Yes | No | No | Moderate |
| Liu, 2018* | No | No | Yes | No | No | NO | No | Yes | Yes | Yes | Yes | No | Yes | No | No | Partial Yes | Critically low |
| Löllgen, 2009 | Yes | No | Yes | Yes | Yes | Yes | No | No | Yes | No | Yes | Yes | Yes | Yes | Yes | No | Low |
| Merom, 2016* | No | No | No | No | No | No | No | No | No | No | No | No | No | No | No | No | Critically low |
| Milton, 2008 | Yes | No | Yes | Yes | Yes | Yes | Yes | Yes | Yes | Yes | Yes | Yes | Yes | Yes | Yes | Partial Yes | High |
| Momma, 2022 | Yes | Yes | Yes | Yes | Yes | Yes | No | Yes | Yes | Yes | Yes | Yes | Yes | Yes | Yes | Partial Yes | High |
| Moore, 2012* | No | Yes | No | No | No | No | No | No | No | Yes | No | No | No | No | No | Partial Yes | Critically low |
| Nascimento, 2021 | Yes | Yes | Yes | Yes | Yes | Yes | No | No | Yes | Yes | Yes | Yes | Yes | Yes | No | No | Moderate |
| Nocon, 2008 | Yes | No | No | Yes | No | No | No | Yes | Yes | No | No | No | No | Yes | Yes | No | Critically low |
| O’Donovan, 2017* | No | Yes | Yes | Yes | Yes | Yes | Yes | Yes | Yes | Yes | Yes | Yes | Yes | Yes | Yes | Partial Yes | High |
| Paluch, 2022 | Yes | Yes | Yes | Yes | Yes | Yes | No | Yes | Yes | Yes | Yes | Yes | Yes | Yes | Yes | Partial Yes | High |
| Pedisic, 2019 | Yes | Yes | Yes | Yes | Yes | Yes | Yes | Yes | Yes | Yes | Yes | Yes | Yes | Yes | No | Partial Yes | High |
| Qiu, 2021 | Yes | Yes | Yes | Yes | No | No | Yes | Yes | Yes | Yes | Yes | Yes | Yes | Yes | Yes | No | High |
| Ramakrishnan, 2021 | Yes | Yes | Yes | No | Yes | Yes | No | Yes | Yes | Yes | Yes | Yes | Yes | Yes | Yes | Partial Yes | Moderate |
| Rojer, 2020 | Yes | Yes | Yes | Yes | Yes | Yes | Yes | Yes | Yes | Yes | Yes | Yes | Yes | Yes | Yes | Partial Yes | High |
| Runacres, 2021 | Yes | Yes | No | Yes | Yes | Yes | Yes | Yes | Yes | Yes | Yes | Yes | Yes | Yes | Yes | Partial Yes | High |
| Saeidifard, 2019 | Yes | No | No | Yes | Yes | Yes | Yes | Yes | Yes | Yes | Yes | Yes | Yes | Yes | No | Partial Yes | Moderate |
| Samitz, 2011 | Yes | No | No | Yes | Yes | Yes | Yes | Yes | Yes | Yes | Yes | Yes | Yes | Yes | Yes | Partial Yes | High |
| Schmid, 2015 | Yes | No | No | Yes | Yes | Yes | Yes | Yes | Yes | No | Yes | Yes | Yes | Yes | Yes | No | High |
| Shailendra, 2022 | Yes | Yes | Yes | Yes | Yes | Yes | Yes | Yes | Yes | No | Yes | Yes | Yes | Yes | Yes | No | High |
| Sheng, 2021 | Yes | Yes | Yes | Yes | Yes | Yes | No | Yes | Yes | Yes | Yes | Yes | Yes | Yes | Yes | Partial Yes | High |
| Stamatakis, 2018 | Yes | No | No | No | No | No | No | Yes | Yes | Yes | No | No | No | No | No | Partial Yes | Critically low |
| Stens,2023 | Yes | Yes | Yes | Yes | Yes | Yes | Yes | Yes | Yes | Yes | Yes | Yes | Yes | Yes | Yes | Partial Yes | High |
| Sun, 2015 | Yes | No | Yes | Yes | Yes | Yes | Yes | Yes | Yes | Yes | Yes | Yes | Yes | No | Yes | Partial Yes | High |
| Takagi, 2019 | Yes | No | Yes | Yes | No | No | Yes | Yes | Yes | No | Yes | Yes | No | No | Yes | No | Moderate |
| Wang, 2023 | Yes | Yes | Yes | Yes | Yes | Yes | No | Yes | Yes | Yes | Yes | Yes | Yes | Yes | Yes | Partial Yes | High |
| Wilmot, 2012 | Yes | Yes | Yes | Yes | Yes | Yes | Yes | Yes | Yes | Yes | Yes | Yes | Yes | Yes | Yes | Partial Yes | High |
| Woodcock, 2011 | Yes | No | Yes | Yes | Yes | Yes | No | Yes | No | Yes | Yes | Yes | Yes | Yes | Yes | Partial Yes | Critically low |
| Zhou, 2023 | Yes | Yes | Yes | Yes | Yes | Yes | Yes | Yes | Yes | Yes | Yes | Yes | Yes | Yes | Yes | Partial Yes | High |

AMSTAR, assess the methodological quality of systematic reviews; Q, Question; Q1: Did the research questions and inclusion criteria for the review include the components of PICO?, Q2: Did the report of the review contain an explicit statement that the review methods were established prior to the conduct of the review and did the report justify any significant deviations from the protocol?, Q3: Did the review authors explain their selection of the study designs for inclusion in the review?, Q4: Did the review authors use a comprehensive literature search strategy?, Q5: Did the review authors perform study selection in duplicate?, Q6: Did the review authors perform data extraction in duplicate?, Q7: Did the review authors provide a list of excluded studies and justify the exclusions?, Q8: Did the review authors describe the included studies in adequate detail?, Q9: Did the review authors use a satisfactory technique for assessing the risk of bias (RoB) in individual studies that were included in the review?, Q10: Did the review authors report on the sources of funding for the studies included in the review?, Q11: If meta-analysis was performed, did the review authors use appropriate methods for statistical combination of results?, Q12: If meta-analysis was performed, did the review authors assess the potential impact of RoB in individual studies on the results of the meta-analysis or other evidence synthesis?, Q13: Did the review authors account for RoB in primary studies when interpreting/discussing the results of the review?, Q14: Did the review authors provide a satisfactory explanation for, and discussion of, any heterogeneity observed in the results of the review?, Q15: If they performed quantitative synthesis did the review authors carry out an adequate investigation of publication bias (small study bias) and discuss its likely impact on the results of the review?, Q16: Did the review authors report any potential sources of conflict of interest, including any funding they received for conducting the review?

# **Supplementary table 5.** Finding across meta-analyses of observational studies on health outcomes of physical activity and sedentary behavior.

| **Outcome** | **Included studies (or cohorts)** | **Metric** | **Re-analyzed summary ES (95% CI)** | | | | **Re-analyzed summary P value** | **Heterogeneity I^2^ (%)** | | **Tau^2^** | **Egger’s p-value** | **Small study effect** | **95% Prediction interval** | **Credibility of evidence** |
| --- | --- | --- | --- | --- | --- | --- | --- | --- | --- | --- | --- | --- | --- | --- |
|  |  |  | **Fix effect model** | **Random effect model** | **Largest study** | **HS method; random-effect model** |  |  |  |  |  |  |  |  |
| **1. Self-reported leisure-time physical activity (LTPA)** | | | | | | | | | | | | | | |
| All-cause mortality | 7 | HR | 0.73 (0.71 to 0.75) | 0.73 (0.68 to 0.78) | 0.77 (0.73 to 0.80) | 0.73 (0.66 to 0.80) | 0.20x10^-3^ | 79.63 | | 4.86x10^-3^ | 0.941 | N | (0.59, 0.91) | Suggestive |
| Cardiovascular disease mortality | 7 | HR | 0.75 (0.73 to 0.77) | 0.74 (0.70 to 0.78) | 0.77 (0.74 to 0.81) | 0.74 (0.69 to 0.80) | <.0001 | 76.85 | | 4.10x10^-3^ | 0.403 | N | (0.61, 0.89) | Suggestive |
| Cardiovascular disease mortality (SA) | 5 | HR | 0.75 (0.73, 0.78) | 0.73 (0.67, 0.80) | 0.81 (0.77, 0.85) | 0.73 (0.65, 0.83) | 0.002 | 83.2 | | 0.008 | 0.52 | N | (0.51, 1.06) | Suggestive |
| Cancer mortality | 6 | HR | 0.88 (0.86 to 0.90) | 0.87 (0.83 to 0.91) | 0.90 (0.88 to 0.93) | 0.87 (0.83 to 0.91) | 0.60 x10^-3^ | 55.51 | | 1.32x10^-3^ | 0.719 | N | (0.77, 0.98) | Suggestive |
| **2. Self-reported total physical activity (TPA)** | | | | | | | | | | | | | | |
| All-cause mortality | 7 | HR | 0.82 (0.80 to 0.84) | 0.78 (0.69 to 0.87) | 0.88 (0.85 to 0.91) | 0.78 (0.70 to 0.86) | 0.80 x10^-3^ | 91.17 | | 1.95 | 0.298 | N | (0.02, 28.23) | Suggestive |
| All-cause mortality (SA) | 2 | HR | 0.88 (0.85, 0.91) | 0.88 (0.85, 0.91) | 0.88 (0.85, 0.91) | 0.88 (0.77, 1.00) | 0.049 | 0 | | <0.001 | - | N | - | Week |
| **3. Device-measured total physical activity (TPA)** | | | | | | | | | | | | | | |
| All-cause mortality | 9 | HR | 0.50 (0.47 to 0.54) | 0.50 (0.40 to 0.63) | 0.71 (0.62 to 0.73) | 0.50 (0.38 to 0.65) | 0.30 x10^-3^ | 95.79 | | 0.12 | 0.418 | N | (0.17, 1.29) | Suggestive |
| All-cause mortality (SA) | 7 | HR | 0.57 (0.53, 0.61) | 0.52 (0.42, 0.64) | 0.71 (0.62, 0.82) | 0.52 (0.40, 0.67) | 0.001 | 86.7 | | 0.0675 | 0.0582 | N | (0.24, 1.10) | Suggestive |
| **4. Self-reported sedentary behavior (SB)** | | | | | | | | | | | | | | |
| All-cause mortality | 4 | HR | 1.30 (1.26 to 1.34) | 1.30 (1.25 to 1.36) | 1.27 (1.22 to 1.32) | 1.3 (1.22 to 1.38) | 0.10 x10^-2^ | 33.02 | | 5.78 x10^-4^ | 0.787 | NA | (1.09, 1.55) | Weak |
| All-cause mortality (SA) | 3 | HR | 1.33 (1.28, 1.39) | 1.33 (1.28, 1.39) | 1.34 (1.28, 1.40) | 1.33 (1.22, 1.45) | 0.005 | 0 | | <0.001 | 0.96 | N | (0.76, 2.35) | Suggestive |
| Cardiovascular disease mortality | 4 | HR | 1.37 (1.31 to 1.44) | 1.47 (1.19 to 1.80) | 1.18 (1.09 to 1.26) | 1.47 (1.04 to 2.07) | 0.39 x10^-1^ | 94.05 | | 3.85 x10^-2^ | 0.596 | NA | (0.47, 4.57) | Weak |
| Cardiovascular disease mortality (SA) | 3 | HR | 1.25 (1.18, 1.32) | 1.32 (1.14, 1.53) | 1.18 (1.09, 1.26) | 1.32 (0.83, 2.12) | 0.126 | 80.60 | | 0.01 | 0.32 | N | (0.05, 37.74) | NA |
| Cancer mortality | 3 | HR | 1.20 (1.15 to 1.24) | 1.20 (1.15 to 1.24) | 1.21 (1.14 to 1.28) | 1.20 (1.13 to 1.26) | 0.49 x10^-2^ | 54 | | <0.001 | 0.690 | N | (0.84, 1.71) | Weak |
| Cancer mortality (SA) | 2 | HR | 1.19 (1.14, 1.24) | 1.19 (1.14, 1.24) | 1.21 (1.14, 1.28) | 1.19 (0.96, 1.47) | 0.061 | 0 | | <0.001 | - | N | - | NA |
| **5. Device-measured sedentary behavior (SB)** | | | | | | | | | | | | | | |
| All-cause mortality | 3 | HR | 2.14 (1.81 to 2.53) | 2.16 (1.57 to 2.96) | 1.58 (1.19 to 2.09) | 2.16 (1.09 to 4.28) | 0.40 x10^-1^ | 71.50 | | 5.55 x10^-2^ | 0.333 | NA | (0.01, 457.13) | Weak |
| All-cause mortality (SA) | 2 | HR | 2.53 (2.05, 3.12) | 2.53 (2.05, 3.12) | 2.44 (1.82, 3.25) | 2.53 (1.57, 4.07) | 0.026 | 0 | | <0.001 | - | N | - | Weak |
| **6. Self-reported walking time** | | | | | | | | | | | | | | |
| All-cause mortality | 4 | HR | 0.86 (0.82 to 0.90) | 0.79 (0.69 to 0.90) | 0.89 (0.83 to 0.96) | 0.79 (0.61 to 1.00) | 0.05 | 86.41 | | 1.63 x10^-2^ | 0.083 | N | (0.05, 12.79) | Weak |
| All-cause mortality (SA) | 2 | HR | 0.84 (0.79, 0.90) | 0.78 (0.60, 1.02) | 0.89 (0.83, 0.96) | 0.78 (0.14, 4.32) | 0.320 | 91.09 | | 0.03 | - | N | - | NA |
| **7. Self-reported running time** | | | | | | | | | | | | | | |
| All-cause mortality | 2 | HR | 0.82 (0.79 to 0.84) | 0.78 (0.69 to 0.89) | 0.83 (0.80 to 0.85) | 0.78 (0.35 to 1.76) | 0.16 | 89.67 | | 7.39 x10^-3^ | - | N | - | NA |
| Cardiovascular disease mortality | 2 | HR | 0.81 (0.77 to 0.85) | 0.81 (0.77 to 0.85) | 0.81 (0.77 to 0.85) | 0.81 (0.62 to 1.05) | 0.61 x10^-1^ | <0.001 | | <0.001 | - | N | - | NA |
| Cancer mortality | 2 | HR | 0.88 (0.85 to 0.91) | 0.84 (0.73 to 0.96) | 0.89 (0.86 to 0.92) | 0.84 (0.34 to 2.07) | 0.25 | 79.75 | | 8.36 x10^-3^ | - | N | - | NA |
| **8. Device-measured daily steps** | | | | | | | | | | | | | | |
| All-cause mortality | 7 | HR | 0.33 (0.25 to 0.43) | 0.45 (0.37 to 0.53) | 0.39 (0.32 to 0.48) | 0.44 (0.35 to 0.56) | 0.2 x10^-3^ | 84.98 | | 4.32 x10^-2^ | - | N | (0.25, 0.80) | Suggestive |
| **8. Device-measured daily steps per 1000 steps increment** | | | | | | | | | | | | | | |
| All-cause mortality | 2 | HR | 0.85 (0.81 to 0.91) | 0.86 (0.83 to 0.90) | 0.88 (0.83 to 0.93) | 0.86 (0.69 to 1.07) | 0.07 | <0.001 | | <0.001 | - | N | - | NA |
| **9. Self-reported TV-viewing** | | | | | | | | | | | | | | |
| All-cause mortality | 2 | HR | 1.21 (1.16 to 1.26) | 1.27 (1.00 to 1.61) | 1.13 (1.07 to 1.18) | 1.27 (0.27 to 5.94) | 0.30 | 96.38 | | 2.83 x10^-2^ | - | NA | - | NA |
| Cardiovascular disease mortality | 3 | HR | 1.67 (1.53 to 1.82) | 1.68 (1.25 to 2.25) | 1.59 (1.38 to 1.80) | 1.68 (0.85 to 3.30) | 0.81 x10^-1^ | 91.01 | | 6.04 x10^-2^ | 0.904 | Y | (0.01, 365.35) | NA |
| Cancer mortality | 2 | HR | 1.27 (1.15 to 1.41) | 1.27 (1.15 to 1.41) | 1.26 (1.10 to 1.45) | 1.27 (1.10 to 1.48) | 0.31 x10^-1^ | <0.001 | | <0.001 | - | NA | - | Weak |
| **10. Pooled studies** | | | | | | | | | | | | | | |
| All-cause mortality | 14 | HR | 0.69 (0.68 to 0.70) | 0.70 (0.66 to 0.74) | 0.68 (0.66 to 0.69) | 0.70 (0.64 to 0.76) | <.0001 | 96.26 | | 1.23 x10^-2^ | 0.594 | Y | (0.54, 0.91) | Suggestive |
| All-cause mortality (SA) | 5 | HR | 0.66 (0.63, 0.69) | 0.64 (0.58, 0.70) | 0.69 (0.65, 0.74) | 0.64 (0.55, 0.74) | 0.001 | 66.70 | | 0.01 | 0.20 | N | (0.44, 0.92) | Suggestive |
| Cardiovascular disease mortality | 11 | HR | 0.66 (0.65 to 0.68) | 0.69 (0.62 to 0.76) | 0.67 (0.65 to 0.70) | 0.69 (0.61, 0.78) | <.0001 | 94.04 | | 2.32 x10-^2^ | 0.491 | Y | (0.47, 1.00) | Suggestive |
| Cardiovascular disease mortality (SA) | 3 | HR | 0.62 (0.55, 0.68) | 0.62 (0.55, 0.68) | 0.63 (0.55, 0.72) | 0.62 (0.56, 0.67) | 0.002 | 0 | | <0.001 | 0.41 | N | (0.34, 1.10) | Suggestive |
| Cancer mortality | 9 | HR | 0.83 (0.82 to 0.85) | 0.84 (0.78 to 0.90) | 0.92 (0.88 to 0.95) | 0.84 (0.78 to 0.90) | 0.00060 | 93.10 | | 1.12 x10^-2^ | 0.861 | N | (0.64, 1.09) | Suggestive |
| Cancer mortality (SA) | 3 | HR | 0.84 (0.77, 0.91) | 0.84 (0.77, 0.91) | 0.86 (0.77, 0.96) | 0.84 (0.75, 0.94) | 0.021 | 0 | | <0.001 | 0.49 | N | (0.41, 1.73) | Weak |
| **11. Resistance training** | | | | | | | | | | | | | | |
| All-cause mortality | 4 | HR | 0.82 (0.77 to 0.86) | 0.82 (0.77 to 0.86) | 0.85 (0.79 to 0.93) | 0.82 (0.76 to 0.88) | 0.33 x10^-2^ | <0.001 | | <0.001 | 0.241 | NA | (0.69, 0.96) | Weak |
| All-cause mortality (SA) | 3 | HR | 0.83 (0.78, 0.88) | 0.83 (0.78, 0.88) | 0.85 (0.79, 0.93) | 0.83 (0.76, 0.91) | 0.012 | 0 | | <0.001 | 0.19 | N | (0.47, 1.48) | Weak |
| Cardiovascular disease mortality | 3 | HR | 0.82 (0.77 to 0.89) | 0.82 (0.77 to 0.89) | 0.82 (0.74 to 0.91) | 0.82 (0.81 to 0.84) | 0.50 x10^-3^ | <0.001 | | <0.001 | 0.591 | N | (0.73, 0.93) | Suggestive |
| Cancer mortality | 5 | HR | 0.84 (0.79 to 0.89) | 0.84 (0.79 to 0.89) | 0.88 (0.80 to 0.97) | 0.84 (0.77 to 0.92) | 0.52 x10^-2^ | 4.58 | | 2.54x10^-4^ | 0.231 | N | (0.72, 0.98) | Weak |
| Cancer mortality (SA) | 4 | HR | 0.85 (0.80, 0.91) | 0.85 (0.80, 0.91) | 0.88 (0.80, 0.97) | 0.85 (0.81, 0.90) | 0.002 | 0 | | <0.001 | 0.46 | N | (0.77, 0.95) | Suggestive |
| **12. Aerobic training** | | | | | | | | | | | | | | |
| All-cause mortality | 3 | HR | 0.73 (0.68 to 0.79) | 0.73 (0.66 to 0.80) | 0.75 (0.67 to 0.84) | 0.73 (0.57 to 0.93) | 0.30 x10^-1^ | 32.13 | | 2.51x10^-3^ | - | N | (0.13, 3.95) | Weak |
| **13. Aerobic plus Resistance training** | | | | | | | | | | | | | | |
| All-cause mortality | 4 | HR | 0.60 (0.54 to 0.67) | 0.60 (0.56 to 0.64) | 0.60 (0.54 to 0.66) | 0.60 (0.56 to 0.64) | <.0001 | <0.001 | | <0.001 | - | N | (0.38, 0.94) | Suggestive |
| Cardiovascular disease mortality | 3 | HR | 0.52 (0.44 to 0.62) | 0.52 (0.44 to 0.62) | 0.54 (0.41 to 0.70) | 0.52 (0.41 to 0.66) | 0.72 x10^-2^ | <0.001 | | <0.001 | - | NA | (0.11, 2.44) | Weak |
| Cancer mortality | 4 | HR | 0.80 (0.71 to 0.91) | 0.80 (0.71 to 0.91) | 0.90 (0.75 to 1.08) | 0.80 (0.65 to 0.99) | 0.42 x10^-1^ | <0.001 | | <0.001 | - | NA | (0.51, 1.26) | Weak |
| **14. Device-measured cardiorespiratory fitness** | | | | | | | | | | | | | | |
| All-cause mortality | 3 | HR | 0.61 (0.57 to 0.65) | 0.58 (0.49 to 0.69) | 0.67 (0.61 to 0.74) | 0.58 (0.38 to 0.89) | 0.32 x10^-1^ | 83.32 | | 1.84x10^-2^ | 0.284 | NA | (0.02, 15.43) | Weak |
| Cardiovascular disease mortality | 3 | HR | 0.52 (0.48 to 0.58) | 0.51 (0.43 to 0.61) | 0.49 (0.42 to 0.56) | 0.51 (0.34 to 0.77) | 0.19 x10^-1^ | 66.28 | | 1.61x10^-2^ | 0.622 | Y | (0.02, 10.95) | Weak |
| Cancer mortality | 4 | HR | 0.69 (0.65 to 0.75) | 0.67 (0.55 to 0.80) | 0.76 (0.69 to 0.84) | 0.67 (0.49 to 0.90) | 0.24 x10^-1^ | 82.17 | | 2.91x10^-2^ | 0.438 | N | (0.25, 1.80) | Weak |
| **15. Cardiorespiratory fitness per one-MET increase in CRF** | | | | | | | | | | | | | | |
| All-cause mortality | 3 | HR | 0.87 (0.85 to 0.90) | 0.87 (0.85 to 0.90) | 0.87 (0.84 to 0.90) | 0.87 (0.82 to 0.93) | 0.24 x10^-1^ | <0.001 | | <0.001 | - | NA | (0.57, 1.32) | Weak |
| Cardiovascular disease mortality | 2 | HR | 0.86 (0.83 to 0.88) | 0.86 (0.83 to 0.88) | 0.85 (0.82 to 0.88) | 0.86 (0.74 to 0.99) | 0.46 x10^-1^ | <0.001 | | <0.001 | - | NA | - | Weak |
| **16. Cardiorespiratory incidence** | | | | | | | | | | | | | | |
| Incidence | 3 | HR | 0.64 (0.60, 0.68) | 0.56 (0.42, 0.73) | 0.67 (0.63, 0.72) | 0.55 (0.31, 1.00) | 0.05 | 86.23 | 0.05 | | 0.32 | N | (0.00, 66.37) | Weak |
| **17. Low skeletal muscle mass** | | | | | | | | | | | | | | |
| All-cause mortality | 2 | HR | 1.37 (1.30, 1.45) | 1.40 (1.25, 1.57) | 1.36 (1.28, 1.44) | 1.40 (0.67, 2.92) | 0.062 | 31.76 | 0.003 | | - | N | - | NA |

Legend. HR, hazard ratio; N, no; NA, not assessable; Y, yes; SA, sensitivity analysis after removing studies with low and critically low qualities.

# **Supplementary table 6.** Finding across individual meta-analyses of included observational studies.

| **Study** | **Sample size** | | **Exposure** | **Comparison** | **Outcome** | **Effect size** | | **I^2^** | **Significance** | | | **Biases** | | **Quality** |
| --- | --- | --- | --- | --- | --- | --- | --- | --- | --- | --- | --- | --- | --- | --- |
|  | **k** | **Participants** |  |  |  | **Metric** | **ES (95% CI)** |  | **p** | **PI sign** | **LS sign** | **SSE** | **ESB** |  |
| Arem, 2015* | 6 | 661 137 | Self-reported TPA | High vs. inactive  Moderate vs. inactive population  Low vs. inactive  High vs. inactive population  Moderate vs. inactive population  Low vs. inactive  High vs. inactive population  Moderate vs. inactive population  Low vs. inactive population | All-cause mortality  CVD mortality  Cancer mortality | HR | 0.72 (0.68-0.76)  0.71 (0.68-0.73)  0.73 (0.71-0.75)  0.58 (0.56-0.61)  0.59 (0.57-0.63)  0.67 (0.65-0.70)  0.74 (0.71-0.77)  0.75 (0.72-0.79)  0.79 (0.75-0.82) | NA | NR | Y | Y | Y | Y | Critically low |
| Banach, 2023 | 17 | 226889 | Device‐measured daily steps | High vs. very low  Moderate vs. very low  Low vs. very low  Per 1000 steps increment  High vs. very low  Moderate vs. very low  Low vs. very low  Per 500 steps increment | All-cause mortality  CVD mortality | HR | 0.33 (0.25-0.43)  0.45 (0.33-0.60)  0.51 (0.47-0.56)  0.85 (0.81-0.91)  0.33 (0.28-0.44)  0.51 (0.42-0.62)  0.84 (0.73-0.97)  0.93 (0.91-0.95) | 33  11 | <0.001  =.001  0.001  <0.00001  <0.001  <0.001  0.021  <0.00001 | N | N | N | N | Moderate |
| Biswas, 2015Y | 41 | 2126809 | Self-reported SB | High vs. low ST | All-cause mortality  CVD mortality  Cancer mortality | HR | 1.24 (1.09-1.41)  1.18 (1.11–1.24)  1.16 (1.10–1.22) | 94.96  19.22  0.23 | <0.001  0.170  0.54 | N | N | N | N | High |
| Blond, 2020 | 48 | 2038756 | Self-reported running time  Device‐measured running time  Self-reported TPA  Self-reported LTPA | Higher levels of PA vs. the recommended level (30 vs. 7.5 MET h/ week) | All-cause mortality  CVD mortality  Cancer mortality  All-cause mortality  CVD mortality  Cancer mortality  All-cause mortality  CVD mortality  Cancer mortality  All-cause mortality  CVD mortality  Cancer mortality | HR | 0.83 (0.80-0.85)  0.81 (0.77-0.85)  0.89 (0.86-0.92)  0.47 (0.34-0.65)  0.80 (0.71-0.90)  0.93 (0.79-1.11)  0.88 (0.85-0.91)  0.76 (0.66-0.88)  0.88 (0.78-0.98)  0.90 (0.77-1.05)  0.82 (0.68-1.00)  0.93 (0.79-1.11) | 77–80  68–74  7–22 | <0.001 | Y | Y | Y | N | High |
| Chastin, 2019 | 8 | 48513 | Device‐measured TPA | Light PA vs. inactive population | All-cause mortality | HR | 0.71 (0.62-0.73) | 73 | <0.00001 | N | N | N | N | Moderate |
| Chastin, 2021* | 6 | 130 239 | Device‐measured TPA | MVPA vs. inactive population  Light PA vs. inactive population | All-cause mortality | HR | 0.63 (0.55-0.71)  0.5 (0.42-0.62) | 92.2 | <0.001 | N | N | N | N | Moderate |
| Chau, 2013 | 6 | 595,086 | Self-reported/ device‐measured SB | High vs. low SB | All-cause mortality | HR | 1.34 (1.28-1.40) | 82.7 | <0.001 | N | N | Y | Y | Moderate |
| Cheng, 2018 | 44 | 1,584,181 | Self-reported LTPA | High vs. low-intensity LTPA  Moderate vs. low-intensity LTPA | CVD mortality | HR | 0.73 (0.69–0.77)  0.77 (0.74–0.81) | NR | 0.119  0.106 | Y | N | N | N | Critically low |
| Cillekens, 2022 | 23 | 655 892 | Self-reported OPA | High vs. low OPA  Moderate OPA vs. Low OPA  Moderate OPA vs. inactive population | CVD mortality | HR | 0.99 (0.90–1.09)  0.85 (0.77–0.94)  1.03 (0.92–1.15) | 71  39  79 | 0.14  NR  NR | N | N | N | N | Critically low |
| Coenen, 2018 | 17 | 193 696 | Self-reported OPA | High vs. low OPA | All-cause mortality | HR | 1.18 (1.05-1.34) | 76 | <0.00001 | N | N |  |  | High |
| Ekelund, 2016 | 16 | 1 005 791 | Self-reported SB  Self-reported TV-viewing time | High vs. low SB  High vs. low TV-viewing time  High vs. low SB  High vs. low TV-viewing time  High vs. low SB  High vs. low TV-viewing time | All-cause mortality  CVD mortality  Cancer mortality | HR | 1·27 (1·22–1·32)  1·44 (1·34–1·56)  1.74 (1.60-1.90)  2.26 (1.93-2.66)  1.22 (1.13-1.31)  1.26 (1.10-1.45) | NR | NR | Y | Y | Y | Y | Critically low |
| Ekelund, 2019 (1) | 8 | 850 060 | Device‐measured TPA  Device‐measured SB | High vs. low TPA  High vs. moderate to vigorous TPA  High vs. low SB | All-cause mortality | HR | 0.27 (0.23-0.32)  0.52 (0.43-0.61)  2.63 (1.94- 3.56) | NR | NR | N | N | N | N | High |
| Ekelund, 2019  (2) | 9 | 36383 | Self-reported SB  Self-reported TV-viewing time | High vs. low SB  High vs. low TV-viewing time  High vs. low SB  High vs. low TV-viewing time | CVD mortality  Cancer mortality | HR | 1.32 (0.21-1.43)  1.59 (1.38-1.80)  1.21 (1.14-1.28)  1.29 (1.10-1.51) | NR | NR | Y | Y | Y | Y | High |
| Garcia, 2023 | 191 | 163,415,543 | Self-reported LTPA | High vs. very low LTPA  Moderate vs. very low LTPA  Low vs. very low LTPA  High vs. very low LTPA  Moderate vs. very low LTPA  Low vs. very low LTPA  High vs. very low LTPA  Moderate vs. very low LTPA  Low vs. very low LTPA | All-cause mortality  CVD mortality  Cancer mortality | RR | 0.66 (0.62-0.70)  0.69 (0.65-0.73)  0.77 (0.73-0.80)  0.65 (0.60-0.71)  0.71 (0.66-0.77)  0.81 (0.77-0.85)  0.82 (0.77-0.86)  0.85 (0.81-0.89)  0.90 (0.88-0.93) | NR | NR | Y | N | Y | N | Moderate |
| Grøntved, 2011 | 8 | 175 938 | Self-reported TV viewing | TV-viewing time (2h/d) | All-cause mortality | RR | 1.13 (1.07-1.18) | 50.4 | 0.001 | N | N | N | N | Low |
| Hamer, 2008 | 11 | 147063 | Self-reported walking time | Highest walking vs. lowest walking | All-cause mortality | HR | 0.68 (0.59-0.78) | 31.35 | 0.001 | N | N | N | N | Moderate |
| Han, 2022 | 34 | 625 400 | Device‐measured CRF | Per one-MET increase in CRF  High vs. low CRF  Moderate vs. low CRF | All-cause mortality  CVD mortality  Cancer mortality  All-cause mortality  CVD mortality  Cancer mortality  All-cause mortality  CVD mortality  Cancer mortality | RR | 0.88 (0.83-0.93)  0.87 (0.83- 0.91)  0.93 (0.91-0.96)  0.47 (0.39–0.56)  0.49 (0.42–0.56)  0.57 (0.46–0.70)  0.67 (0.61–0.74)  0.60 (0.51–0.69)  0.76 (0.69–0.84) | 99.4  80.3  76.6 | <0.001  <0.001  <0.001 | N | N | N | N | Low |
| Hupin, 2015 | 9 | 122 417 | Self-reported TPA | High vs. very low TPA  Moderate vs. very low TPA  Low vs. very low TPA | All-cause mortality | RR | 0.65 (0.61-0.70)  0.72 (0.65-0.80)  0.78 (0.71-0.87) | NR | <0.0001 | N | N | N | N | Critically low |
| Jayedi, 2021 | 7 | 175370 | Device‐measured daily steps | Per 1000 steps increment | All-cause mortality | HR | 0.88 (0.83-0.93) | 79 | <0.0001 | N | N | N | N | High |
| Kelly, 2014 | 18 | 280000 | Self-reported walking time Self-reported cycling time | International PA recommendations (11.25 MET.hours per week) | All-cause mortality  All-cause mortality | RR | 0.89 (0.83-0.96)  0.90 (0.87-0.94) | 82.6  20.5 | <0.05  0.27 | N | N | N | N | High |
| Kodama, 2009 | 33 | 102 980 | Device‐measured CRF | Per one-MET increase in CRF | All-cause mortality  CVD mortality | RR | 0.87 (0.84-0.90)  0.85 (0.82-0.88) | 82.3  74.7 | 0.001 | N | N | N | N | High |
| Ku, 2019 | 11 | 49 239 | Device‐measured TPA | High vs. very low TPA Low vs. very Moderate vs. very low TPA  low TPA | All-cause mortality | HR | 0.56 (0.44‐0.71)  0.68 (0.59‐0.79)  0.71 (0.62‐0.82) | 35.11 | 0.019 | N | N | N | N | High |
| Liew, 2023 | 20 | 13,606 | Device‐measured TPA  Device‐measured SB | High vs. low TPA  High vs. moderate-to-vigorous TPA  High vs. very low TPA  High vs. low SB  High vs. low TPA  High vs. moderate-to-vigorous TPA  High vs. vvery low TPA  High vs. low SB | All-cause mortality  CVD mortality | HR | 0.42 (0.34-0.53)  0.43 (0.35-0.53)  0.58 (0.43-0.80)  1.58 (1.19-2.09)  0.29 (0.18-0.47)  0.37 (0.25-0.55)  0.62 (0.41-0.93)  1.89 (1.09, 3.29) | 0.0  4.4  9.3  31.8  0.0  50.5  34.7  61.7 | 0.55  0.39  0.35  0.14  0.41  0.049  0.22  0.01 | N | N | N | N | Moderate |
| Liu, 2018* | 9 | 467 729 | Self-reported LTPA | High vs. very low LTPA  Moderate vs. very low LTPA  Low vs. very low LTPA  High vs. very low LTPA  Moderate vs. very low LTPA  Low vs. very low LTPA  High vs. very low LTPA  Moderate vs. very low LTPA  Low vs. very low LTPA | All-cause mortality  CVD mortality  Cancer mortality | HR | 0.86 (0.82-0.91)  0.86 (0.81-0.92)  0.85 (0.81-0.90)  0.84 (0.77-0.92)  0.86 (0.78-0.94)  0.83 (0.78-0.89)  0.93 (0.89-0.98)  0.94 (0.87-1.00)  0.92 (0.88-0.95) | NR | NR | Y | Y | Y | Y | Critically low |
| Löllgen, 2009 | 38 | 271 000 | Self-reported TPA | Highly active vs. inactive  Moderately active vs. inactive population | All-cause mortality | RR | 0.80 (0.66–0.97)  0.78 (0.61–1.00) | NR | NR | N | N | N | N | Low |
| Merom, 2016* | 11 | 48,390 | Self-reported walking time  Self-reported dancing time | Moderate walking vs. inactive  Low walking vs. inactive population  Moderate dancing vs. inactive population  Low dancing vs. inactive population | CVD mortality | HR | 0.47 (0.29-0.75)  0.86 (0.65-1.13)  0.35 (0.27-0.45)  0.81 (0.69-0.95) | NR | <0.001 | N | N | N | N | Critically low |
| Momma, 2022 | 16 | 581 194 | Self-reported Resistance training | Aerobic only  RT vs. inactive  RT plus AT vs. inactive population | All-cause mortality  CVD mortality  Cancer mortality | RR | 0.75 (0.67-0.84)  0.85 (0.79-0.93)  0.60 (0.54-0.67)  0.71 (0.61-0.81)  0.83 (0.73-.93)  0.54 (0.41-0.7)  0.89 (0.72-1.10)  0.88 (0.80-0.97)  0.72 (0.53-0.98) | 59.28  NR  NR  62.55  NR  NR  84.78  NR  NR | <0.086  NR  NR  0.069  NR  NR  0.001  NR  NR | Y | Y | Y | N | High |
| Moore, 2012* | 6 | 654,827 | Self-reported LTPA | High vs. inactive population  Moderate vs. inactive population  Low vs. inactive population | All-cause mortality | HR | 0.59 (0.57–0.61)  0.61 (0.59–0.63)  0.68 (0.66–0.69) | 95.8  92.6  64.6 | <0.01  <0.01  0.02 | N | N | N | N | Critically low |
| Nascimento, 2021 | 12 | 1,297,620 | Self-reported resistance training and LTPA | RT vs. inactive population  LTPA vs. inactive population  RT plus LTPA vs. inactive | Cancer mortality | HR | 0.83 (0.73-0.94)  0.89 (0.72-1.10)  0.72 (0.53-0.98) | 21  90  85 | 0.92  NR  NR | N | N | N | N | Moderate |
| Nocon, 2008 | 33 | 883 372 | Self-reported CRF  Device‐measured CRF | High vs. low CRF  High vs. low CRF  High vs. low CRF  High vs. low CF | All-cause mortality  CVD mortality | RR | 0·71 (0·66-0·76)  0·59 (0·53-0·65)  0·70 (0·66-0·74)  0·43 (0·33-0·57) | 80.1  19.0  40.3  60.3 | 0.00001  0.27  0.27  0.007 | N | N | N | N | Critically low |
| O’Donovan, 2017* | 11 | 63 591 | Self-reported LTPA | High vs. very low LTPA  Moderate vs. very low LTPA  Low vs. very low LTPA | All-cause mortality  CVD mortality  Cancer mortality | HR | 0.65 (0.58-0.73)  0.70 (0.60-0.82)  0.69 (0.65-0.74)  0.59 (0.48-0.73)  0.60 (0.45-0.82)  0.63 (0.55-0.72)  0.79 (0.66-0.94)  0.82 (0.63-1.06)  0.86 (0.77-0.96) | NR | NR | N | N | N | N | High |
| Paluch, 2022 | 15 | 47 471 | Device‐measured daily steps | High vs. very low  Moderate vs. very low  Low vs. very low | All-cause mortality | HR | 0·39 (0·32-0·48)  0·47 (0·40-0·56)  0·56 (0·47-0·65) | 47  12  52 | NR | N | N | N | N | High |
| Pedisic, 2019 | 6 | 232 149 | Self-reports running time | High vs. no running | All-cause mortality  CVD mortality  Cancer mortality | HR | 0.73 (0.68-0.79)  0.70 (0.49-0.98)  0.77 (0.68-0.87) | 8.54  63.44  NR | <0.001  0.040  NR | N | N | N | N | High |
| Qiu, 2021 | 25 | 170,000 | Self-reported CRF | Per 1-MET higher of CRF | All-cause mortality  CVD mortality | HR | 0.83 (0.78- 0.88)  0.83 (0.80-0.86) | 93.6  65.0 | < 0.05 | N | N | N | N | High |
| Ramakrishnan, 2021 | 15 | 141,582 | Device‐measured TPA | High vs. low TPA | All-cause mortality | HR | 0.33 (0.25-0.43) | 54.2 | 0.016 | N | N | N | N | Moderate |
| Rojer, 2020 | 12 | 38,141 | Device‐measured SB  Device‐measured daily steps | High vs. low SB  High vs. low | All-cause mortality | HR | 2.44 (1.82–3.25)  3.09 (2.33–4.11) | 70.3  69.2 | <0.001  0.04 | Y | Y | Y | Y | High |
| Runacres, 2021 | 44 | 165,000 | Elite athletes | General population | All-cause mortality  CVD mortality  Cancer mortality | HR | 0.67 (0.59-0.75)  0.73 (0.62-0.85)  0.75 (0.63-0.89) | 96.9  81.8  88.1 | <0.01  <0.05  <0.01 | N | N | N | N | High |
| Saeidifard, 2019 | 11 | 370,256 | Self-reported resistance training and LTPA | RT vs. inactive  LTPA vs. inactive population  RT plus LTPA vs. inactive  RT vs. inactive population  RT plus LTPA vs. inactive  RT vs. inactive population  RT plus LTPA vs. inactive | All-cause mortality  CVD mortality  Cancer mortality | HR | 0.79 (0.69–0.91)  0.59 (0.45–0.76)  0.60 (0.49–0.72)  0.83 (0.68–1.03)  0.43 (0.27–0.70)  0.81 (0.54–1.20)  0.90 (0.75–1.08) | 73  52  53  49  27  2 | 0.0009  0.07  0.07  0.0005  0.29  0.25 | N | N | N | N | Moderate |
| Samitz, 2011 | 33 | 1 338 143 | Self-reported TPA  Self-reported LTPA  Self-reported OPA | High vs. low TPA  High vs. low LTPA  High vs. low OPA | All-cause mortality | RR | 0.65(0.60-0.71)  0.74 (0.70–0.77)  0.83 (0.71–0.97) | 79.4  68.1  87.6 | <0.001  0.018  <0.001 | N | N | N | N | High |
| Schmid, 2015 | 6 | 71 654 | Device‐measured CRF | High vs. low CRF  Moderate vs. low CRF | Cancer mortality | RR | 0.80 (0.67–0.97  0.55 (0.47–0.65) | 38  0 | 0.17  0.43 | N | N | N | N | High |
| Shailendra, 2022 | 10 | NA | Self-reported resistance training and LTPA | RT vs. inactive population  LTPA vs. inactive population  RT plus LTPA vs. inactive  RT vs. inactive population  LTPA vs. inactive population  RT plus LTPA vs. inactive  RT vs. inactive population  LTPA vs. inactive population  RT plus LTPA vs. inactive population | All-cause mortality  CVD mortality  Cancer mortality | RR | 0.82 (0.72-0.93)  0.75 (0.67-0.84)  0.60 (0.54-0.66)  0.82 (0.74-0.91)  0.71 (0.61-0.81)  0.54 (0.41-0.70)  0.84 (0.75-0.94)  0.89 (0.72-1.10)  0.72 (0.53-0.98) | 73.4  88.0  57.5  0  65.2  61.0  11.1  90.3  84.8 | NR | N | N | N | N | High |
| Sheng, 2021 | 16 | 132,674 | Device‐measured daily steps | High vs. low | All-cause mortality  CVD mortality | RR | 0·31 (0·23-0·42)  0·41 (0·25-0·67) | 56.8  70.6 | 0.014  0.017 | N | N | N | N | High |
| Stamatakis, 2018 | 11 | 736,463 | Self-reported resistance training | RT vs. inactive population | All-cause mortality  Cancer mortality | HR | 0.77 (0.69-0.87)  0.69 (0.56-0.86) | NR | NR | N | N | N | N | Critically low |
| Stens, 2023 | 12 | 111309 | Device‐measured daily steps | High vs. low  Moderate vs. low | All-cause mortality | HR | 0.50 (0.42-0.60)  0.64 (0.56-0.72) | 53  62 | <0.01  <0.01 | Y | N | Y | Y | High |
| Sun, 2015 | 10 | 647,475 | Self-reported TV viewing | High vs. low TV-viewing time | All-cause mortality | HR | 1.33 (1.20- 1.47) | 66.7 | 0.001 | N | N | N | N | High |
| Takagi, 2019 | 15 | 924,971 | Self-reported TV viewing | High vs. low TV-viewing time | CVD mortality | HR | 1.32 (1.12-1.55) | 53 | 0.05 | N | N | N | N | Moderate |
| Wang, 2023 | 16 | 11696 | Muscle wasting | Low vs. normal muscle mass | All-cause mortality | HR | 1.57 (1.25- 1.96) | 94.9 | <0.001 | N | N | N | N | High |
| Wilmot, 2012 | 18 | 482,117 | Self-reported SB | High vs. low SB | All-cause mortality CVD mortality | RR | 1.49 (1.14-2.03)  1.90 (1.36-2.66) | NR | NR |  |  | Y | N | High |
| Woodcock, 2011 | 22 | 977 925 | Self-reported TPA  Self-reported walking time | 11 vs. 0 MET-h/week | All-cause mortality | RR | 0.81 (0.76-0.85)  0.89 (0.82-0.96) | 86  75 | <0.001  0.003 | N | N | N | N | Critically low |
| Zhou, 2023 | 49 | 878349 | Muscle wasting | Low vs. normal muscle mass | All-cause mortality  CVD mortality  Cancer mortality | HR | 1.36 (1.59-1.75)  1.29 (1.05-1.58)  1.75 (1.02-1.27) | 96.5 | <0.001 | N | N | N | N | High |

Legend. LS, largest study with significant effect; PI, prediction interval; ESB, excess significance bias; SSE, small study effects; sign., significant; Y, yes; N, no.

# **Supplementary references**

1 Abioye, A. I., Odesanya, M. O., Abioye, A. I. & Ibrahim, N. A. Physical activity and risk of gastric cancer: a meta-analysis of observational studies. *British journal of sports medicine* **49**, 224-229 (2015).

2 Al-Zalabani, A. H., Stewart, K. F., Wesselius, A., Schols, A. M. & Zeegers, M. P. Modifiable risk factors for the prevention of bladder cancer: a systematic review of meta-analyses. *European journal of epidemiology* **31**, 811-851 (2016).

3 Aune, D. *et al.* Resting heart rate and the risk of cardiovascular disease, total cancer, and all-cause mortality–a systematic review and dose–response meta-analysis of prospective studies. *Nutrition, Metabolism and Cardiovascular Diseases* **27**, 504-517 (2017).

4 Aune, D. *et al.* Physical activity and the risk of heart failure: a systematic review and dose–response meta-analysis of prospective studies. *European journal of epidemiology* **36**, 367-381 (2021).

5 Bahls, M. *et al.* Physical activity, sedentary behavior and risk of coronary artery disease, myocardial infarction and ischemic stroke: a two-sample Mendelian randomization study. *Clinical Research in Cardiology*, 1-10 (2021).

6 Ballard-Barbash, R. *et al.* Physical activity, biomarkers, and disease outcomes in cancer survivors: a systematic review. *Journal of the National Cancer Institute* **104**, 815-840 (2012).

7 Bao, Y. & Michaud, D. S. Physical activity and pancreatic cancer risk: a systematic review. *Cancer Epidemiology Biomarkers & Prevention* **17**, 2671-2682 (2008).

8 Barone, E., Corrado, A., Gemignani, F. & Landi, S. Environmental risk factors for pancreatic cancer: an update. *Archives of Toxicology* **90**, 2617-2642 (2016).

9 Barry, V. W. *et al.* Fitness vs. fatness on all-cause mortality: a meta-analysis. *Progress in cardiovascular diseases* **56**, 382-390 (2014).

10 Batty, G. D. Physical activity and coronary heart disease in older adults: a systematic review of epidemiological studies. *The European Journal of Public Health* **12**, 171-176 (2002).

11 Bauman, A. E. Updating the evidence that physical activity is good for health: an epidemiological review 2000–2003. *Journal of science and medicine in sport* **7**, 6-19 (2004).

12 Behrens, G. *et al.* The association between physical activity and gastroesophageal cancer: systematic review and meta-analysis. *European journal of epidemiology* **29**, 151-170 (2014).

13 Behrens, G. *et al.* Physical activity and risk of pancreatic cancer: a systematic review and meta-analysis. *European journal of epidemiology* **30**, 279-298 (2015).

14 Belvederi Murri, M. *et al.* Physical activity promotes health and reduces cardiovascular mortality in depressed populations: a literature overview. *International Journal of Environmental Research and Public Health* **17**, 5545 (2020).

15 Bennett, C. M. *et al.* Lifestyle factors and small intestine adenocarcinoma risk: a systematic review and meta-analysis. *Cancer epidemiology* **39**, 265-273 (2015).

16 Berlin, J. A. & Colditz, G. A. A meta-analysis of physical activity in the prevention of coronary heart disease. *American journal of epidemiology* **132**, 612-628 (1990).

17 Berzigotti, A., Saran, U. & Dufour, J. F. Physical activity and liver diseases. *Hepatology* **63**, 1026-1040 (2016).

18 Blair, S. N. & Brodney, S. Effects of physical inactivity and obesity on morbidity and mortality: current evidence and research issues. *Medicine and science in sports and exercise* **31**, S646-662 (1999).

19 Buffart, L. M. *et al.* Physical activity and the risk of developing lung cancer among smokers: a meta-analysis. *Journal of Science and Medicine in Sport* **17**, 67-71 (2014).

20 Canabrava, K. L. R., Amorim, P. R. d. S., Miranda, V. P. N., Priore, S. E. & Franceschini, S. d. C. C. Sedentary behavior and cardiovascular risk in children: a systematic review. *Revista Brasileira de Medicina do Esporte* **25**, 433-441 (2019).

21 CHA, J., Kim, J. & Hong, K. Association between types of physical activity and risk of ischemic heart disease based on US guidelines: A systematic review and meta-analysis. *medRxiv*, 2023.2003. 2006.23286885 (2023).

22 Cust, A. E., Armstrong, B. K., Friedenreich, C. M., Slimani, N. & Bauman, A. Physical activity and endometrial cancer risk: a review of the current evidence, biologic mechanisms and the quality of physical activity assessment methods. *Cancer Causes & Control* **18**, 243-258 (2007).

23 Chen, Y., Yu, C. & Li, Y. Physical activity and risks of esophageal and gastric cancers: a meta-analysis. *PloS one* **9**, e88082 (2014).

24 Colpani, V. *et al.* Lifestyle factors, cardiovascular disease and all-cause mortality in middle-aged and elderly women: a systematic review and meta-analysis. *European journal of epidemiology* **33**, 831-845 (2018).

25 Cooper, R., Kuh, D., Hardy, R. & Group, M. R. Objectively measured physical capability levels and mortality: systematic review and meta-analysis. *Bmj* **341** (2010).

26 Cunningham, C., O'Sullivan, R., Caserotti, P. & Tully, M. A. Consequences of physical inactivity in older adults: A systematic review of reviews and meta‐analyses. *Scandinavian journal of medicine & science in sports* **30**, 816-827 (2020).

27 de Souto Barreto, P., Rolland, Y., Vellas, B. & Maltais, M. Association of long-term exercise training with risk of falls, fractures, hospitalizations, and mortality in older adults: a systematic review and meta-analysis. *JAMA internal medicine* **179**, 394-405 (2019).

28 Desnoyers, A., Riesco, E., Fülöp, T. & Pavic, M. Physical activity and cancer: Update and literature review. *La Revue de medecine interne* **37**, 399-405 (2016).

29 Diep, L., Kwagyan, J., Kurantsin-Mills, J., Weir, R. & Jayam-Trouth, A. Association of physical activity level and stroke outcomes in men and women: a meta-analysis. *Journal of women's health* **19**, 1815-1822 (2010).

30 Dhote, R., Pellicer‐Coeuret, M., Thiounn, N., Debre, B. & Vidal‐Trecan, G. Risk factors for adult renal cell carcinoma: a systematic review and implications for prevention. *BJU international* **86**, 20-27 (2000).

31 Eaton, C. B. Relation of physical activity and cardiovascular fitness to coronary heart disease, part I: a meta-analysis of the independent relation of physical activity and coronary heart disease. *The Journal of the American Board of Family Practice* **5**, 31-42 (1992).

32 Ekelund, U. Infographic: Physical activity, sitting time and mortality. *British Journal of Sports Medicine* (2018).

33 Ekelund, U., Sanchez-Lastra, M. A., Dalene, K. E. & Tarp, J. Dose-response associations, physical activity intensity and mortality risk: A narrative review. *Journal of Sport and Health Science* (2023).

34 Erlichman, J., Kerbey, A. & James, W. Physical activity and its impact on health outcomes. Paper 1: the impact of physical activity on cardiovascular disease and all‐cause mortality: an historical perspective. *obesity reviews* **3**, 257-271 (2002).

35 Farris, M. S., Mosli, M. H., McFadden, A. A., Friedenreich, C. M. & Brenner, D. R. The association between leisure time physical activity and pancreatic cancer risk in adults: a systematic review and meta-analysis. *Cancer Epidemiology, Biomarkers & Prevention* **24**, 1462-1473 (2015).

36 Friedenreich, C. M. Physical activity and breast cancer: review of the epidemiologic evidence and biologic mechanisms. *Clinical cancer prevention*, 125-139 (2011).

37 Ferrario, M. M. *et al.* Differing associations for sport versus occupational physical activity and cardiovascular risk. *Heart* **104**, 1165-1172 (2018).

38 Fogelholm, M. Physical activity, fitness and fatness: relations to mortality, morbidity and disease risk factors. A systematic review. *Obesity reviews* **11**, 202-221 (2010).

39 Ford, E. S. & Caspersen, C. J. Sedentary behaviour and cardiovascular disease: a review of prospective studies. *International journal of epidemiology* **41**, 1338-1353 (2012).

40 Gallanagh, S., Quinn, T. J., Alexander, J. & Walters, M. R. Physical activity in the prevention and treatment of stroke. *International Scholarly Research Notices* **2011** (2011).

41 Garcia-Hermoso, A. *et al.* Safety and effectiveness of long-term exercise interventions in older adults: a systematic review and meta-analysis of randomized controlled trials. *Sports Medicine* **50**, 1095-1106 (2020).

42 Geidl, W., Schlesinger, S., Mino, E., Miranda, L. & Pfeifer, K. Dose–response relationship between physical activity and mortality in adults with noncommunicable diseases: a systematic review and meta-analysis of prospective observational studies. *International Journal of Behavioral Nutrition and Physical Activity* **17**, 1-18 (2020).

43 Gonçalves, A. K. *et al.* Effects of physical activity on breast cancer prevention: a systematic review. *Journal of Physical Activity and Health* **11**, 445-454 (2014).

44 Gonzalez-Jaramillo, N. *et al.* Systematic review of physical activity trajectories and mortality in patients with coronary artery disease. *Journal of the American College of Cardiology* **79**, 1690-1700 (2022).

45 Hackshaw-McGeagh, L. E. *et al.* A systematic review of dietary, nutritional, and physical activity interventions for the prevention of prostate cancer progression and mortality. *Cancer Causes & Control* **26**, 1521-1550 (2015).

46 Hall, K. S. *et al.* Systematic review of the prospective association of daily step counts with risk of mortality, cardiovascular disease, and dysglycemia. *International Journal of Behavioral Nutrition and Physical Activity* **17**, 1-14 (2020).

47 Harriss, D. *et al.* Lifestyle factors and colorectal cancer risk (2): a systematic review and meta‐analysis of associations with leisure‐time physical activity. *Colorectal Disease* **11**, 689-701 (2009).

48 Hershey, M. S., Martínez-González, M. Á., Álvarez-Álvarez, I., Hernández, J. A. M. & Ruiz-Canela, M. The Mediterranean diet and physical activity: better together than apart for the prevention of premature mortality. *British Journal of Nutrition* **128**, 1413-1424 (2022).

49 Katzmarzyk, P., Janssen, I. & Ardern, C. Physical inactivity, excess adiposity and premature mortality. *Obesity reviews* **4**, 257-290 (2003).

50 Keum, N. *et al.* Leisure‐time physical activity and endometrial cancer risk: Dose–response meta‐analysis of epidemiological studies. *International journal of cancer* **135**, 682-694 (2014).

51 Khan, H. *et al.* Resting heart rate and risk of incident heart failure: three prospective cohort studies and a systematic meta‐analysis. *Journal of the American Heart Association* **4**, e001364 (2015).

52 Kokkinos, P. Physical activity, health benefits, and mortality risk. *International Scholarly Research Notices* **2012** (2012).

53 Kraus, W. E. *et al.* Daily step counts for measuring physical activity exposure and its relation to health. *Medicine and science in sports and exercise* **51**, 1206 (2019).

54 Kyu, H. H. *et al.* Physical activity and risk of breast cancer, colon cancer, diabetes, ischemic heart disease, and ischemic stroke events: systematic review and dose-response meta-analysis for the Global Burden of Disease Study 2013. *bmj* **354** (2016).

55 Ku, P.-W., Steptoe, A., Liao, Y., Hsueh, M.-C. & Chen, L.-J. A cut-off of daily sedentary time and all-cause mortality in adults: a meta-regression analysis involving more than 1 million participants. *BMC medicine* **16**, 1-9 (2018).

56 Je, Y., Jeon, J. Y., Giovannucci, E. L. & Meyerhardt, J. A. Association between physical activity and mortality in colorectal cancer: a meta‐analysis of prospective cohort studies. *International journal of cancer* **133**, 1905-1913 (2013).

57 Jiménez-Pavón, D., Lavie, C. J. & Blair, S. N. The role of cardiorespiratory fitness on the risk of sudden cardiac death at the population level: a systematic review and meta-analysis of the available evidence. *Progress in Cardiovascular Diseases* **62**, 279-287 (2019).

58 Jarczok, M. N. *et al.* Heart rate variability in the prediction of mortality: A systematic review and meta-analysis of healthy and patient populations. *Neuroscience & Biobehavioral Reviews* **143**, 104907 (2022).

59 Lacombe, J., Armstrong, M. E., Wright, F. L. & Foster, C. The impact of physical activity and an additional behavioural risk factor on cardiovascular disease, cancer and all-cause mortality: a systematic review. *BMC Public Health* **19**, 1-16 (2019).

60 Lahart, I. M., Metsios, G. S., Nevill, A. M. & Carmichael, A. R. Physical activity, risk of death and recurrence in breast cancer survivors: a systematic review and meta-analysis of epidemiological studies. *Acta oncologica* **54**, 635-654 (2015).

61 Lavie, C. J. *et al.* in *Mayo Clinic Proceedings.* 1541-1552 (Elsevier).

62 Lagerros, Y., Hsieh, S. & Hsieh, C. Physical activity in adolescence and young adulthood and breast cancer risk: a quantitative review. *European Journal of Cancer Prevention*, 5-12 (2004).

63 Lemez, S. & Baker, J. Do elite athletes live longer? A systematic review of mortality and longevity in elite athletes. *Sports medicine-open* **1**, 1-14 (2015).

64 Lee, I.-M. & Skerrett, P. J. Physical activity and all-cause mortality: what is the dose-response relation? *Medicine & science in sports & exercise* **33**, S459-S471 (2001).

65 Lee, C. D., Folsom, A. R. & Blair, S. N. Physical activity and stroke risk: a meta-analysis. *Stroke* **34**, 2475-2481 (2003).

66 Lee, D.-C. *et al.* Running as a key lifestyle medicine for longevity. *Progress in cardiovascular diseases* **60**, 45-55 (2017).

67 Lee, J. Associations between physical activity and liver cancer risks and mortality: A systematic review and meta-analysis. *International Journal of Environmental Research and Public Health* **17**, 8943 (2020).

68 Leitzmann, M. *et al.* European code against cancer 4th edition: physical activity and cancer. *Cancer epidemiology* **39**, S46-S55 (2015).

69 Liang, Z.-d., Zhang, M., Wang, C.-z., Yuan, Y. & Liang, J.-h. Association between sedentary behavior, physical activity, and cardiovascular disease-related outcomes in adults—A meta-analysis and systematic review. *Frontiers in Public Health* **10**, 1018460 (2022).

70 Li, J. & Siegrist, J. Physical activity and risk of cardiovascular disease—a meta-analysis of prospective cohort studies. *International journal of environmental research and public health* **9**, 391-407 (2012).

71 Li, J., Loerbroks, A. & Angerer, P. Physical activity and risk of cardiovascular disease: what does the new epidemiological evidence show? *Current opinion in cardiology* **28**, 575-583 (2013).

72 Li, T. *et al.* The dose–response effect of physical activity on cancer mortality: findings from 71 prospective cohort studies. *British journal of sports medicine* **50**, 339-345 (2016).

73 Liu, Y. *et al.* Does physical activity reduce the risk of prostate cancer? A systematic review and meta-analysis. *European urology* **60**, 1029-1044 (2011).

74 Liu, L. *et al.* Leisure time physical activity and cancer risk: evaluation of the WHO's recommendation based on 126 high-quality epidemiological studies. *British journal of sports medicine* **50**, 372-378 (2016).

75 Loef, M. & Walach, H. The combined effects of healthy lifestyle behaviors on all cause mortality: a systematic review and meta-analysis. *Preventive medicine* **55**, 163-170 (2012).

76 J Tarraga Lopez, P., Solera Albero, J. & Antonio Rodriguez-Montes, J. Is it possible to reduce the incident of colorectal cancer by modifying diet and lifestyle? *Current Cancer Therapy Reviews* **9**, 157-163 (2013).

77 Lopez, J. P. R., Sabag, A., Juan, M. M., Rezende, L. F. & Pastor-Valero, M. Do vigorous-intensity and moderate-intensity physical activities reduce mortality to the same extent? A systematic review and meta-analysis. *BMJ Open Sport & Exercise Medicine* **6**, e000775 (2020).

78 Lynch, B. M. Sedentary behavior and cancer: a systematic review of the literature and proposed biological mechanisms. *Cancer Epidemiology, Biomarkers & Prevention* **19**, 2691-2709 (2010).

79 Lynch, B. M. & Leitzmann, M. F. An evaluation of the evidence relating to physical inactivity, sedentary behavior, and cancer incidence and mortality. *Current Epidemiology Reports* **4**, 221-231 (2017).

80 MacKinnon, H. J. *et al.* The association of physical function and physical activity with all-cause mortality and adverse clinical outcomes in nondialysis chronic kidney disease: a systematic review. *Therapeutic advances in chronic disease* **9**, 209-226 (2018).

81 Maisonneuve, P. & Lowenfels, A. B. Risk factors for pancreatic cancer: a summary review of meta-analytical studies. *International journal of epidemiology* **44**, 186-198 (2015).

82 Martins, P. *et al.* Association between physical activity and mortality in end-stage kidney disease: a systematic review of observational studies. *BMC nephrology* **22**, 227 (2021).

83 McTiernan, A. *et al.* Physical activity in cancer prevention and survival: a systematic review. *Medicine and science in sports and exercise* **51**, 1252 (2019).

84 Meneses-Echávez, J. F., González-Jiménez, E. & Ramírez-Vélez, R. Effects of supervised exercise on cancer-related fatigue in breast cancer survivors: a systematic review and meta-analysis. *BMC cancer* **15**, 1-13 (2015).

85 Michaud, D. S. *et al.* Physical activity, obesity, height, and the risk of pancreatic cancer. *Jama* **286**, 921-929 (2001).

86 Milton, K., Macniven, R. & Bauman, A. Review of the epidemiological evidence for physical activity and health from low-and middle-income countries. *Global public health* **9**, 369-381 (2014).

87 Monninkhof, E. M. *et al.* Physical activity and breast cancer: a systematic review. *epidemiology*, 137-157 (2007).

88 Moore, S., Gierach, G., Schatzkin, A. & Matthews, C. Physical activity, sedentary behaviours, and the prevention of endometrial cancer. *British journal of cancer* **103**, 933-938 (2010).

89 Naci, H. & Ioannidis, J. P. Comparative effectiveness of exercise and drug interventions on mortality outcomes: metaepidemiological study. *Bmj* **347**, f5577 (2013).

90 Nicolson, P. J., Duong, V., Williamson, E., Hopewell, S. & Lamb, S. E. The effect of therapeutic exercise interventions on physical and psychosocial outcomes in adults aged 80 years and older: A systematic review and Meta-analysis. *Journal of Aging and Physical Activity* **30**, 517-534 (2021).

91 Noguchi, J. L., Liss, M. A. & Parsons, J. K. Obesity, physical activity and bladder cancer. *Current urology reports* **16**, 1-13 (2015).

92 Oguma, Y., Sesso, H., Paffenbarger, R. & Lee, I. Physical activity and all cause mortality in women: a review of the evidence. *British journal of sports medicine* **36**, 162-172 (2002).

93 Oliveira, J. S. *et al.* Effect of sport on health in people aged 60 years and older: a systematic review with meta-analysis. *British Journal of Sports Medicine* **57**, 230-236 (2023).

94 Olsen, C. M. *et al.* Recreational physical activity and epithelial ovarian cancer: a case-control study, systematic review, and meta-analysis. *Cancer Epidemiology Biomarkers & Prevention* **16**, 2321-2330 (2007).

95 Oguma, Y. & Shinoda-Tagawa, T. Physical activity decreases cardiovascular disease risk in women: review and meta-analysis. *American journal of preventive medicine* **26**, 407-418 (2004).

96 O'Rorke, M. A., Cantwell, M. M., Cardwell, C. R., Mulholland, H. G. & Murray, L. J. Can physical activity modulate pancreatic cancer risk? A systematic review and meta‐analysis. *International journal of cancer* **126**, 2957-2968 (2010).

97 Pham, N. M. *et al.* Physical activity and colorectal cancer risk: an evaluation based on a systematic review of epidemiologic evidence among the Japanese population. *Japanese journal of clinical oncology* **42**, 2-13 (2012).

98 Patterson, R. *et al.* Sedentary behaviour and risk of all-cause, cardiovascular and cancer mortality, and incident type 2 diabetes: a systematic review and dose response meta-analysis. *European journal of epidemiology* **33**, 811-829 (2018).

99 Pandey, A. *et al.* Dose–response relationship between physical activity and risk of heart failure: a meta-analysis. *Circulation* **132**, 1786-1794 (2015).

100 Perreault, K. *et al.* Does physical activity moderate the association between alcohol drinking and all-cause, cancer and cardiovascular diseases mortality? A pooled analysis of eight British population cohorts. *British Journal of Sports Medicine* **51**, 651-657 (2017).

101 Prince, S. A. *et al.* The effect of leisure time physical activity and sedentary behaviour on the health of workers with different occupational physical activity demands: a systematic review. *International Journal of Behavioral Nutrition and Physical Activity* **18**, 1-17 (2021).

102 Ponticelli, C. & Favi, E. Physical inactivity: a modifiable risk factor for morbidity and mortality in kidney transplantation. *Journal of Personalized Medicine* **11**, 927 (2021).

103 Pozuelo-Carrascosa, D. P. *et al.* Resting heart rate as a predictor of cancer mortality: a systematic review and meta-analysis. *Journal of Clinical Medicine* **10**, 1354 (2021).

104 Proper, K. I., Singh, A. S., Van Mechelen, W. & Chinapaw, M. J. Sedentary behaviors and health outcomes among adults: a systematic review of prospective studies. *American journal of preventive medicine* **40**, 174-182 (2011).

105 Rahmati, M. *et al.* Baseline physical activity is associated with reduced mortality and disease outcomes in COVID‐19: A systematic review and meta‐analysis. *Reviews in medical virology* **32**, e2349 (2022).

106 Ramsey, K. A., Meskers, C. G. & Maier, A. B. Every step counts: synthesising reviews associating objectively measured physical activity and sedentary behaviour with clinical outcomes in community-dwelling older adults. *The Lancet Healthy Longevity* **2**, e764-e772 (2021).

107 Reimers, C. D., Knapp, G. & Reimers, A. K. Does physical activity increase life expectancy? A review of the literature. *Journal of aging research* **2012** (2012).

108 Ristow, B., Doubell, A., Derman, W. & Heine, M. Change in resting heart rate and risk for all-cause mortality. *European Journal of Preventive Cardiology* **29**, e249-e254 (2022).

109 Robsahm, T. E. *et al.* Body mass index, physical activity, and colorectal cancer by anatomical subsites. *European Journal of Cancer Prevention* **22**, 492-505 (2013).

110 Rocha, V., Paixão, C. & Marques, A. Physical activity, exercise capacity and mortality risk in people with interstitial lung disease: A systematic review and meta-analysis. *Journal of Science and Medicine in Sport* (2022).

111 Rossi, A., Dikareva, A., Bacon, S. L. & Daskalopoulou, S. S. The impact of physical activity on mortality in patients with high blood pressure: a systematic review. *Journal of hypertension* **30**, 1277-1288 (2012).

112 Samad, A., Taylor, R., Marshall, T. & Chapman, M. A. A meta‐analysis of the association of physical activity with reduced risk of colorectal cancer. *Colorectal disease* **7**, 204-213 (2005).

113 Sattelmair, J. *et al.* Dose response between physical activity and risk of coronary heart disease: a meta-analysis. *Circulation* **124**, 789-795 (2011).

114 Sergentanis, T. N. *et al.* Risk factors for multiple myeloma: a systematic review of meta-analyses. *Clinical Lymphoma Myeloma and Leukemia* **15**, 563-577. e563 (2015).

115 Schmid, D. & Leitzmann, M. F. Television viewing and time spent sedentary in relation to cancer risk: a meta-analysis. *JNCI: Journal of the National Cancer Institute* **106**, dju098 (2014).

116 Schmid, D., Ricci, C., Behrens, G. & Leitzmann, M. F. Does smoking influence the physical activity and lung cancer relation? A systematic review and meta-analysis. *European journal of epidemiology* **31**, 1173-1190 (2016).

117 Schnohr, P. Physical activity in leisure time: impact on mortality. *Dan Med Bull* **56**, 40-71 (2009).

118 Singh, S., Devanna, S., Edakkanambeth Varayil, J., Murad, M. H. & Iyer, P. G. Physical activity is associated with reduced risk of esophageal cancer, particularly esophageal adenocarcinoma: a systematic review and meta-analysis. *BMC gastroenterology* **14**, 1-11 (2014).

119 Sittichai, N. *et al.* Effects of physical activity on the severity of illness and mortality in COVID-19 patients: A systematic review and meta-analysis. *Frontiers in Physiology* **13**, 1030568 (2022).

120 Sofi, F., Capalbo, A., Cesari, F., Abbate, R. & Gensini, G. F. Physical activity during leisure time and primary prevention of coronary heart disease: an updated meta-analysis of cohort studies. *European Journal of Cardiovascular Prevention & Rehabilitation* **15**, 247-257 (2008).

121 Spence, R., Heesch, K. & Brown, W. A systematic review of the association between physical activity and colorectal cancer risk. *Scandinavian journal of medicine & science in sports* **19**, 764-781 (2009).

122 Sun, J.-Y., Shi, L., Gao, X.-D. & Xu, S.-F. Physical activity and risk of lung cancer: a meta-analysis of prospective cohort studies. *Asian Pacific Journal of Cancer Prevention* **13**, 3143-3147 (2012).

123 Tajabadi, Z., Akbari, M. & Hafez, A. Physical activity and gastrointestinal cancer risk: a review. *Acta Medica Bulgarica* **46**, 57-67 (2019).

124 Tardon, A. *et al.* Leisure-time physical activity and lung cancer: a meta-analysis. *Cancer Causes & Control* **16**, 389-397 (2005).

125 Taylor, W. C., Rix, K., Gibson, A. & Paxton, R. J. Sedentary behavior and health outcomes in older adults: A systematic review. *AIMS Medical Science* **7** (2020).

126 Teramoto, M. & Bungum, T. J. Mortality and longevity of elite athletes. *Journal of Science and Medicine in Sport* **13**, 410-416 (2010).

127 Thorp, A. A., Owen, N., Neuhaus, M. & Dunstan, D. W. Sedentary behaviors and subsequent health outcomes in adults: a systematic review of longitudinal studies, 1996–2011. *American journal of preventive medicine* **41**, 207-215 (2011).

128 Tolley, A. P., Ramsey, K. A., Rojer, A. G., Reijnierse, E. M. & Maier, A. B. Objectively measured physical activity is associated with frailty in community-dwelling older adults: A systematic review. *Journal of clinical epidemiology* **137**, 218-230 (2021).

129 Van Uffelen, J. G. *et al.* Occupational sitting and health risks: a systematic review. *American journal of preventive medicine* **39**, 379-388 (2010).

130 Vermaete, N. V. *et al.* Physical activity and risk of lymphoma: a meta-analysis. *Cancer epidemiology, biomarkers & prevention* **22**, 1173-1184 (2013).

131 Volaklis, K. A., Halle, M. & Meisinger, C. Muscular strength as a strong predictor of mortality: a narrative review. *European journal of internal medicine* **26**, 303-310 (2015).

132 Voskuil, D. W. *et al.* Physical activity and endometrial cancer risk, a systematic review of current evidence. *Cancer Epidemiology Biomarkers & Prevention* **16**, 639-648 (2007).

133 Wahid, A. *et al.* Quantifying the association between physical activity and cardiovascular disease and diabetes: a systematic review and meta‐analysis. *Journal of the American Heart Association* **5**, e002495 (2016).

134 Wendel-Vos, G. *et al.* Physical activity and stroke. A meta-analysis of observational data. *International journal of epidemiology* **33**, 787-798 (2004).

135 Wang, Z. *et al.* Sedentary behavior and the risk of stroke: A systematic review and dose-response meta-analysis. *Nutrition, Metabolism and Cardiovascular Diseases* (2022).

136 Warburton, D. E., Charlesworth, S., Ivey, A., Nettlefold, L. & Bredin, S. S. A systematic review of the evidence for Canada's Physical Activity Guidelines for Adults. *International journal of behavioral nutrition and physical activity* **7**, 1-220 (2010).

137 Warburton, D. E. & Bredin, S. S. Health benefits of physical activity: a systematic review of current systematic reviews. *Current opinion in cardiology* **32**, 541-556 (2017).

138 Wu, Y. *et al.* How to Keep the Balance between Red and Processed Meat Intake and Physical Activity Regarding Mortality: A Dose-Response Meta-Analysis. *Nutrients* **15**, 3373 (2023).

139 Vogel, T. *et al.* Health benefits of physical activity in older patients: a review. *International journal of clinical practice* **63**, 303-320 (2009).

140 Xie, B. *et al.* Accelerometer-measured light-intensity physical activity and the risk of cardiovascular disease or death in older adults: A meta-analysis. *Kardiologia Polska (Polish Heart Journal)* **80**, 774-781 (2022).

141 Xu, C. *et al.* Sedentary behavior, physical activity, and all-cause mortality: dose-response and intensity weighted time-use meta-analysis. *Journal of the American Medical Directors Association* **20**, 1206-1212. e1203 (2019).

142 Yang, Y. *et al.* Physical activity and sedentary behaviour over adulthood in relation to all-cause and cause-specific mortality: a systematic review of analytic strategies and study findings. *International Journal of Epidemiology* **51**, 641-667 (2022).

143 Yerrakalva, D., Mullis, R. & Mant, J. The associations of “fatness,”“fitness,” and physical activity with all‐cause mortality in older adults: a systematic review. *Obesity* **23**, 1944-1956 (2015).

144 Zelenovic, M. *et al.* The effects of physical activity on disease and mortality. *International Journal of Sport Culture and Science* **9**, 255-267 (2021).

145 Zelenović, M. *et al.* Leisure-time physical activity and all-cause mortality: A systematic review. *Revista de Psicología del Deporte* **31** (2022).

146 Wu, Y., Zhang, D. & Kang, S. Physical activity and risk of breast cancer: a meta-analysis of prospective studies. *Breast cancer research and treatment* **137**, 869-882 (2013).

147 Zhang, D., Shen, X. & Qi, X. Resting heart rate and all-cause and cardiovascular mortality in the general population: a meta-analysis. *Cmaj* **188**, E53-E63 (2016).

148 Zhang, F., Wang, H., Wang, W. & Zhang, H. The role of physical activity and mortality in hemodialysis patients: a review. *Frontiers in Public Health* **10**, 818921 (2022).

149 Zhao, R., Bu, W., Chen, Y. & Chen, X. The dose-response associations of sedentary time with chronic diseases and the risk for all-cause mortality affected by different health status: a systematic review and meta-analysis. *The journal of nutrition, health & aging* **24**, 63-70 (2020).

150 Zheng, G. *et al.* Tai chi chuan for the primary prevention of stroke in middle-aged and elderly adults: a systematic review. *Evidence-Based Complementary and Alternative Medicine* **2015** (2015).

151 Zhong, S. *et al.* Association between physical activity and mortality in breast cancer: a meta-analysis of cohort studies. *European journal of epidemiology* **29**, 391-404 (2014).

152 Zhong, S. *et al.* Physical activity and risk of lung cancer: a meta-analysis. *Clinical Journal of Sport Medicine* **26**, 173-181 (2016).

153 Zhou, L.-M. Recreational physical activity and risk of ovarian cancer: a meta-analysis. *Asian Pacific Journal of Cancer Prevention* **15**, 5161-5166 (2014).

154 Zhuo, C., Zhao, J., Chen, M. & Lu, Y. Physical activity and risks of cardiovascular diseases: a mendelian randomization study. *Frontiers in Cardiovascular Medicine* **8**, 722154 (2021).

# **Figure 1.** Meta-regression analysis for the association between physical activity and all-cause mortality based on the moderating role of age.

# **Figure 2.** Meta-regression analysis for the association between physical activity and CVD mortality based on the moderating role of age.

# **Figure 3.** Meta-regression analysis for the association between physical activity and cancer mortality based on the moderating role of age.

# **Figure 4.** Forest plot of the association between different level of LTPA and all-cause mortality.


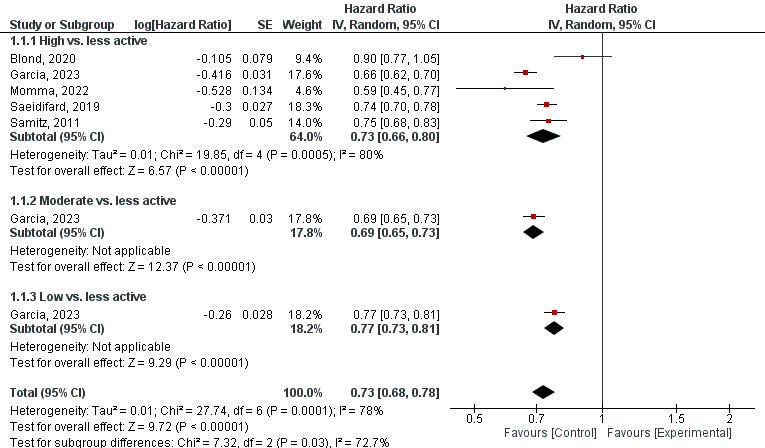


# **Figure 5.** Forest plot of the association between different level of LTPA and CVD mortality.


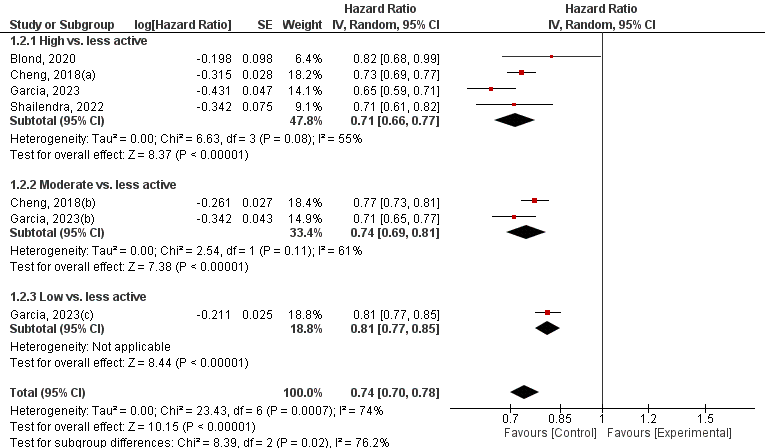


# **Figure 6.** Forest plot of the association between different level of LTPA and cancer mortality.


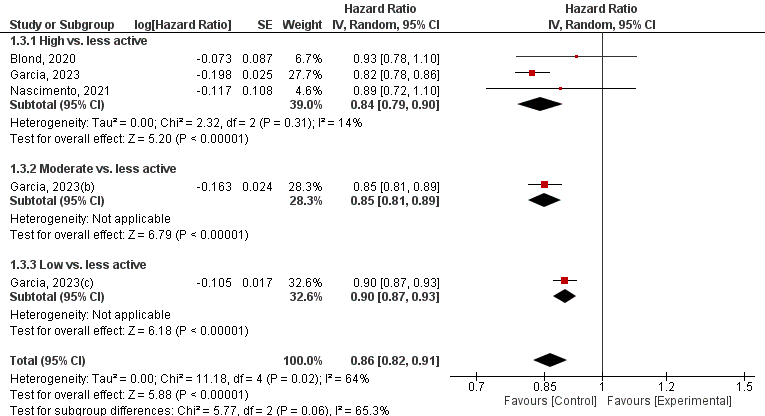


# **Figure 7.** Forest plot of the association between different level of device-measured TPA and cancer mortality.


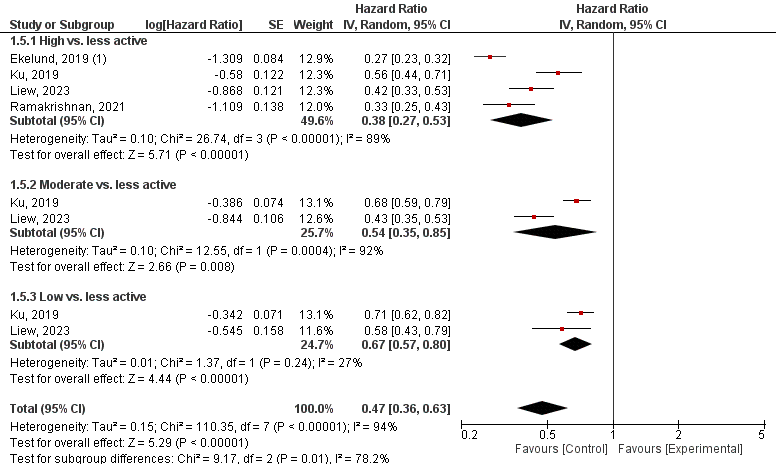


# **Figure 8.** Forest plot of the association between different level of self-reported TPA and cancer mortality.


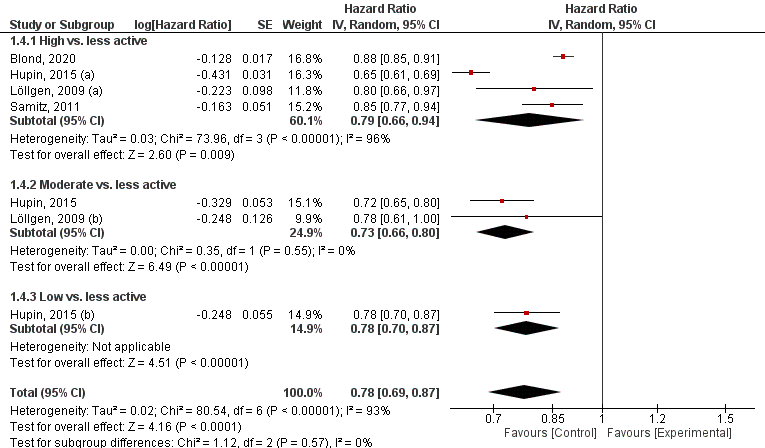


# **Figure 9.** Forest plot of the association between different level of PA and all-cause mortality in pooled analysis studies.


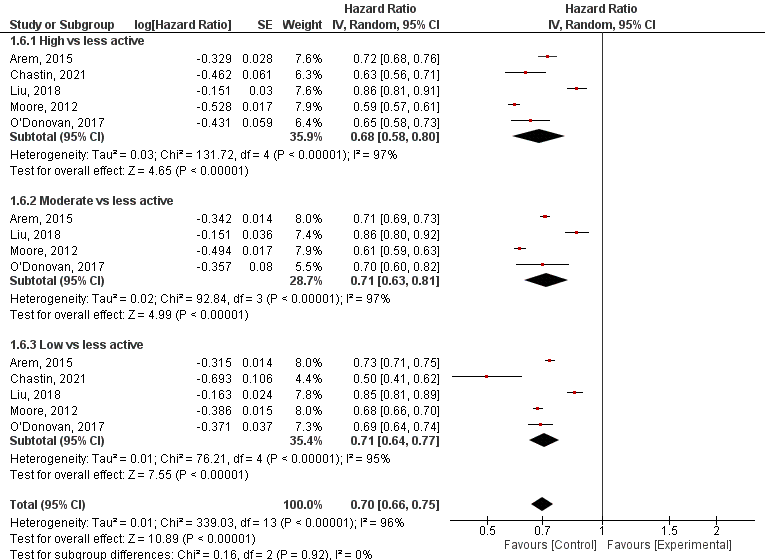


# **Figure 10.** Forest plot of the association between different level of PA and CVD mortality in pooled analysis studies.


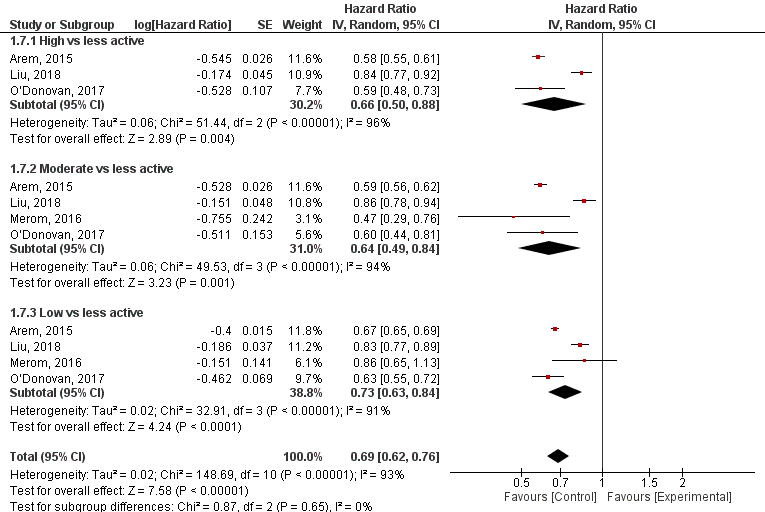


# **Figure 11.** Forest plot of the association between different level of PA and cancer mortality in pooled analysis studies.


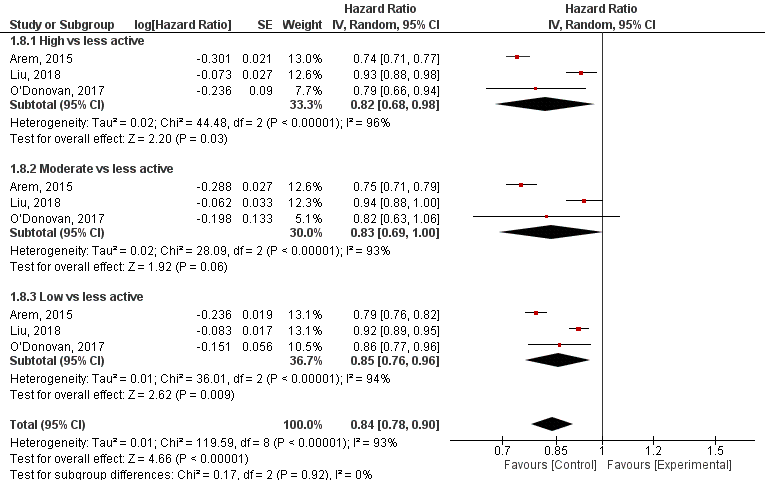


# **Figure 12.** Forest plot of the association between different measurement methods and all-cause mortality in pooled analysis studies.


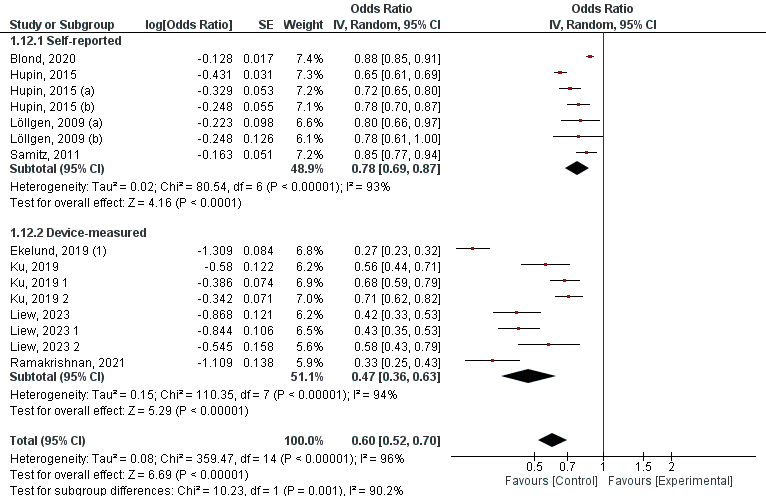


# **Figure 13.** Meta-regression analysis for the association between total physical activity and cancer mortality based on the moderating role of measurement methods.

# **Figure 14.** Forest plot of the association between different exercise training and all-cause mortality.


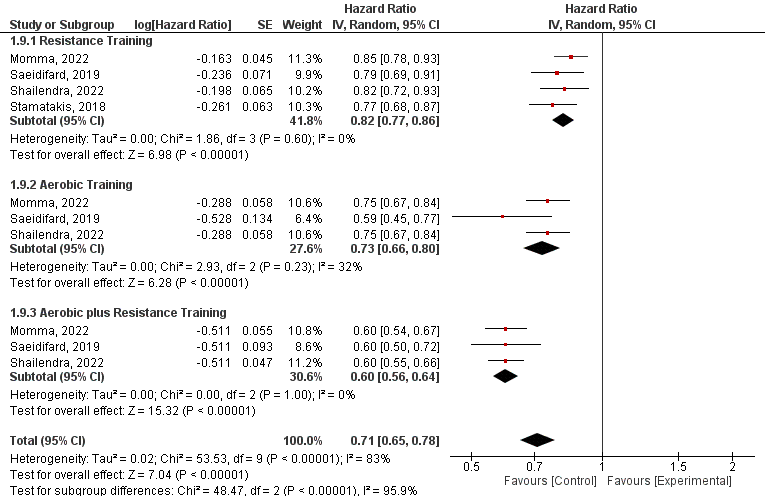


# **Figure 15.** Forest plot of the association between different exercise training and CVD mortality.


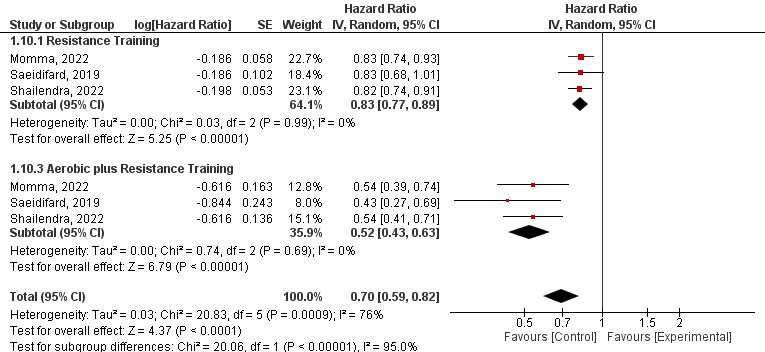


# **Figure 16.** Forest plot of the association between different exercise training and cancer mortality.


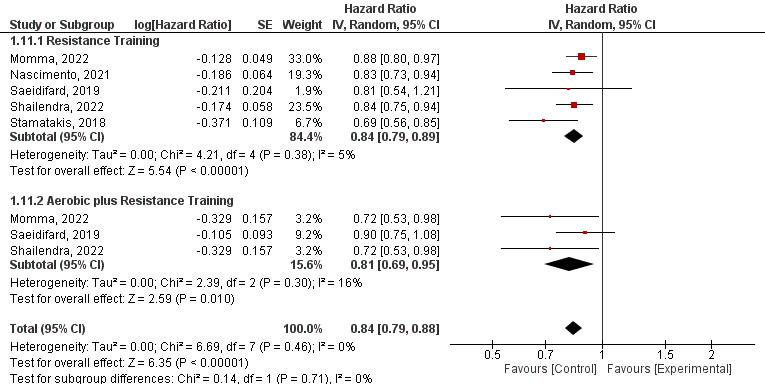


# **Figure 17.** Forest plot of the association between self-reported SB with CVD mortality based on different follow-up duration.


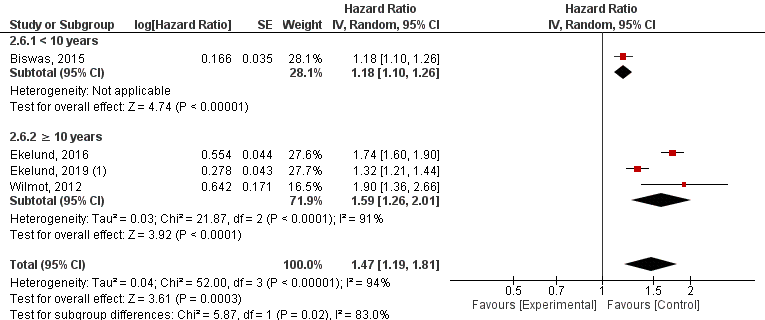


# **Figure 18.** Forest plot of the association between self-reported LTPA with all-cause mortality based on different follow-up duration.


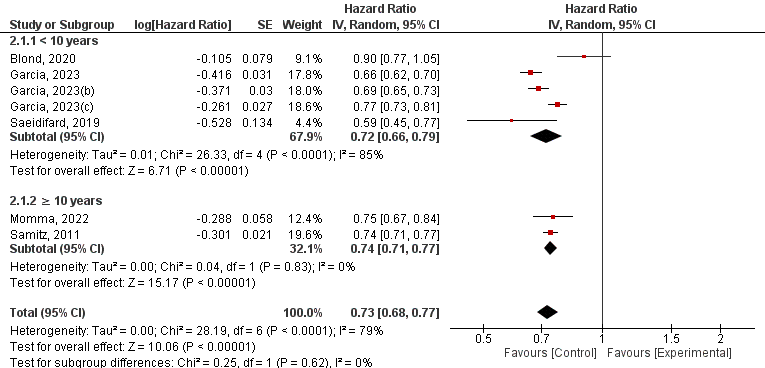


# **Figure 19.** Forest plot of the association between self-reported LTPA with CVD mortality based on different follow-up duration.


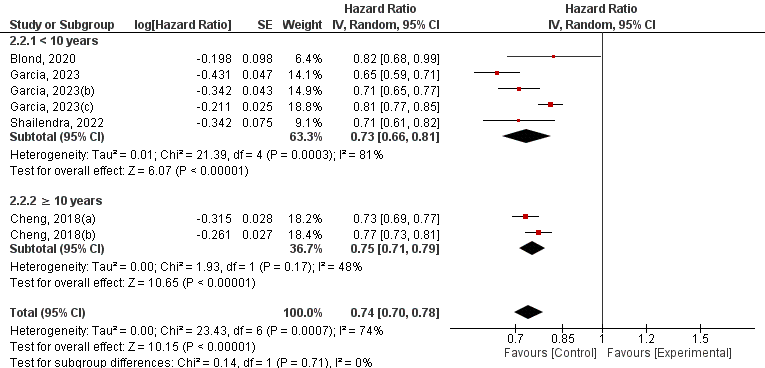


# **Figure 20.** Forest plot of the association between self-reported LTPA with cancer mortality based on different follow-up duration.


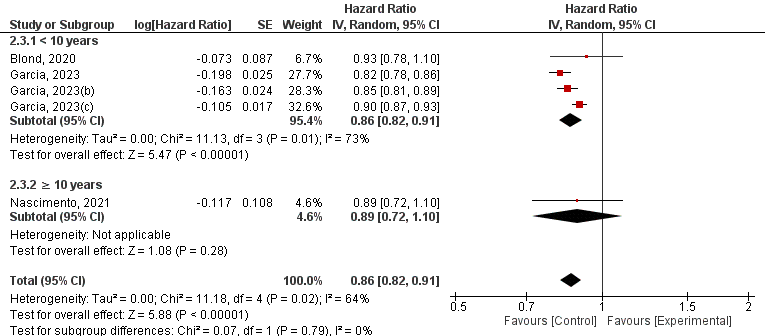


# **Figure 21.** Forest plot of the association between self-reported TPA with all-cause mortality based on different follow-up duration.


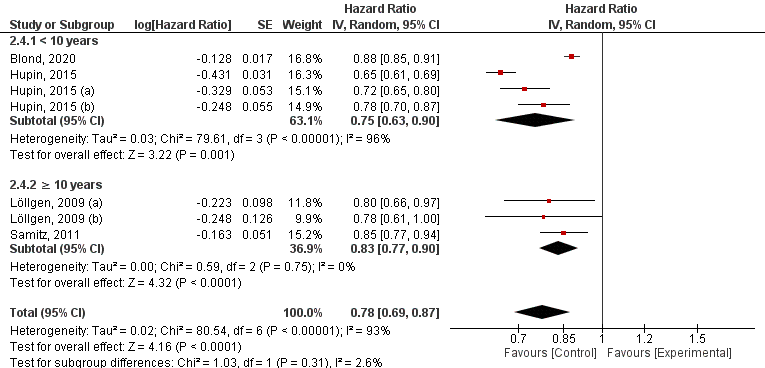


# **Figure 22.** Forest plot of the association between self-reported SB with all-cause mortality based on different follow-up duration.


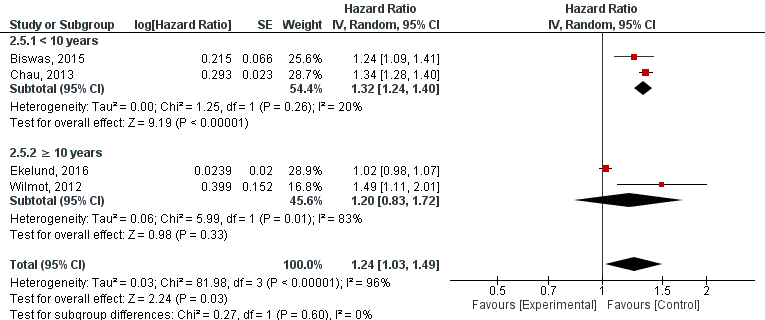


# **Figure 23.** Forest plot of the association between self-reported SB with CVD mortality based on different follow-up duration.


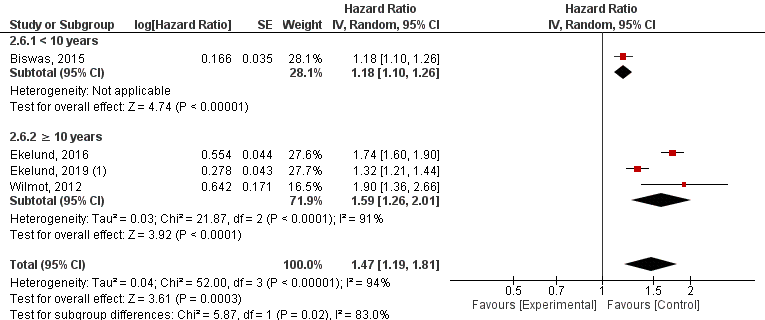


# **Figure 24.** Forest plot of the association between self-reported SB with cancer mortality based on different follow-up duration.


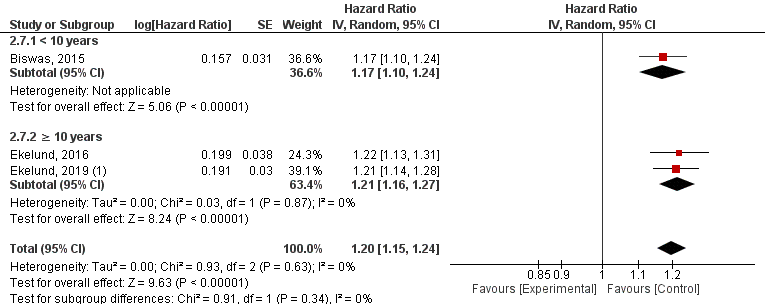


# **Figure 25.** Forest plot of the association between self-reported PA with all-cause mortality based on different follow-up duration in pooled analysis studies.


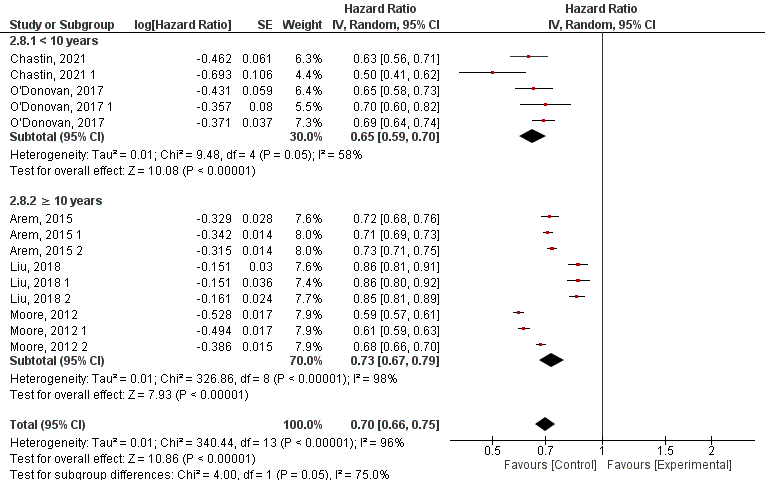


# **Figure 26.** Forest plot of the association between self-reported PA with CVD mortality based on different follow-up duration in pooled analysis studies.


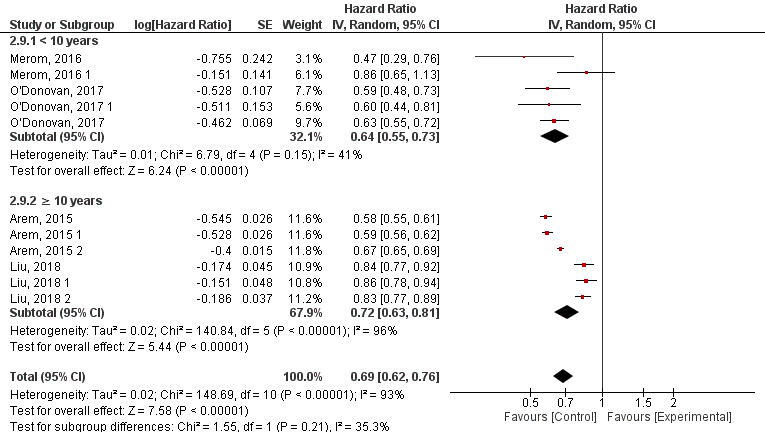


# **Figure 27.** Forest plot of the association between self-reported PA with cancer mortality based on different follow-up duration in pooled analysis studies.


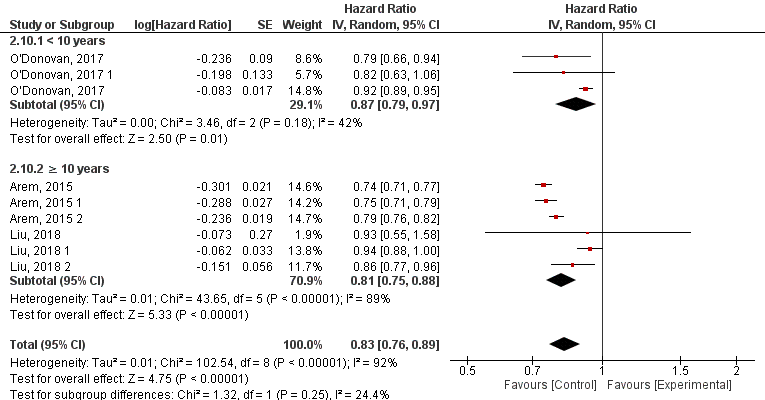


# **Figure 28.** P-curve analysis for self-reported leisure-time physical activity (LTPA).

1. All-cause mortality


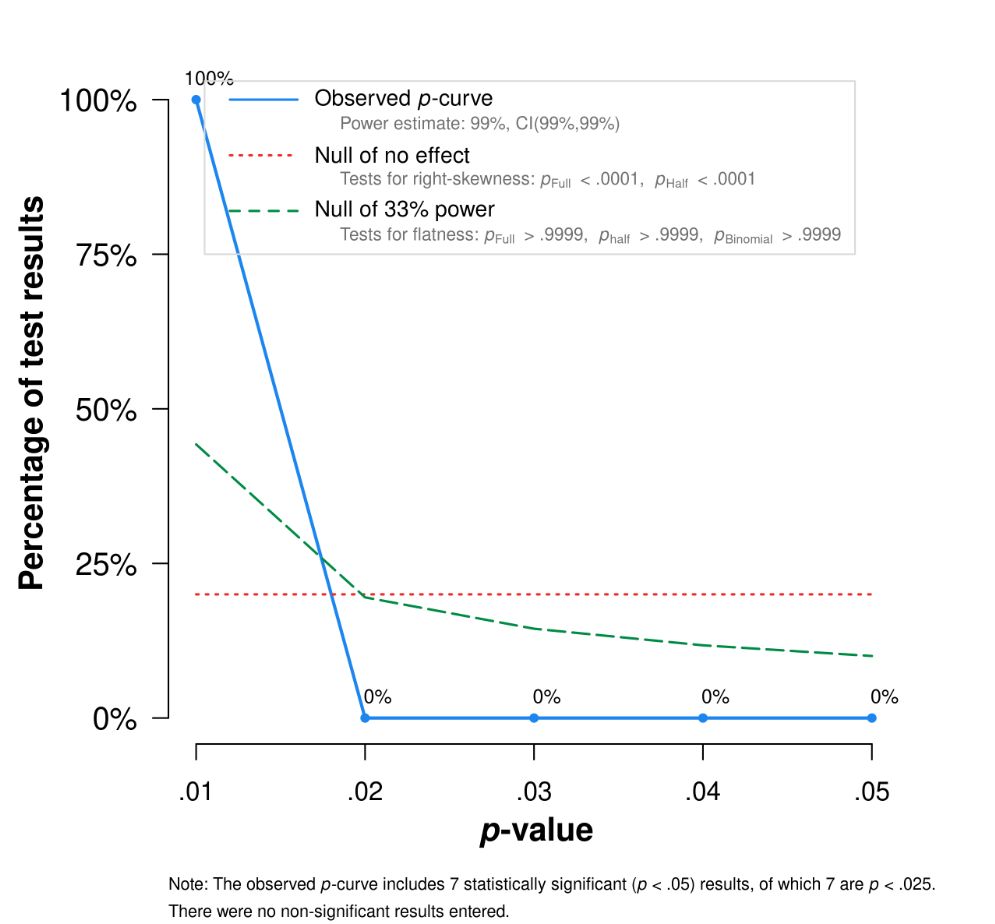


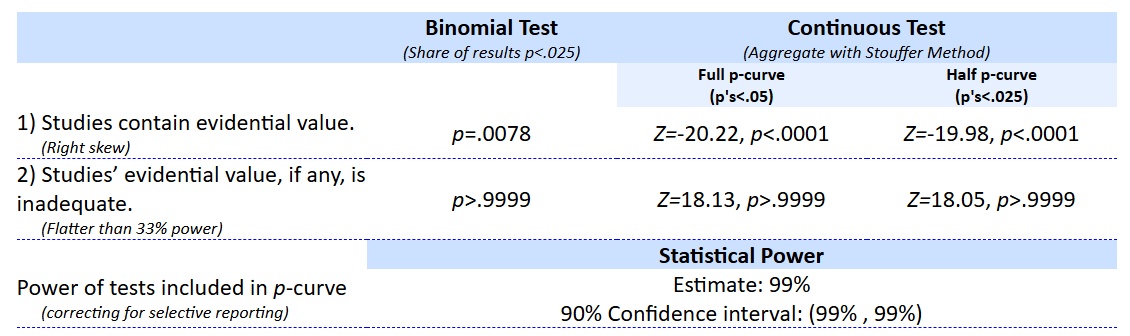


1. Cardiovascular disease mortality


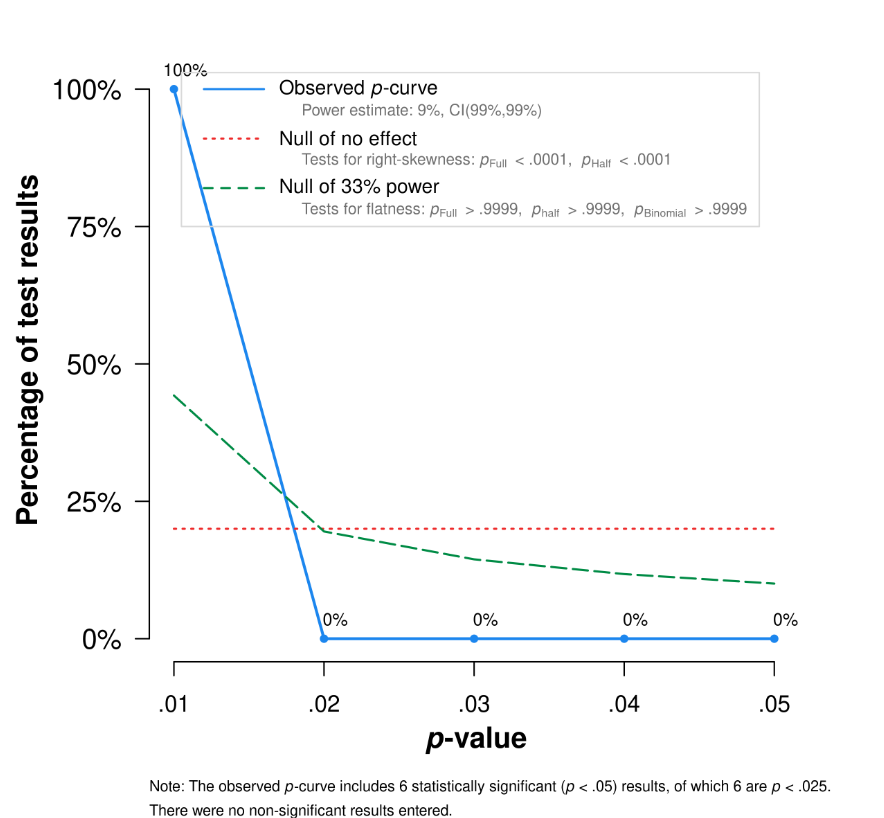


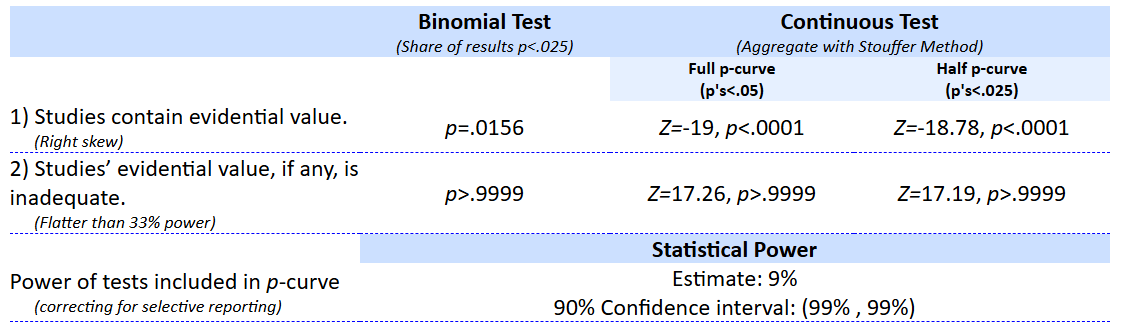


1. Cancer mortality


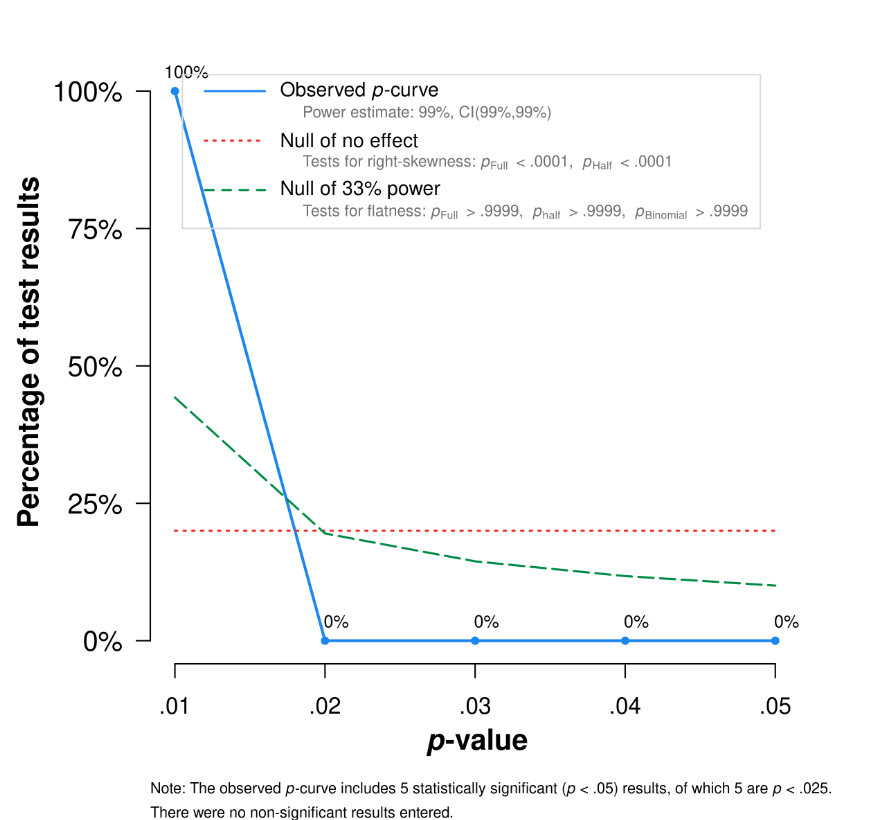

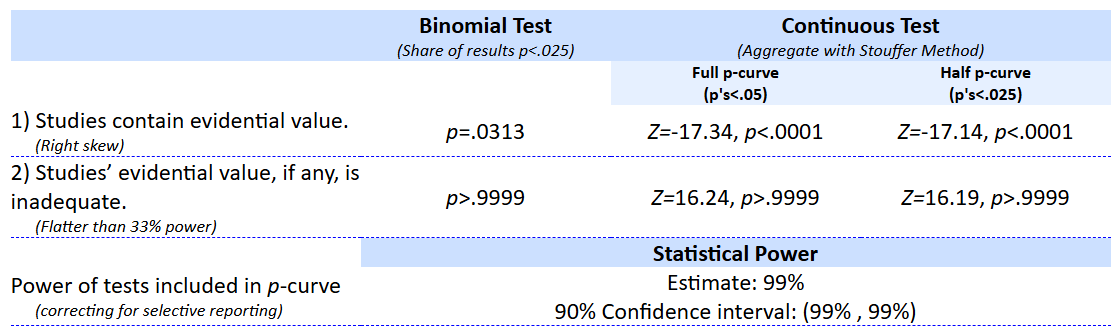


# **Figure 29.** P-curve analysis for Self-reported total physical activity (TPA).

1. All-cause mortality


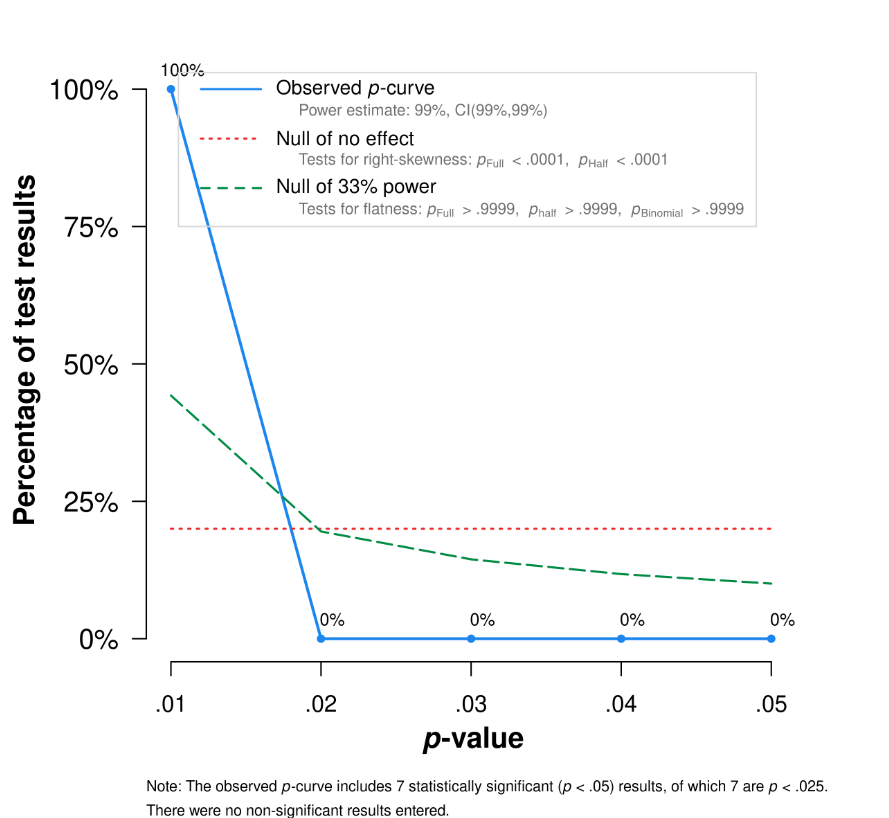


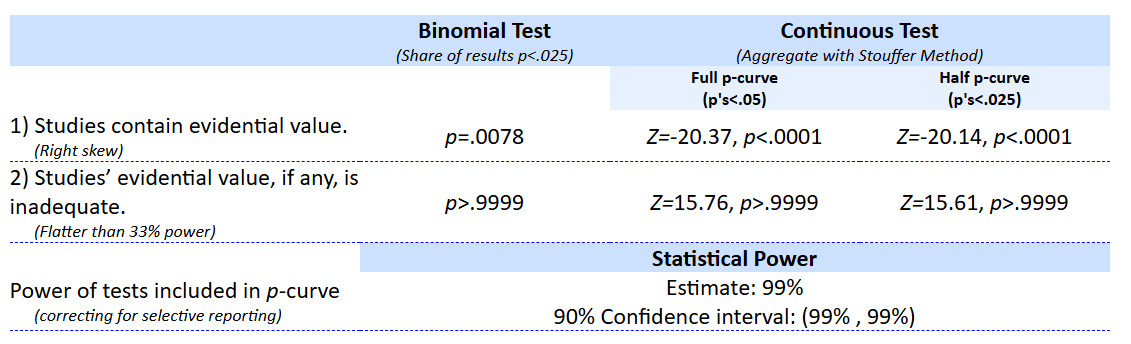


# **Figure 30.** P-curve analysis for device-measured total physical activity (TPA).

1. All-cause mortality


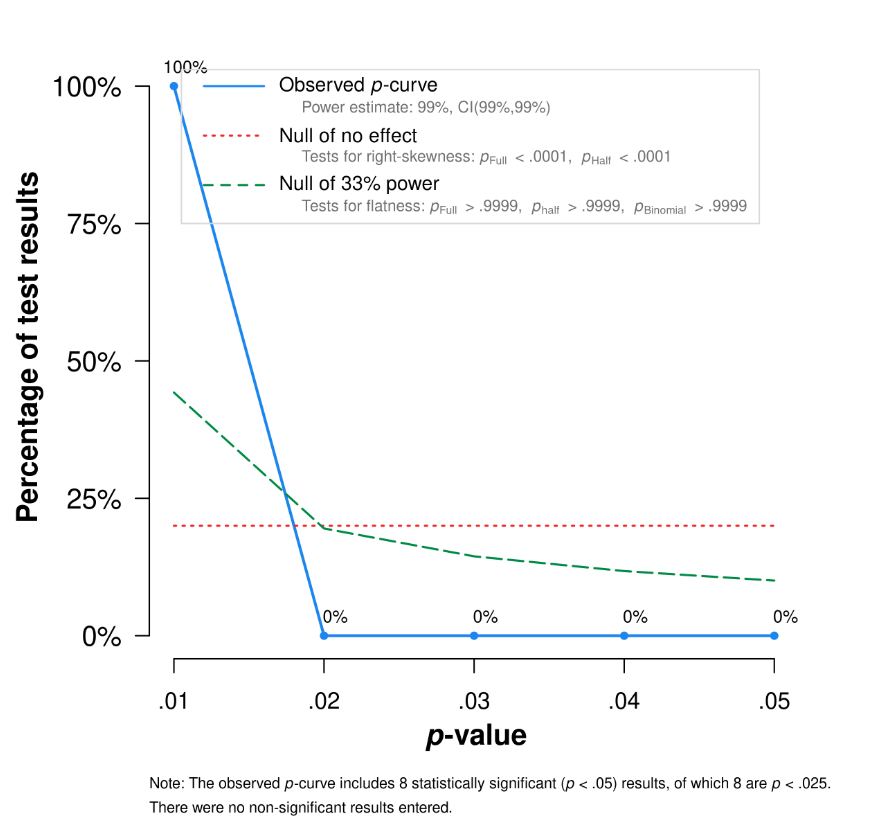

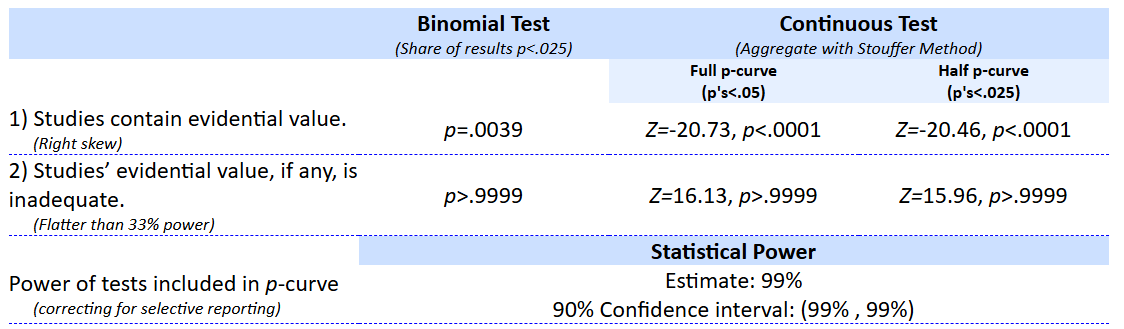


# **Figure 31.** P-curve analysis for self-reported sedentary behavior (SB).

1. Cardiovascular disease mortality


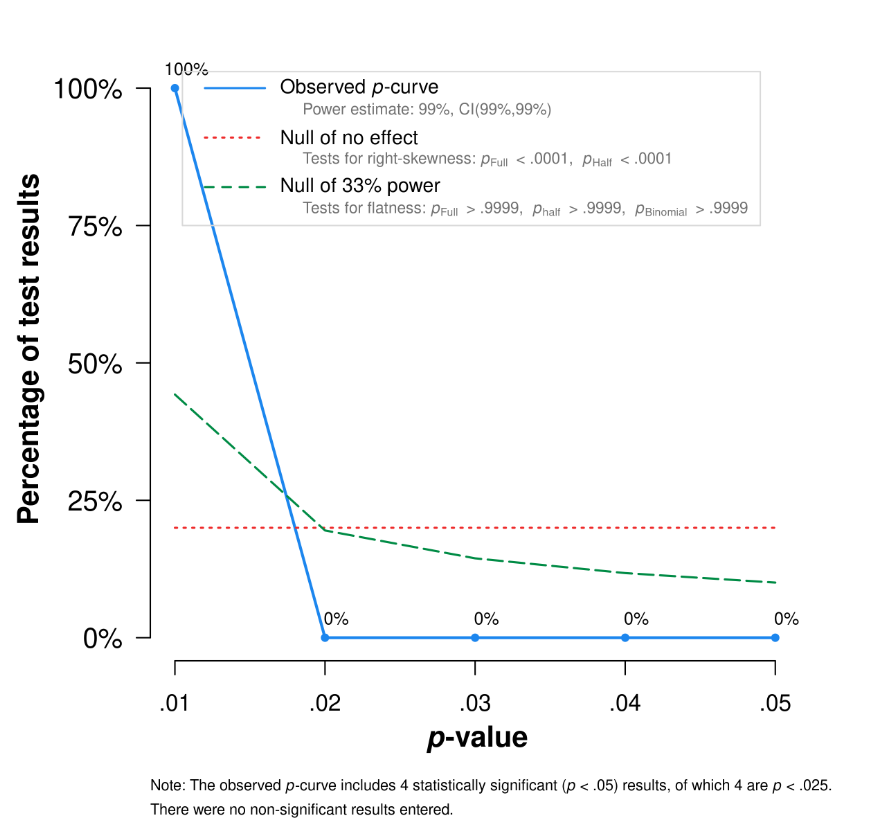

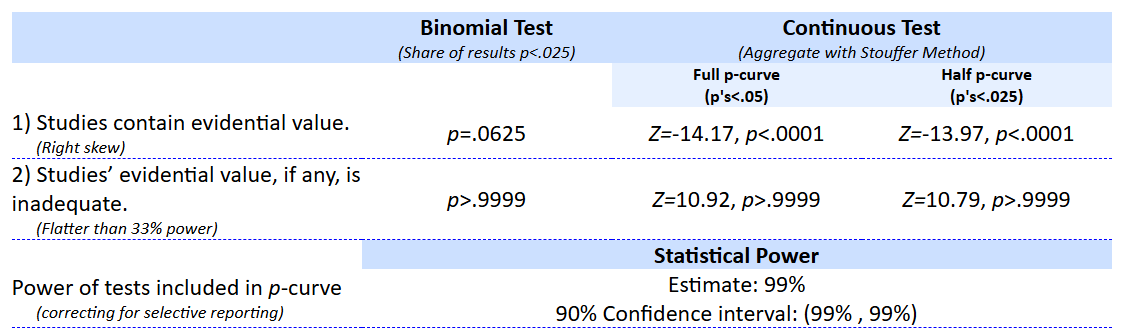


# **Figure 32.** P-curve analysis for device-measured sedentary behavior (SB).

1. All-cause mortality


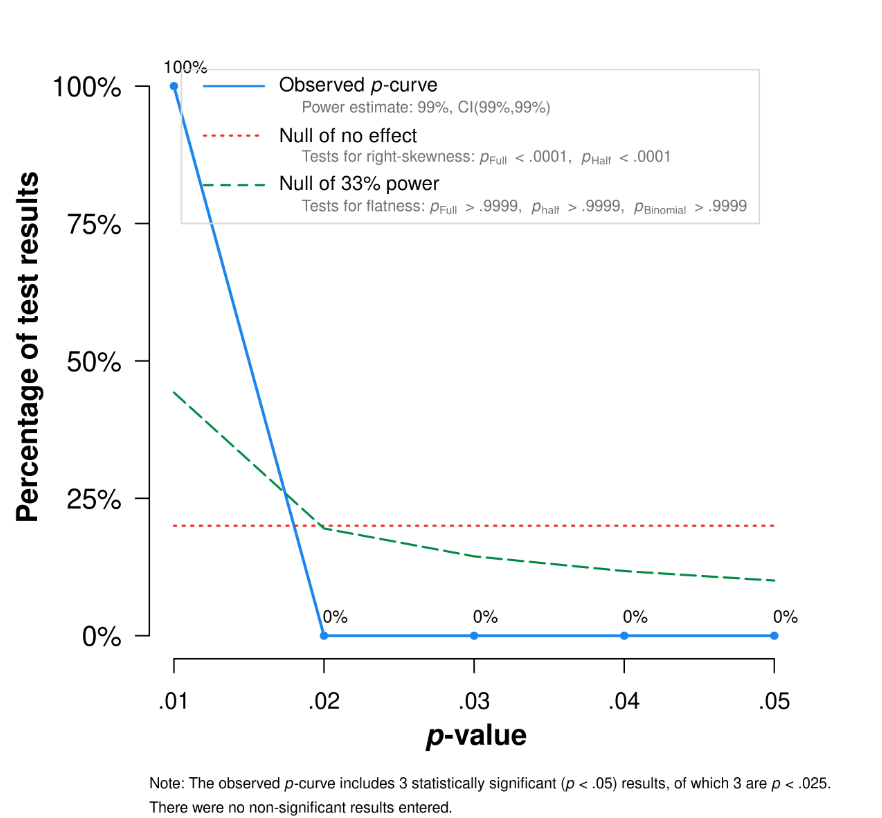

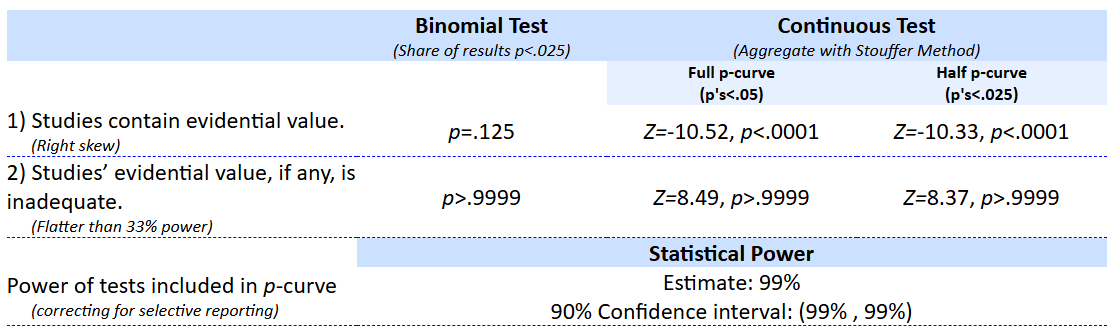


# **Figure 33.** P-curve analysis for self-reported walking time.

1. All-cause mortality


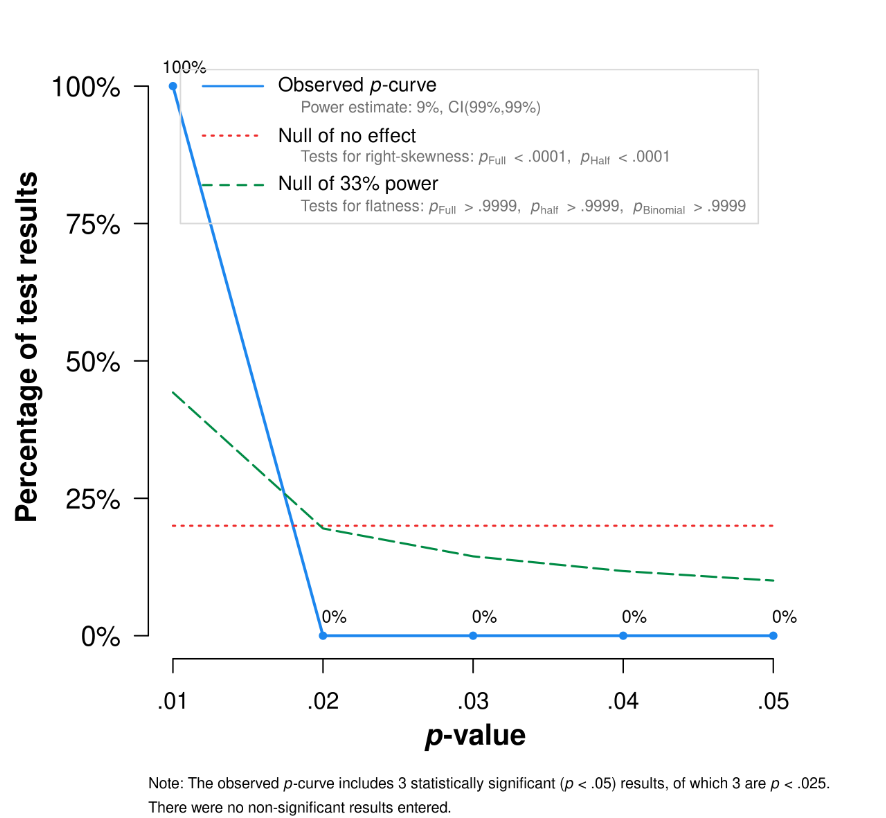

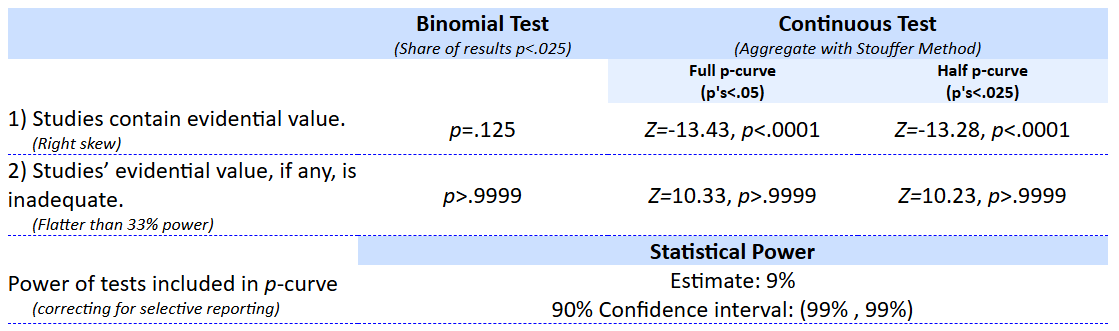


# **Figure 34.** P-curve analysis for self-reported running time.

1. All-cause mortality


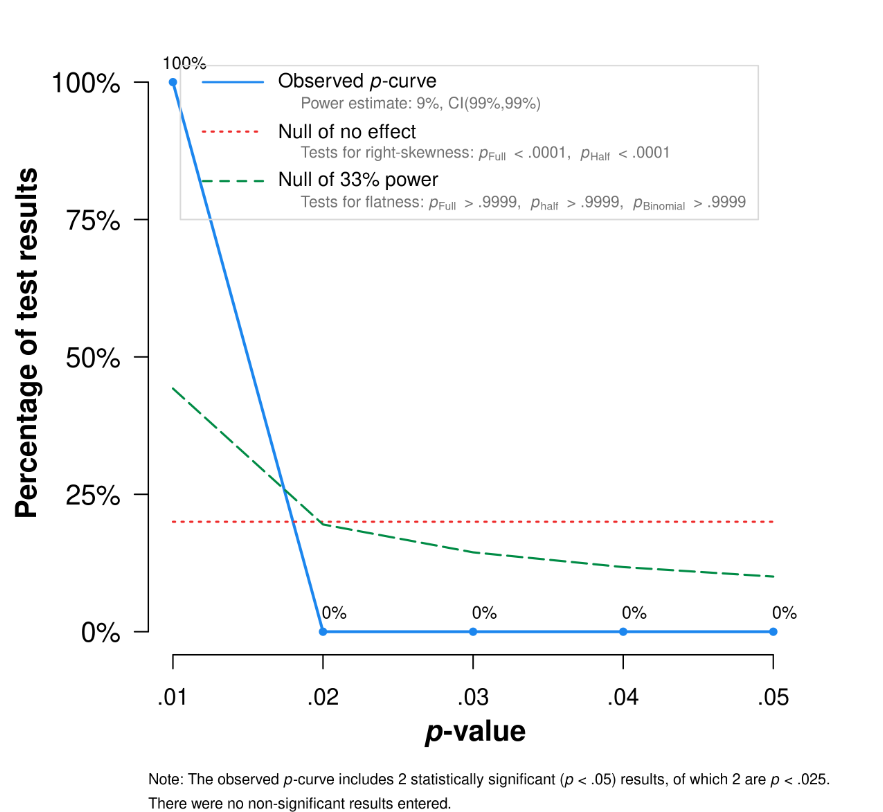

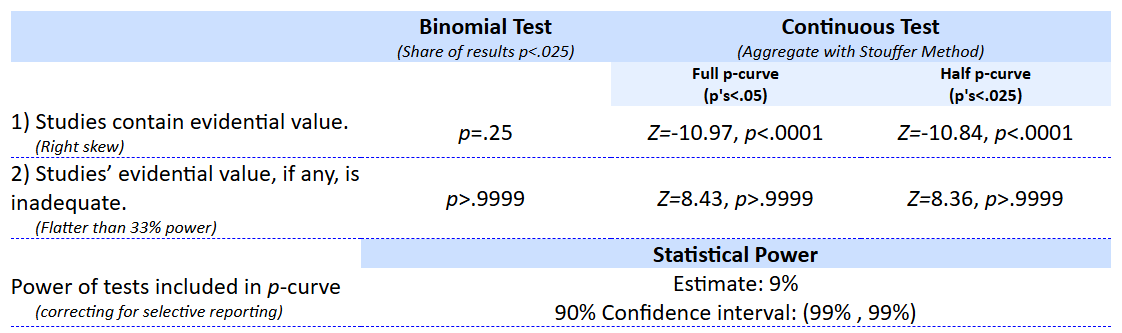


1. Cancer mortality


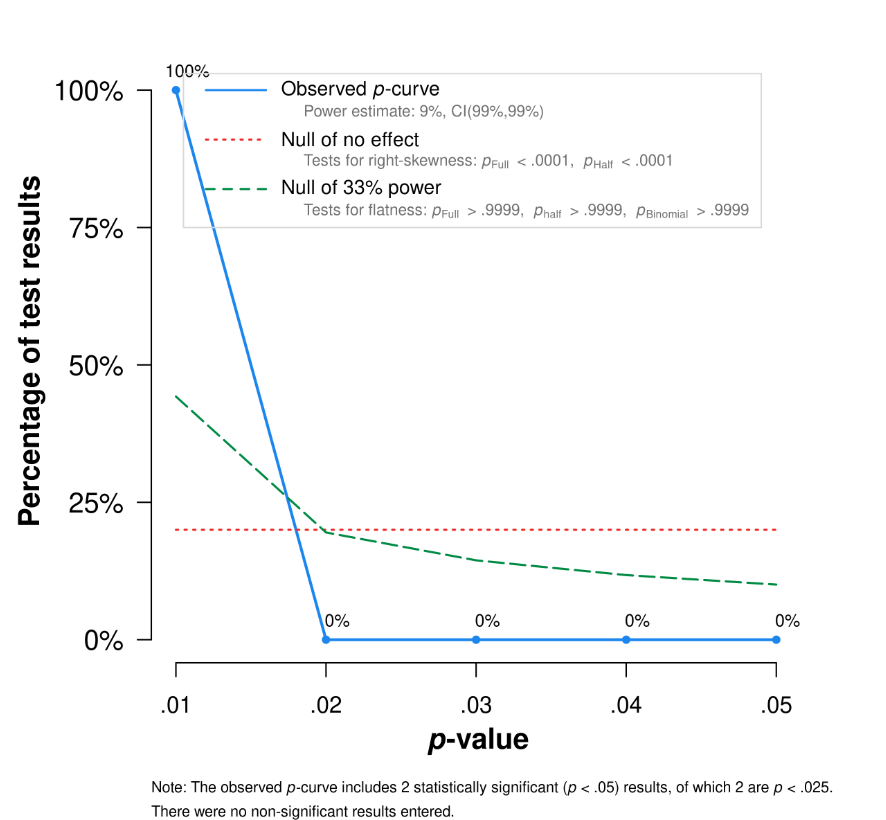

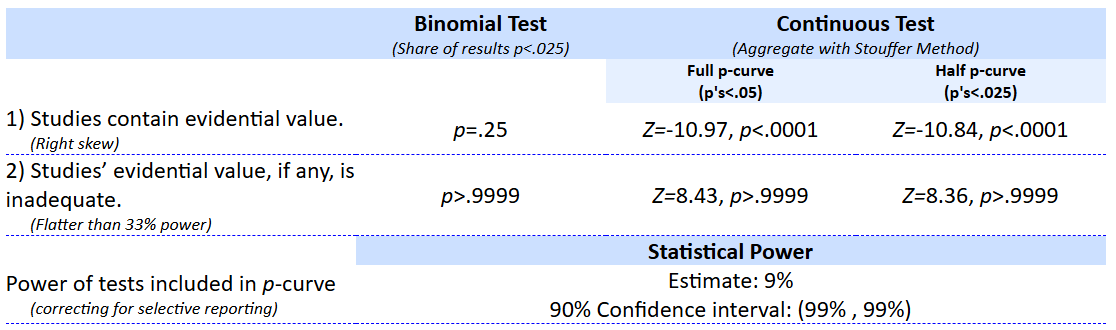


# **Figure 35.** P-curve analysis for self-reported TV-viewing.

1. All-cause mortality


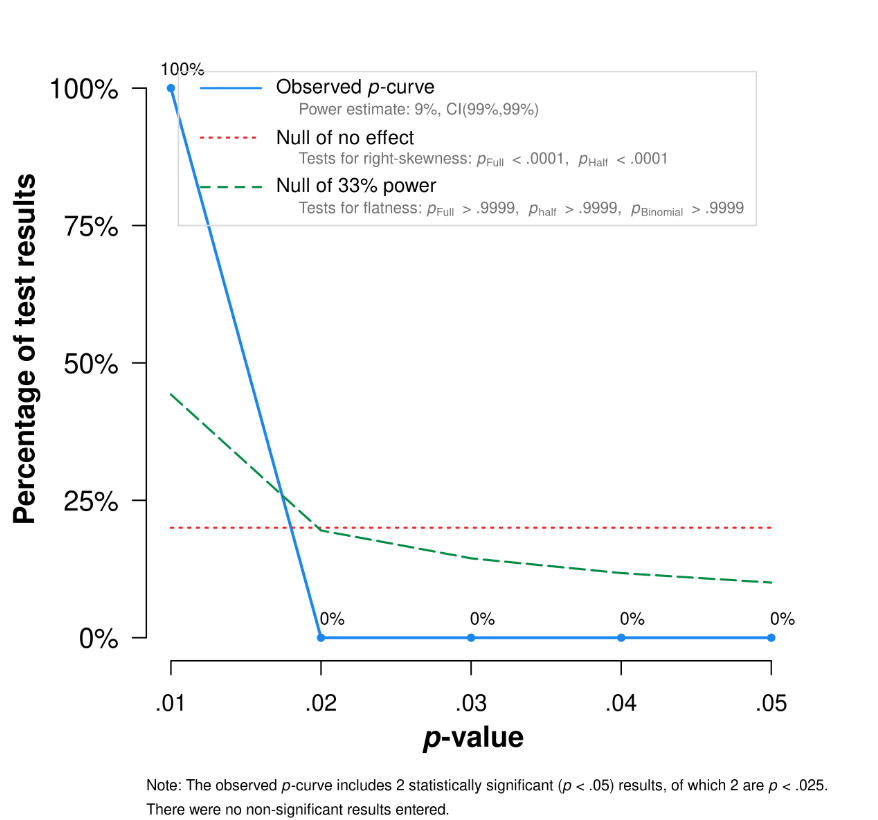

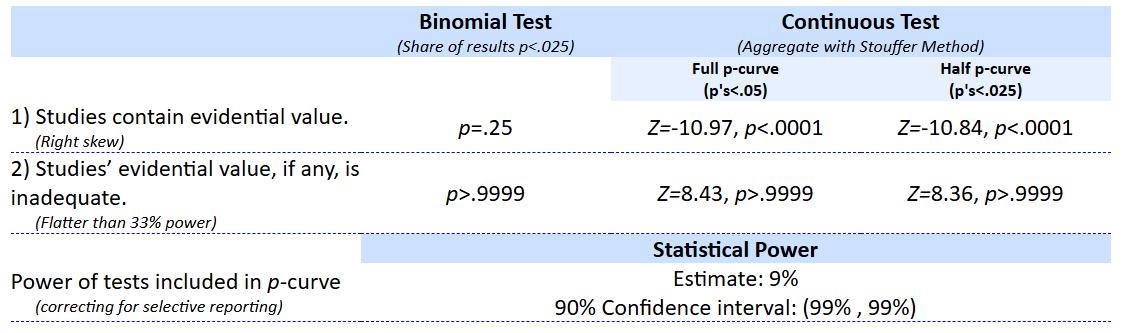


1. Cardiovascular disease mortality


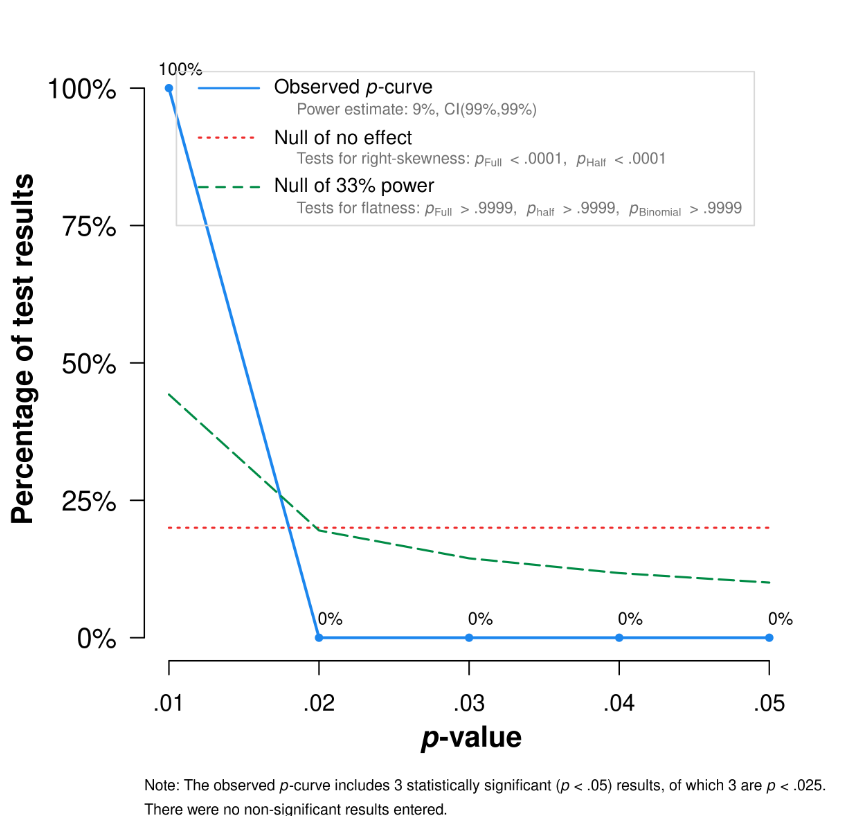

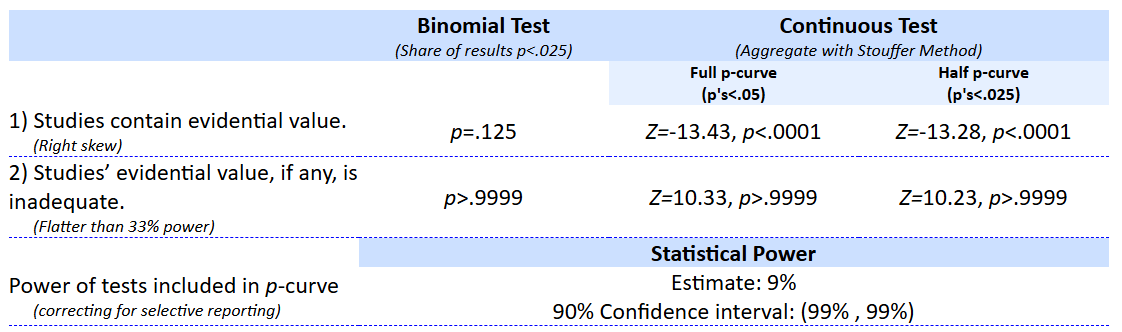


# **Figure 36.** P-curve analysis for pooled studies.

1. All-cause mortality


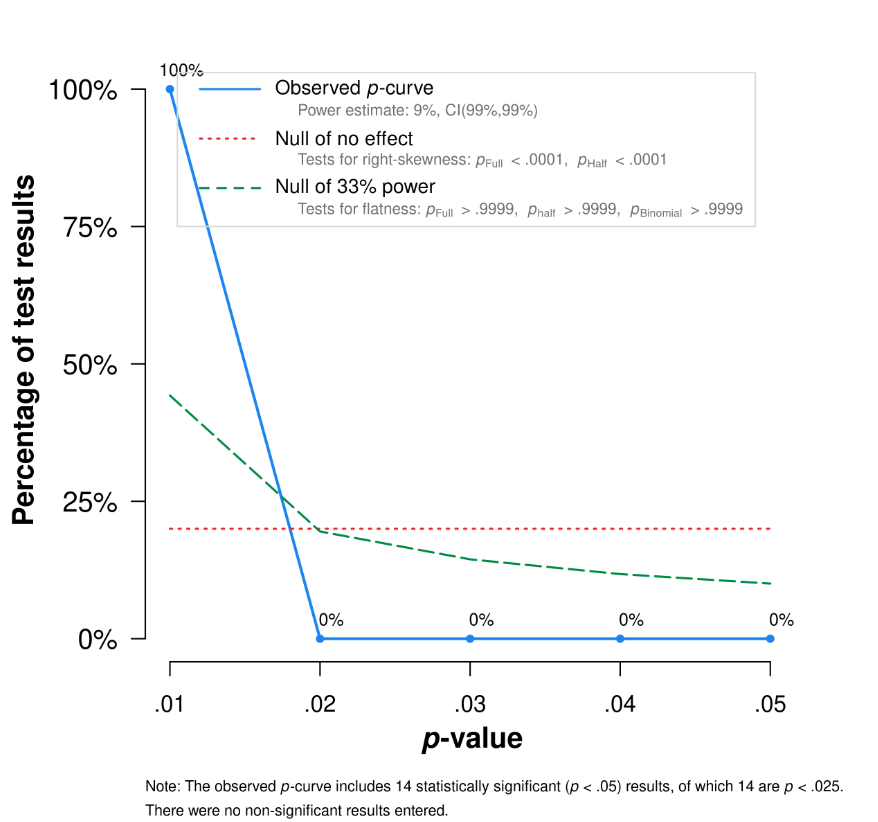

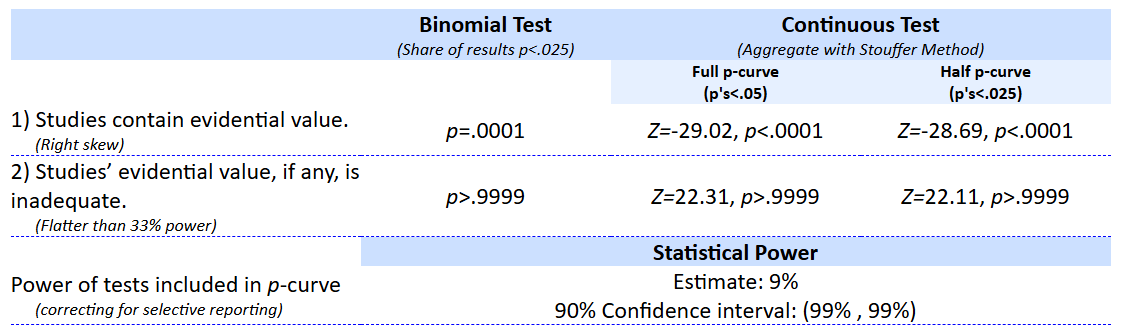


1. Cardiovascular disease mortality


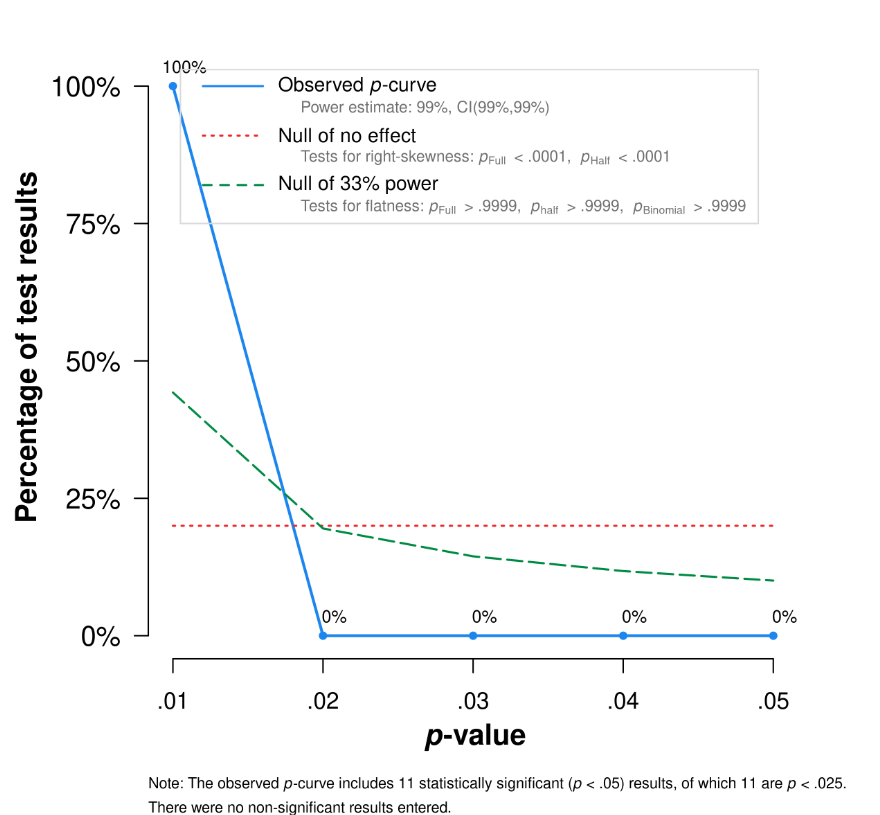

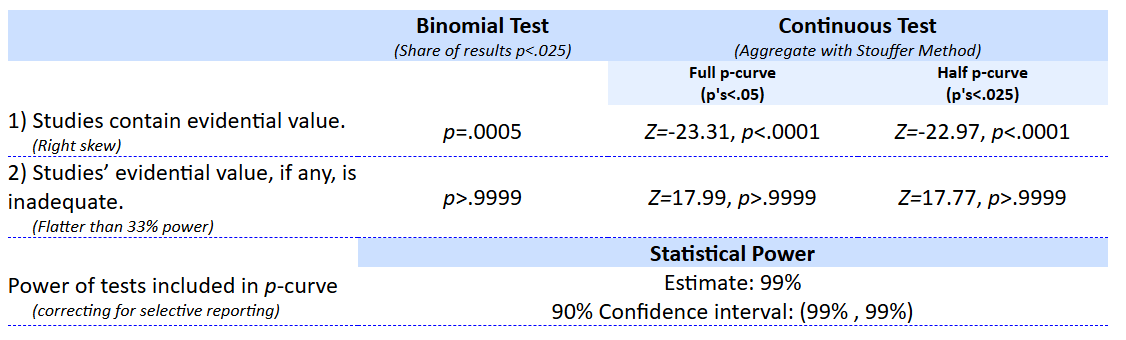


1. Cancer mortality


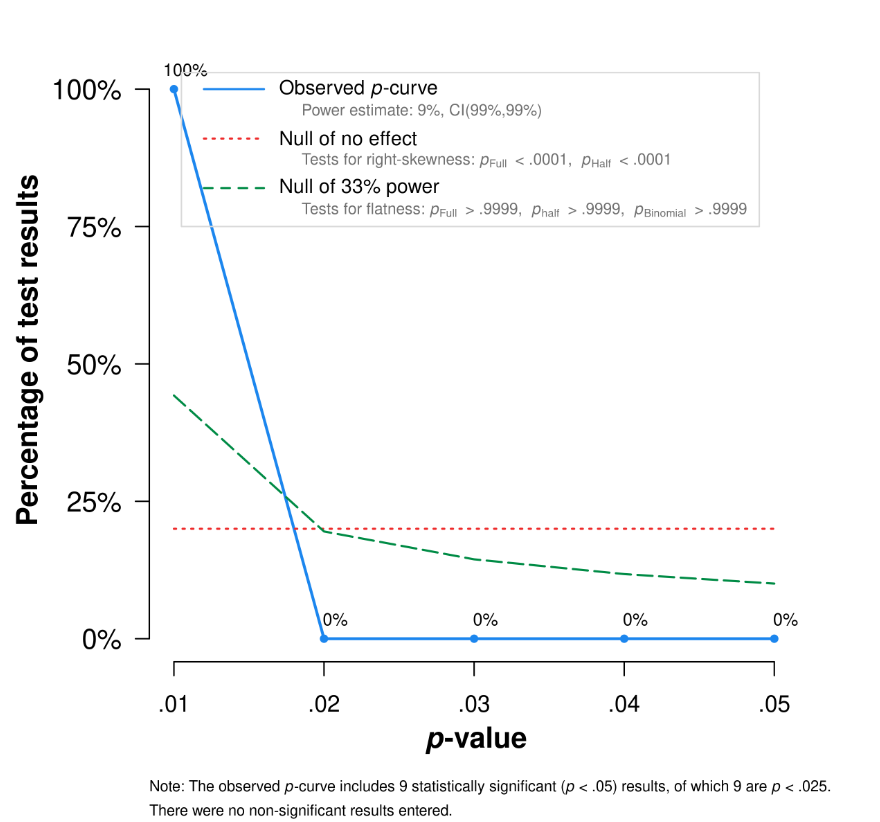


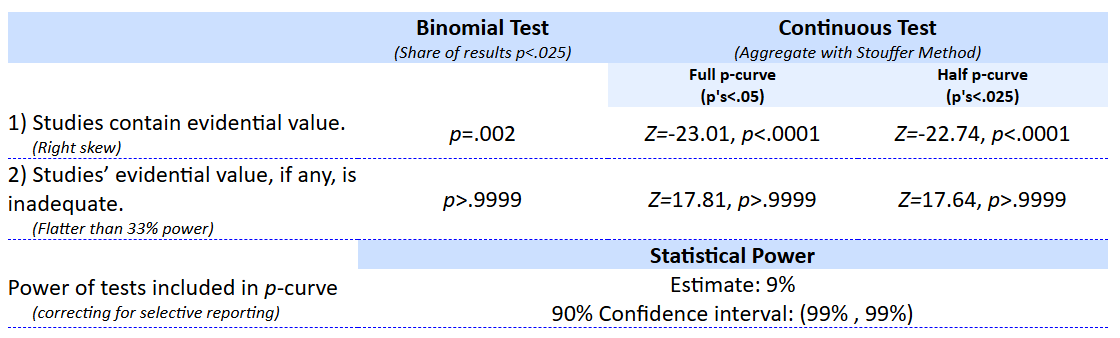


# **Figure 37.** P-curve analysis for device-measured Cardiorespiratory fitness.

1. All-cause mortality


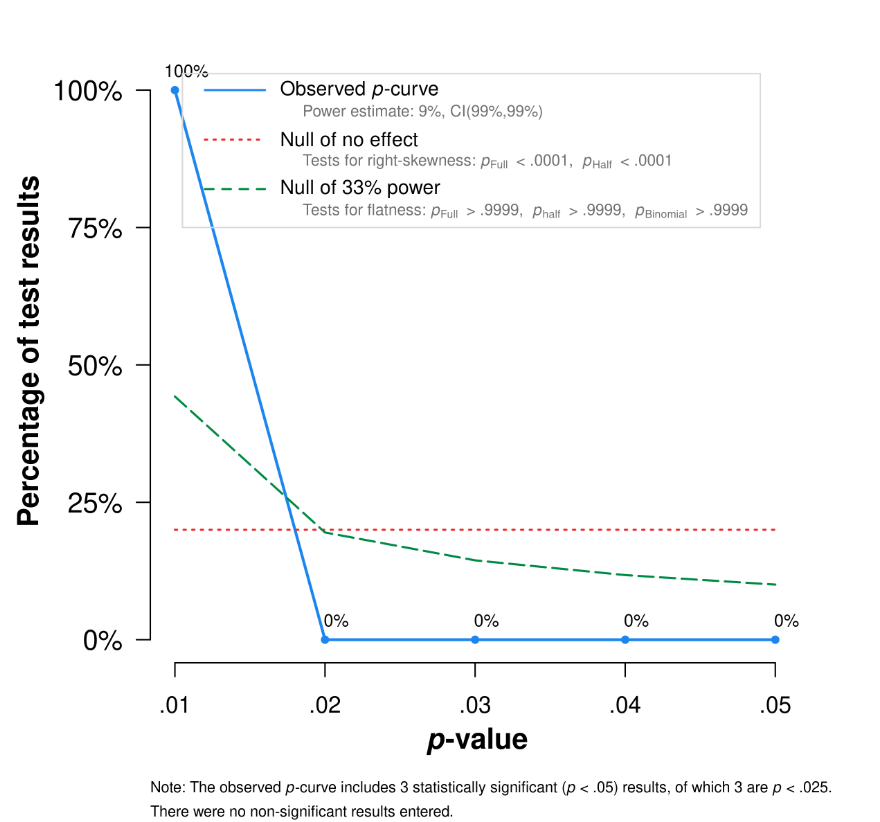

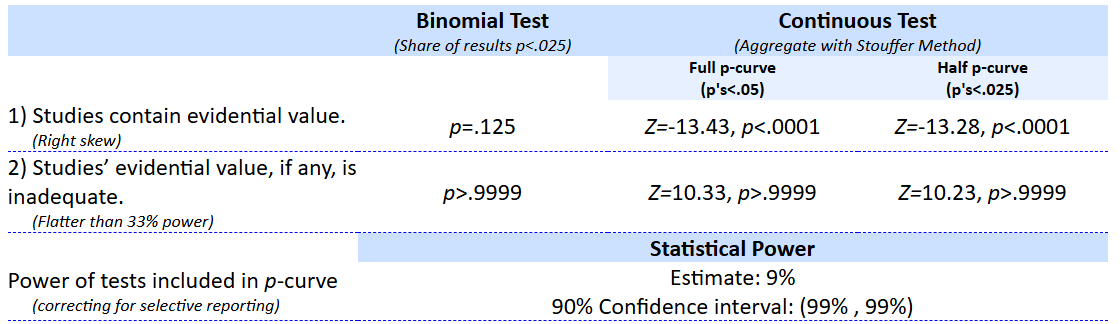


1. Cardiovascular disease mortality


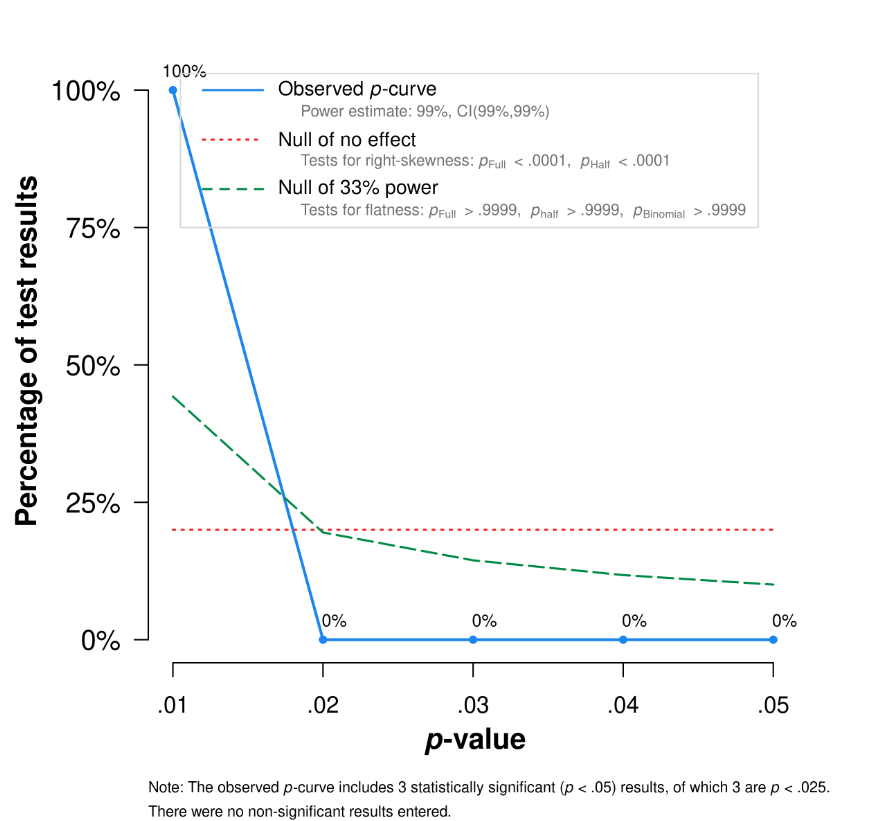

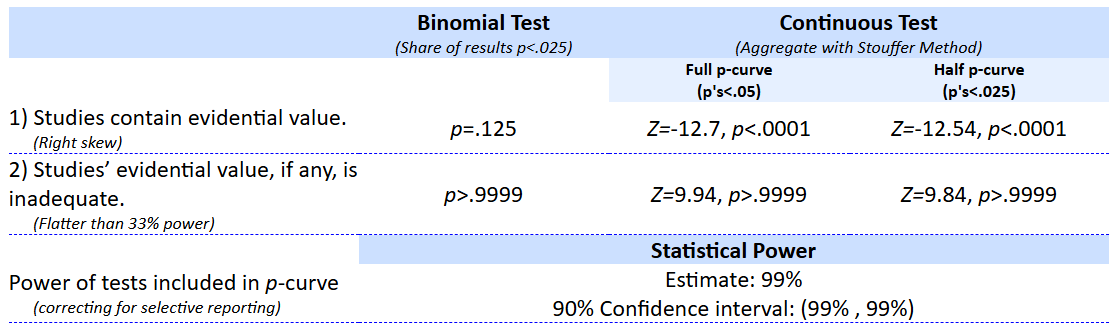


1. Cancer mortality


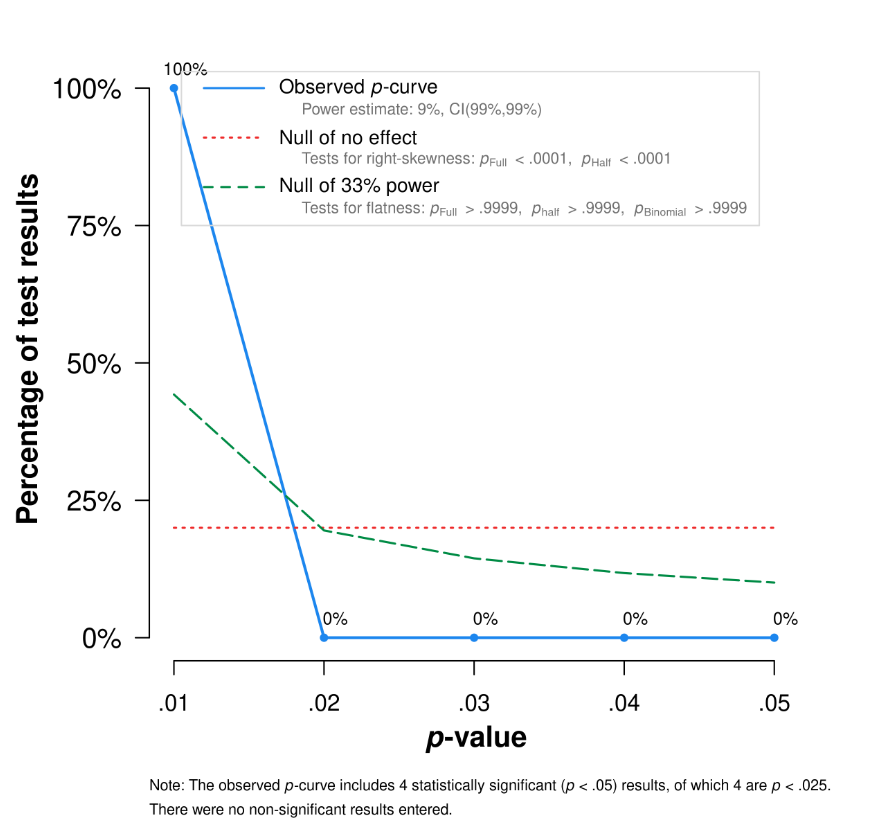

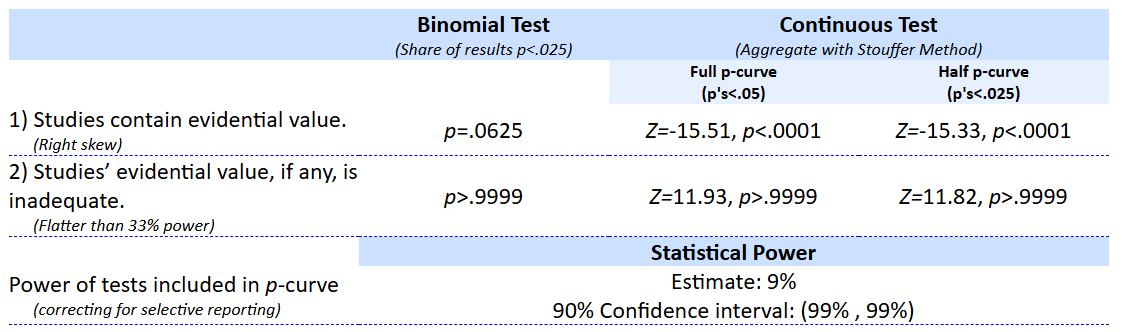


# **Figure 38.** Sensitivity analysis for self-reported LTPA and all-cause mortality.

1. Forest plot


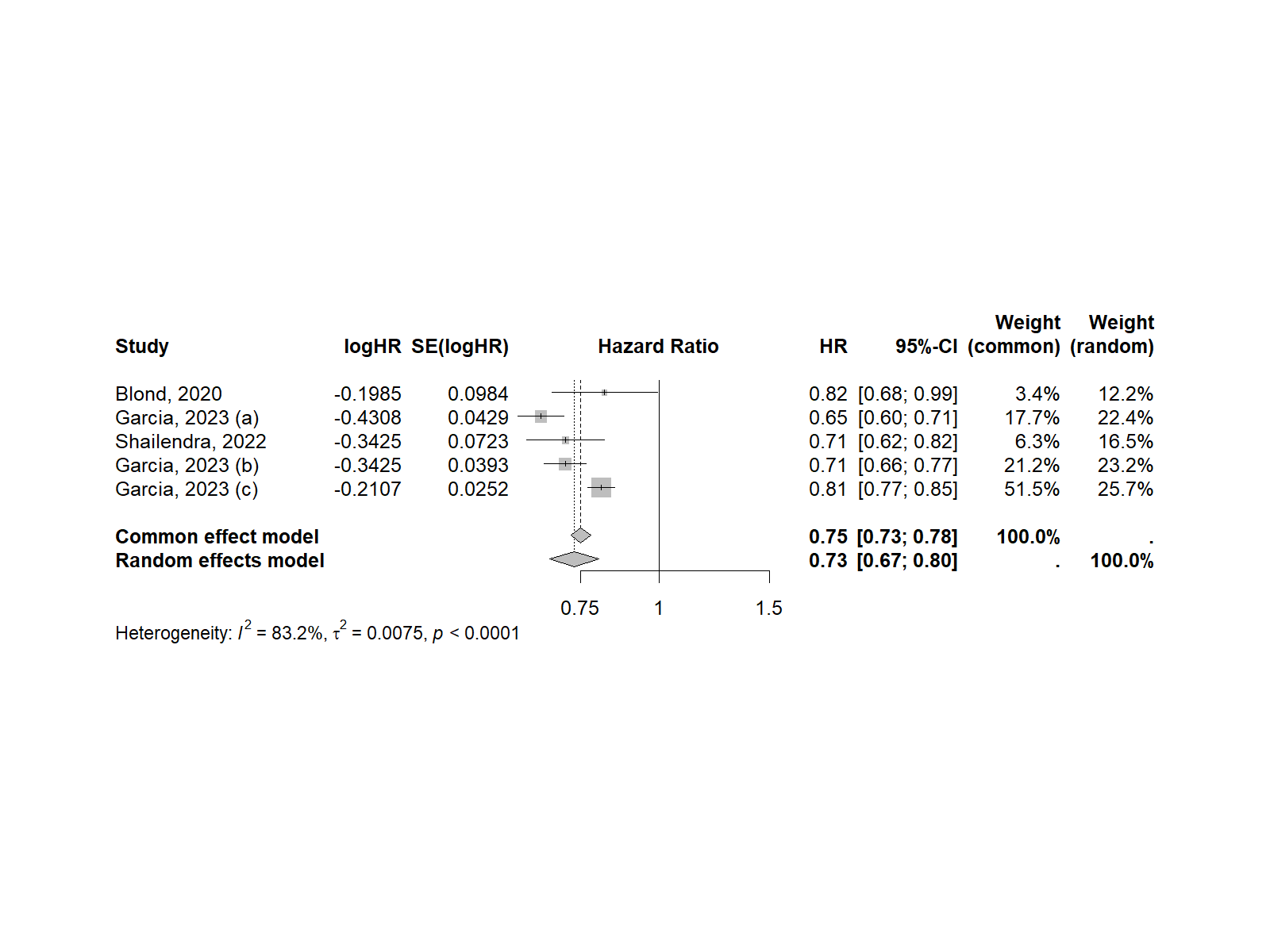


1. Influence diagnostics


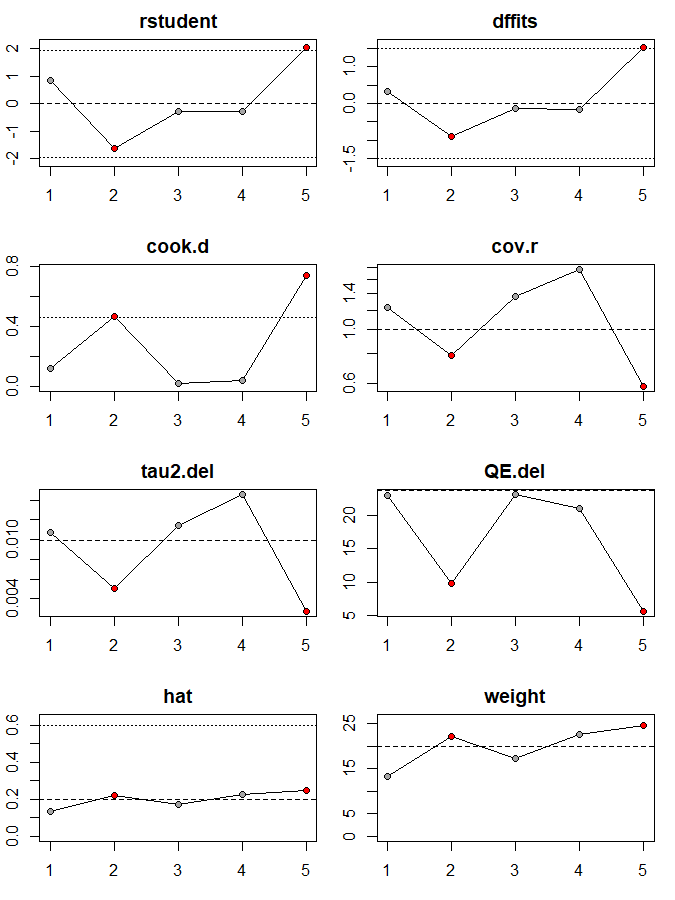


1. Forest plot (sensitivity analysis)


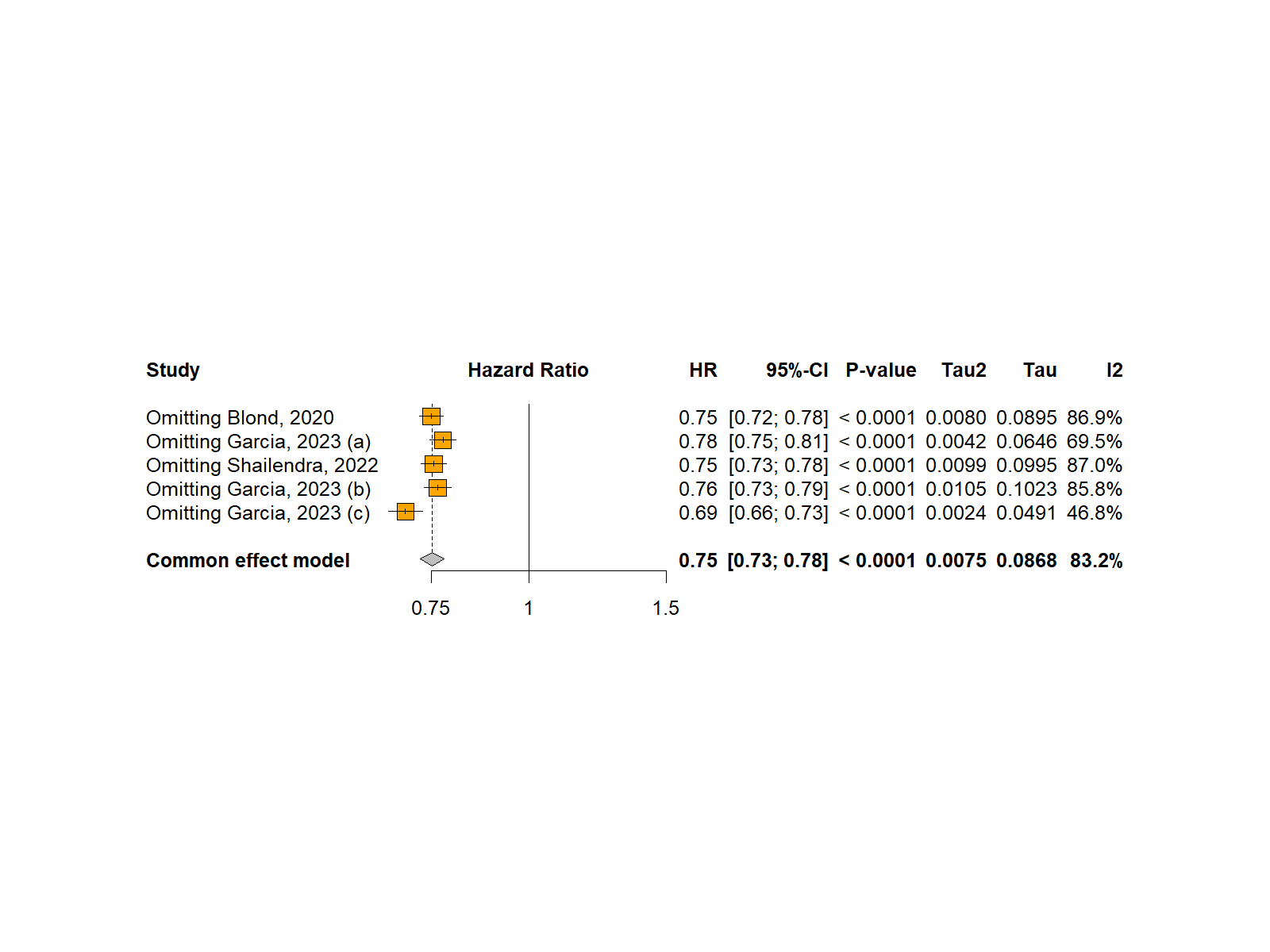


# **Figure 39.** Sensitivity analysis for device-measured TPA and all-cause mortality.

1. Forest plot


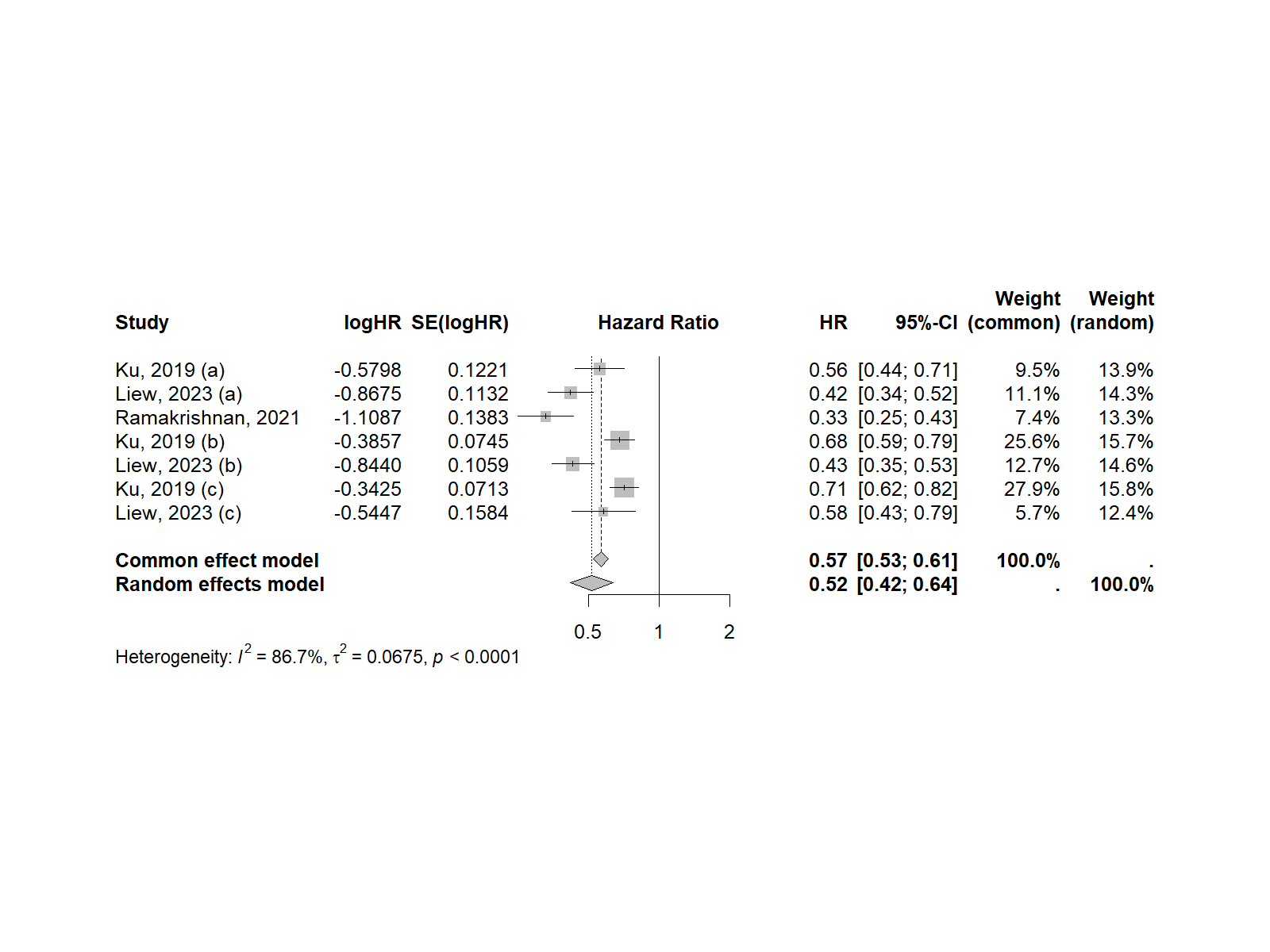


1. Influence diagnostics


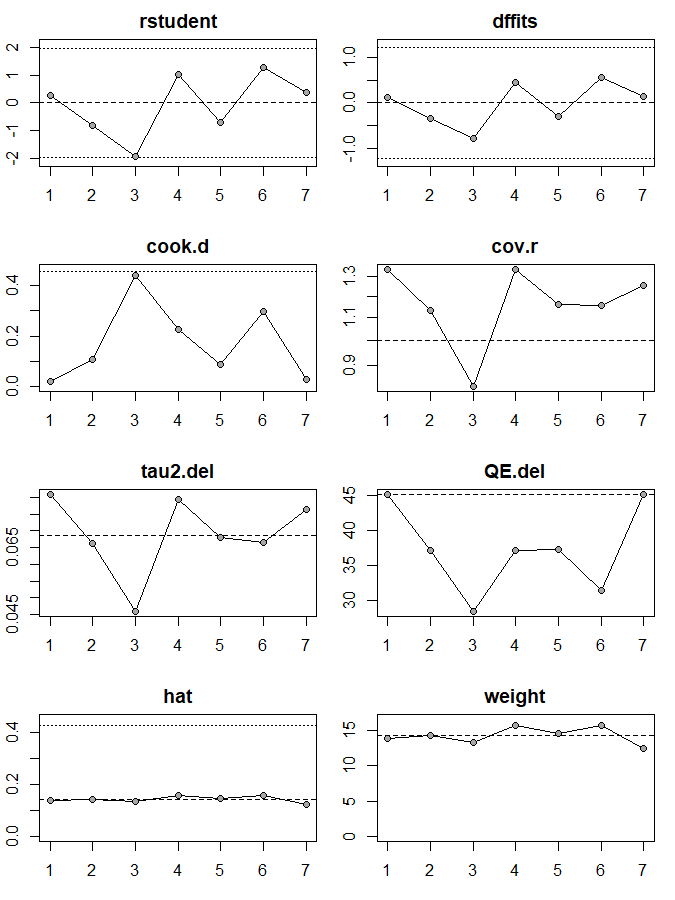


1. Forest plot (sensitivity analysis)


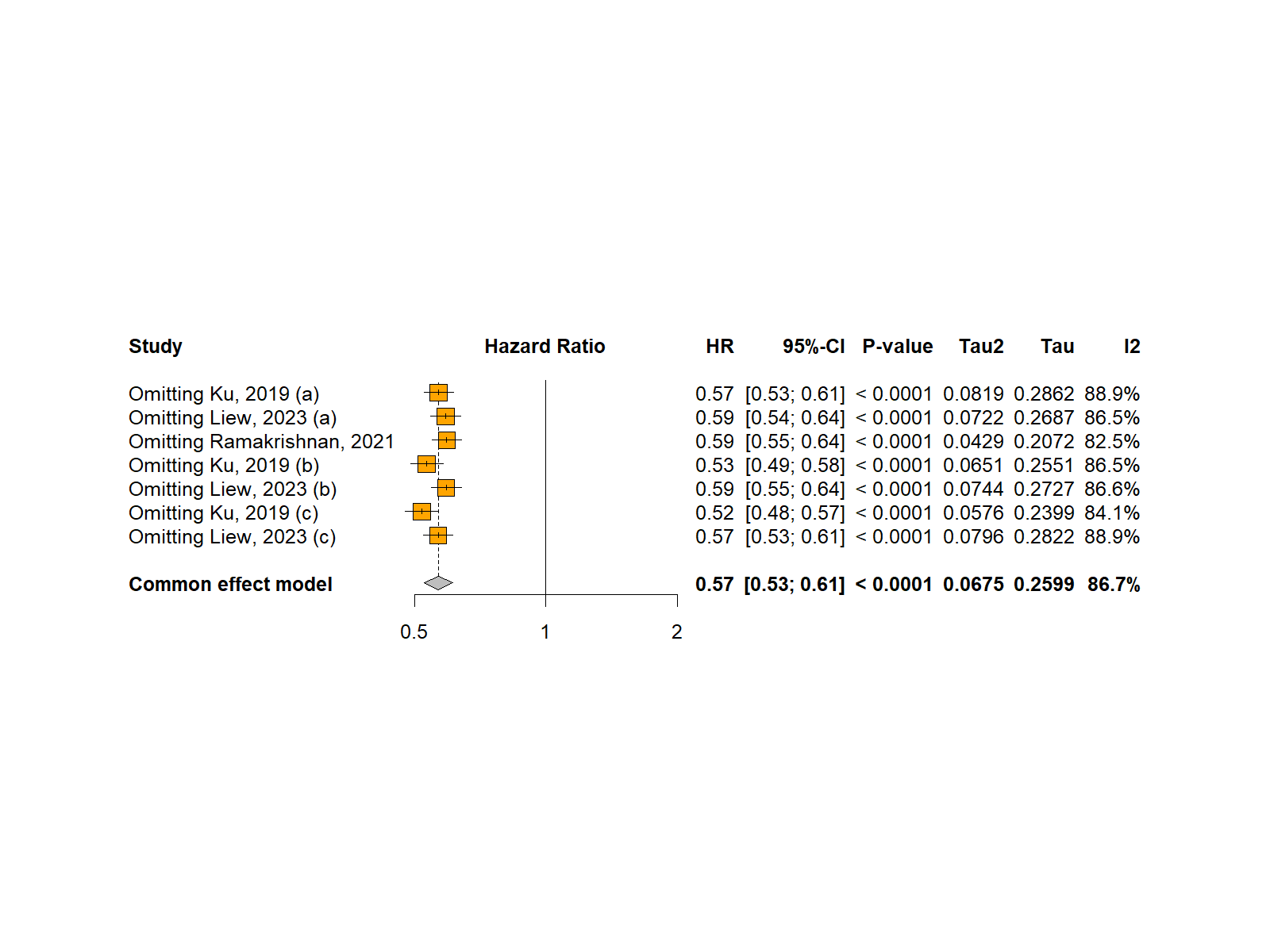


# **Figure 40.** Sensitivity analysis for self-reported SB and all-cause mortality.

1. Forest plot


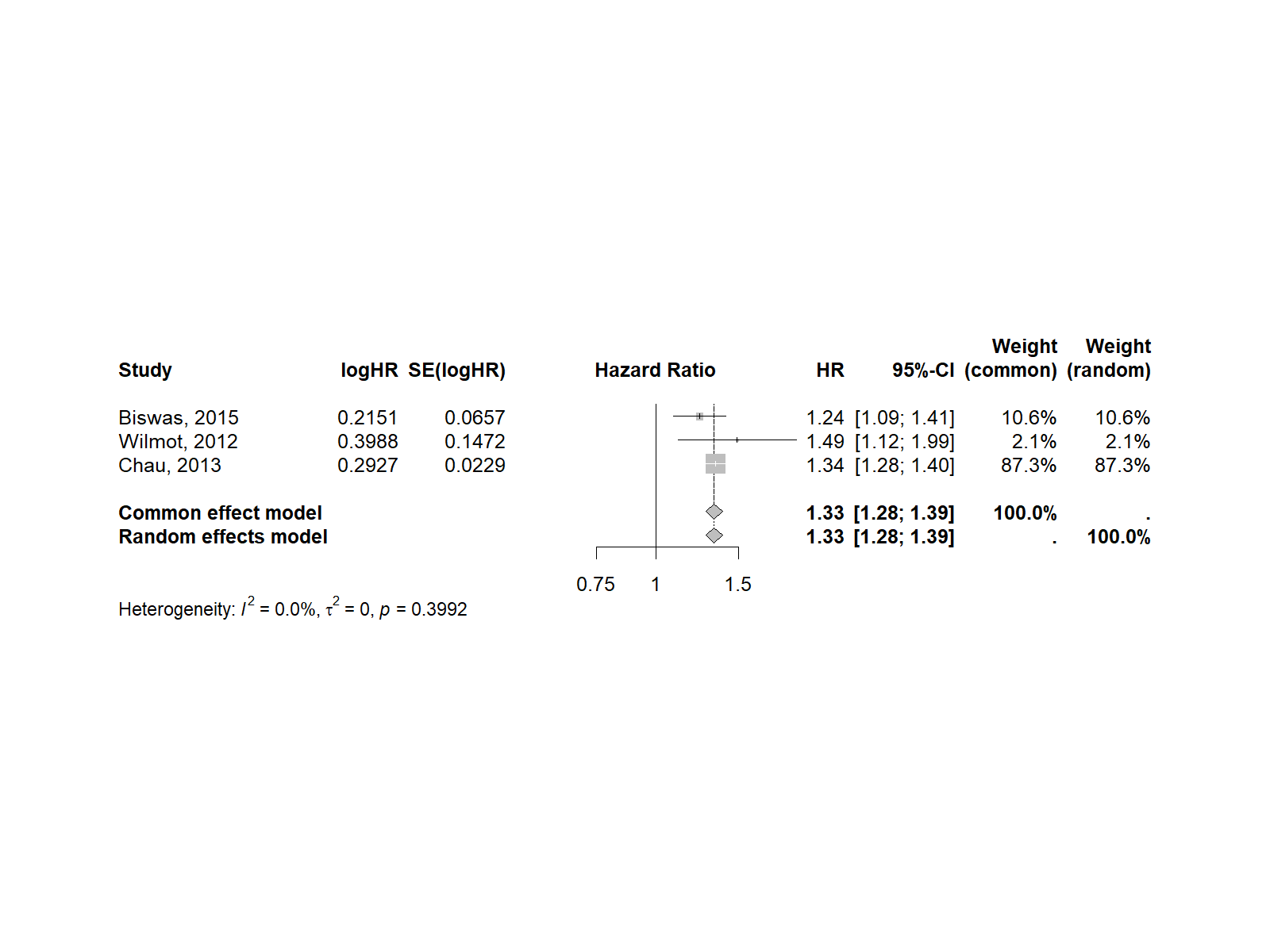


1. Influence diagnostics


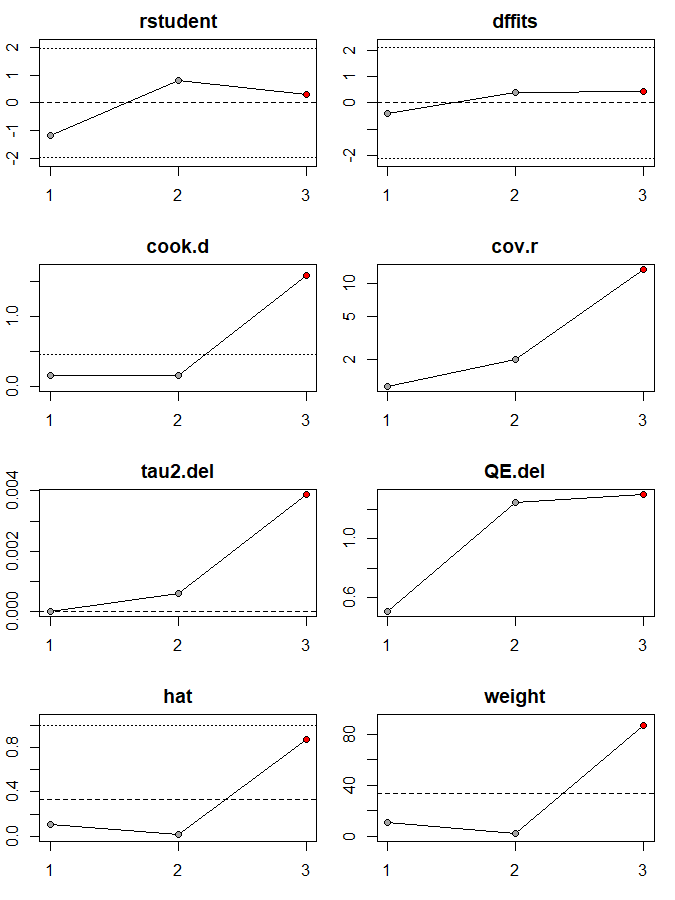


1. Forest plot (sensitivity analysis)


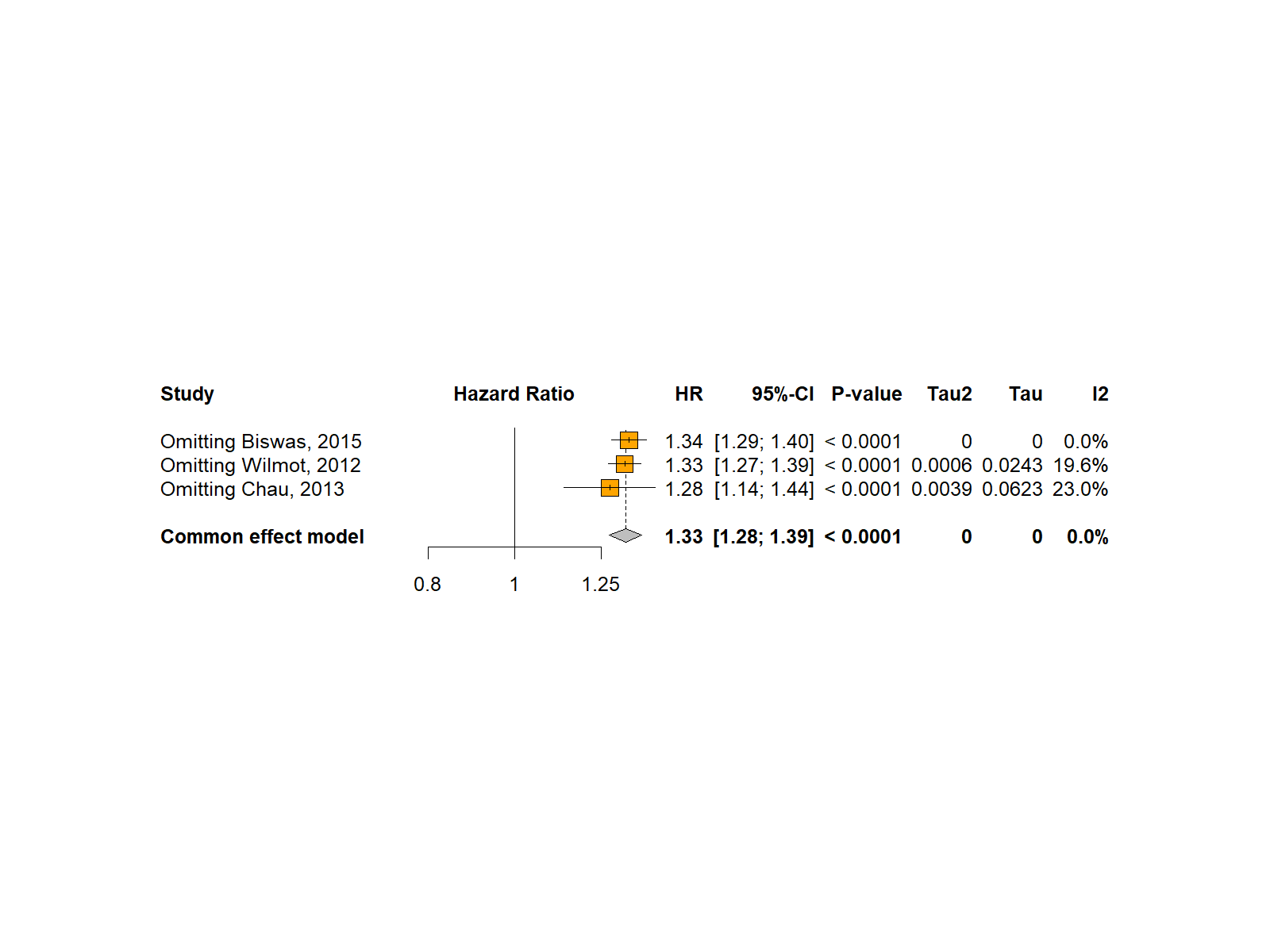


# **Figure 41.** Sensitivity analysis for device-measured SB and all-cause mortality.

1. Forest plot


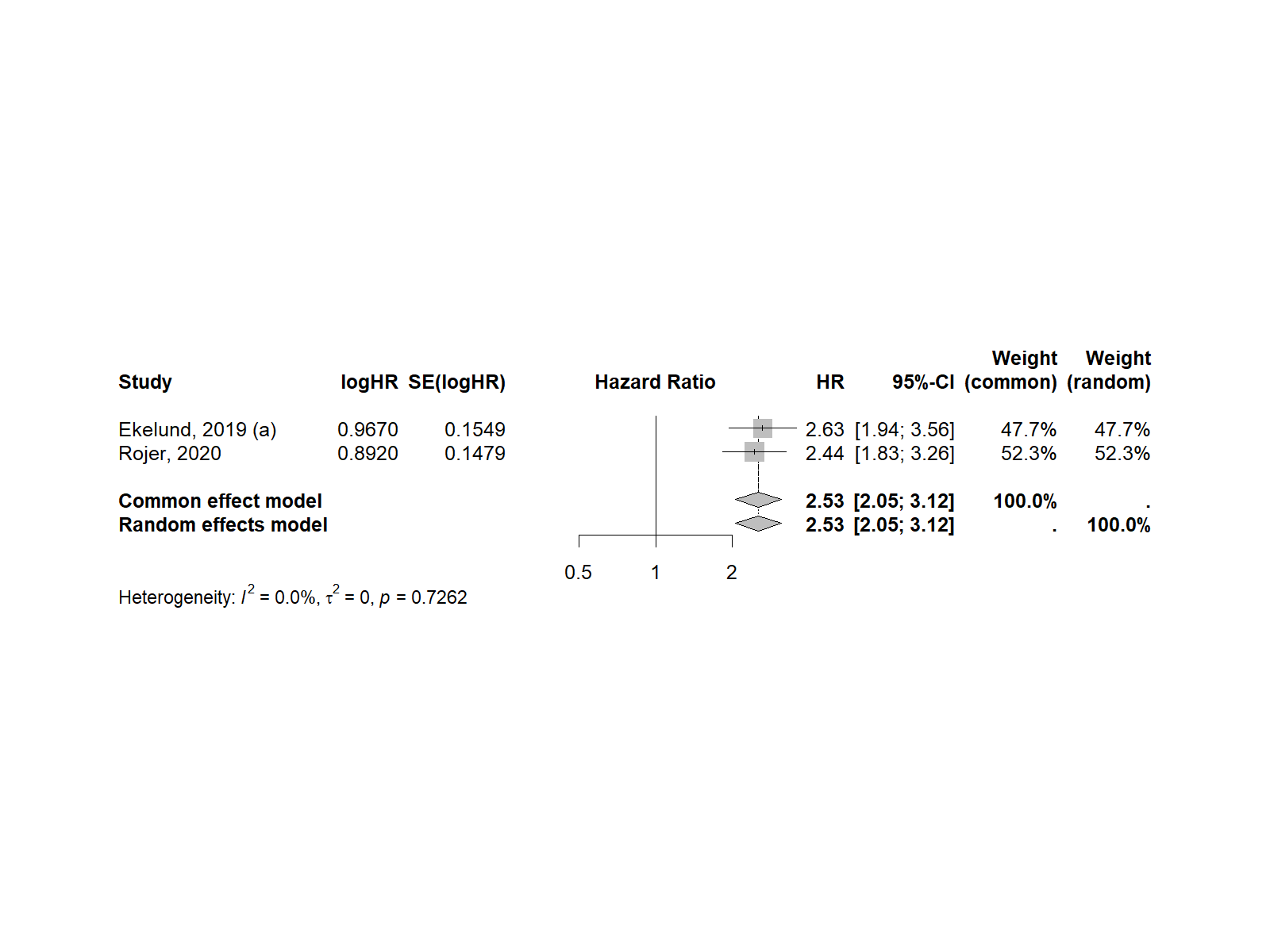


1. Influence diagnostics


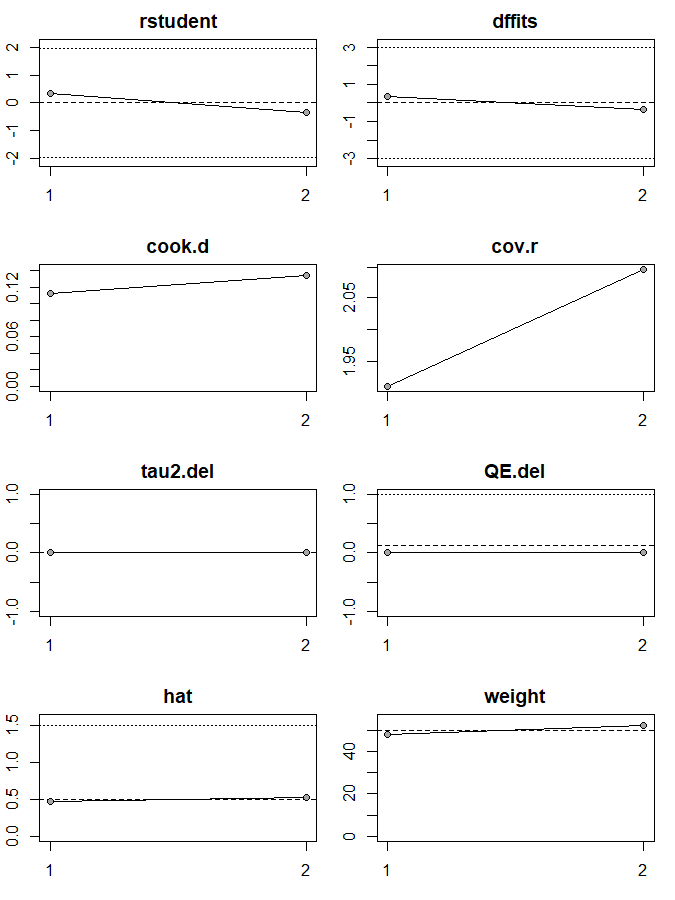


1. Forest plot (sensitivity analysis)


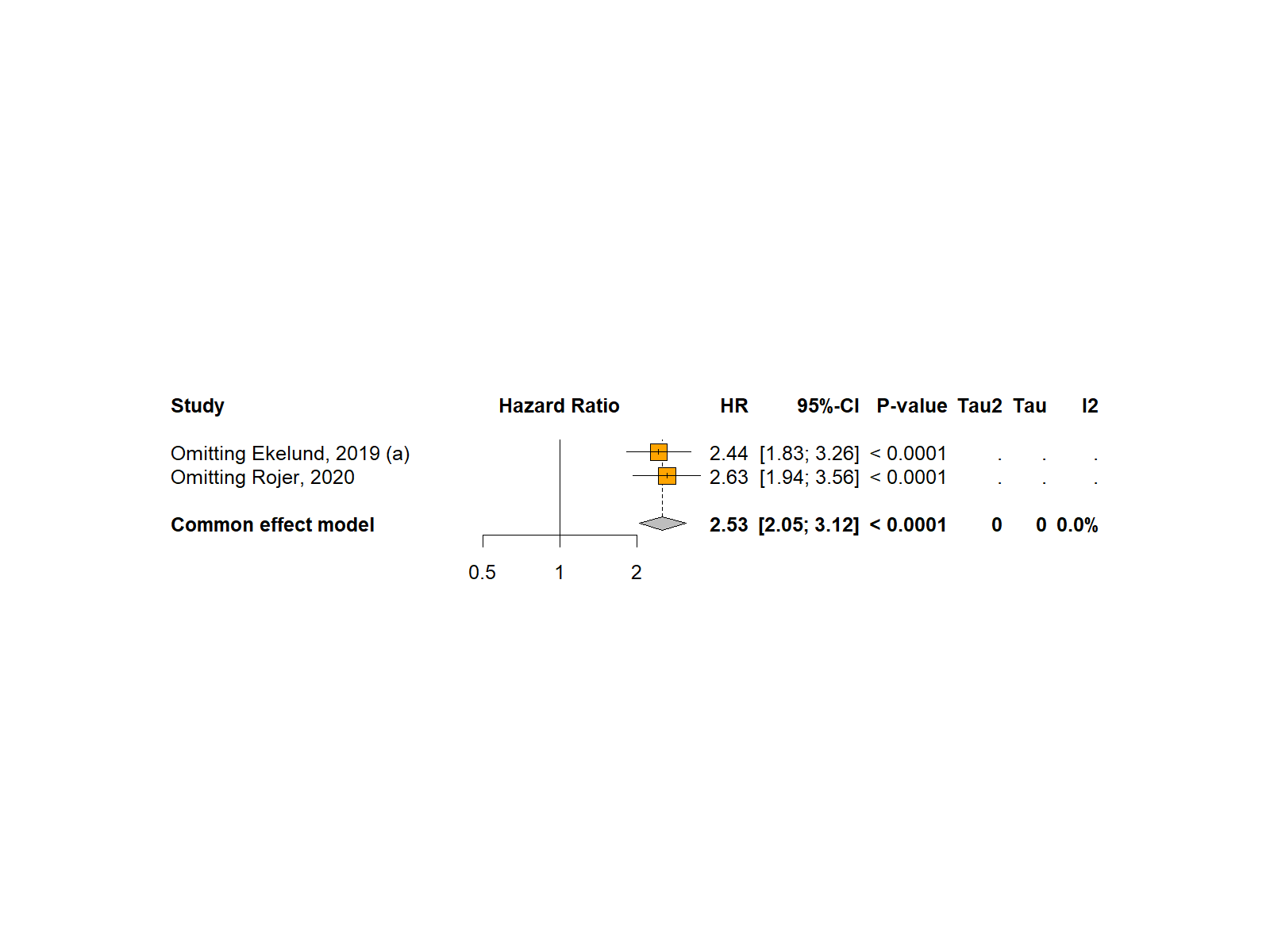


# **Figure 42.** Sensitivity analysis for PA and all-cause mortality in pooled analysis studies.

1. Forest plot


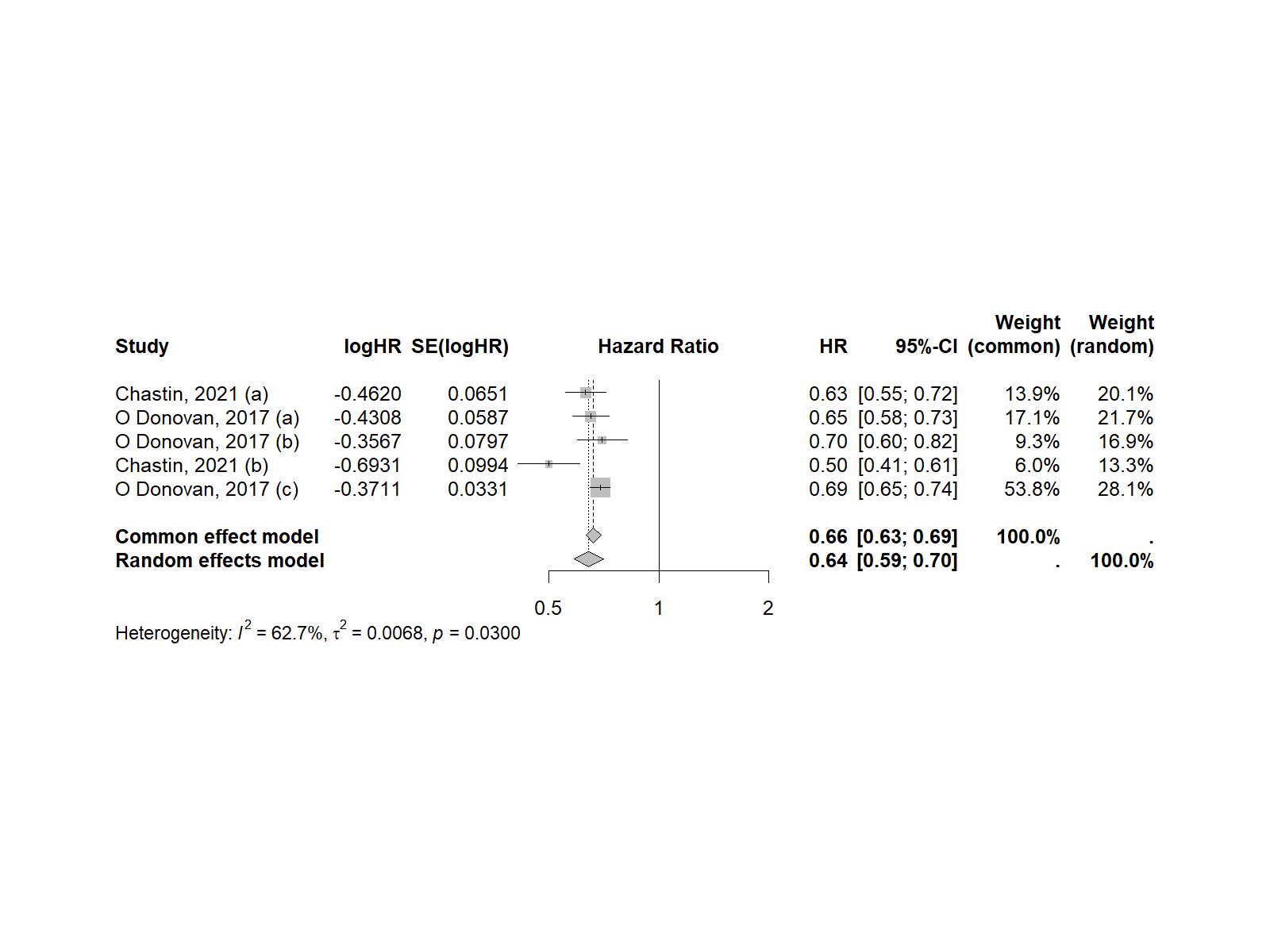


1. Influence diagnostics


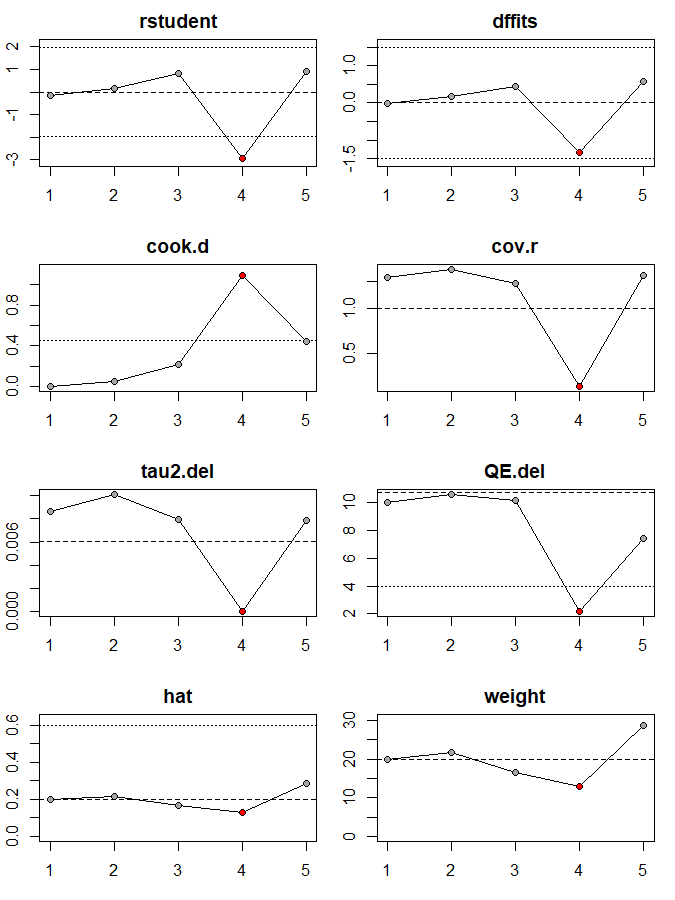


1. Forest plot (sensitivity analysis)


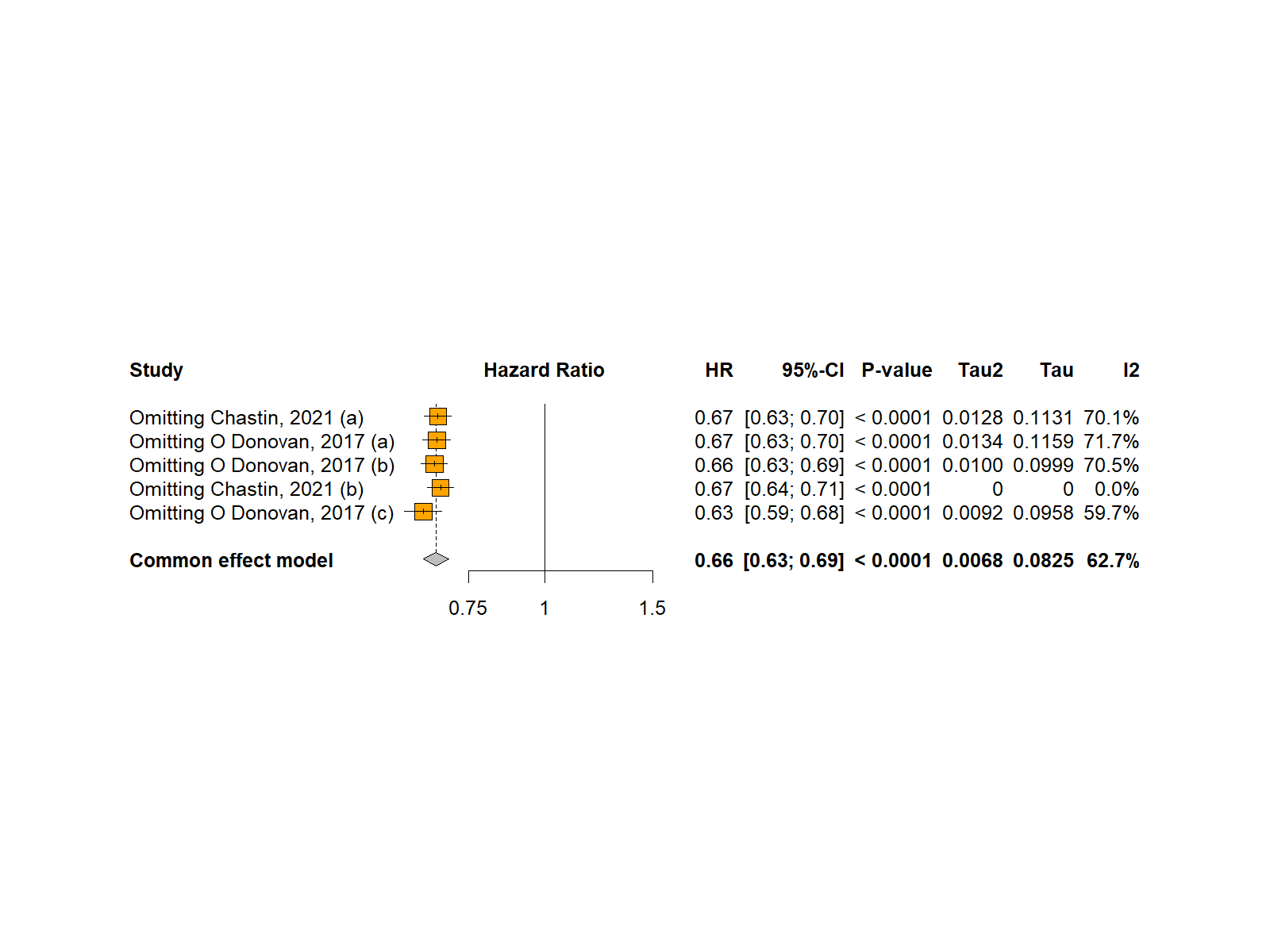


# **Figure 42.** Sensitivity analysis for PA and CVD mortality in pooled analysis studies.

1. Forest plot


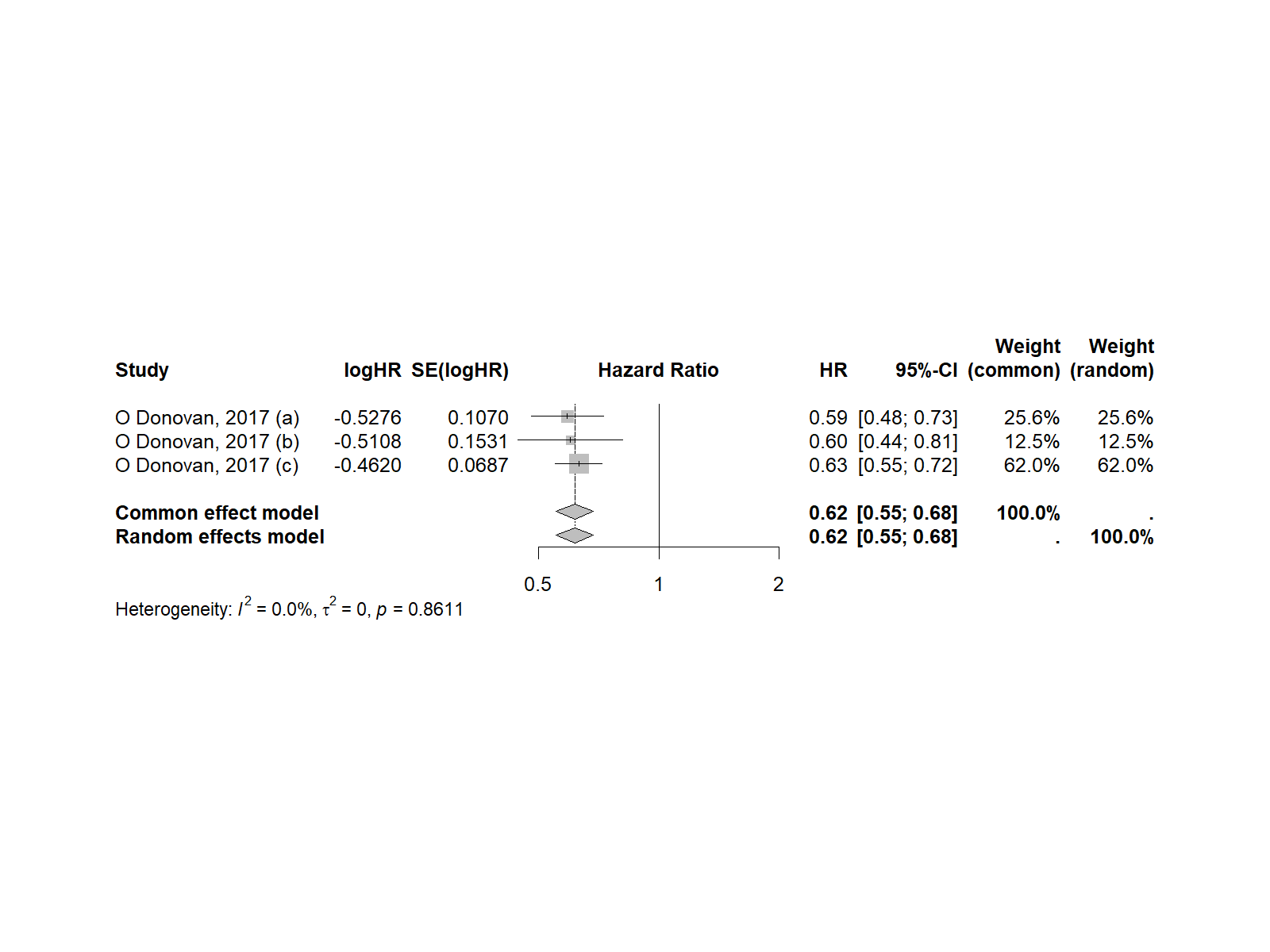


1. Influence diagnostics


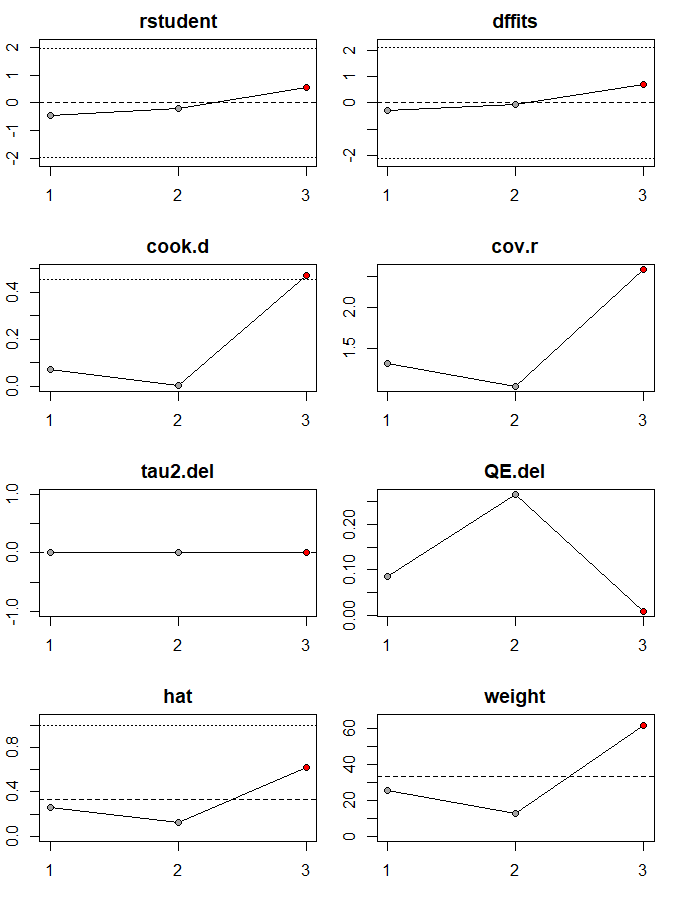


1. Forest plot (sensitivity analysis)


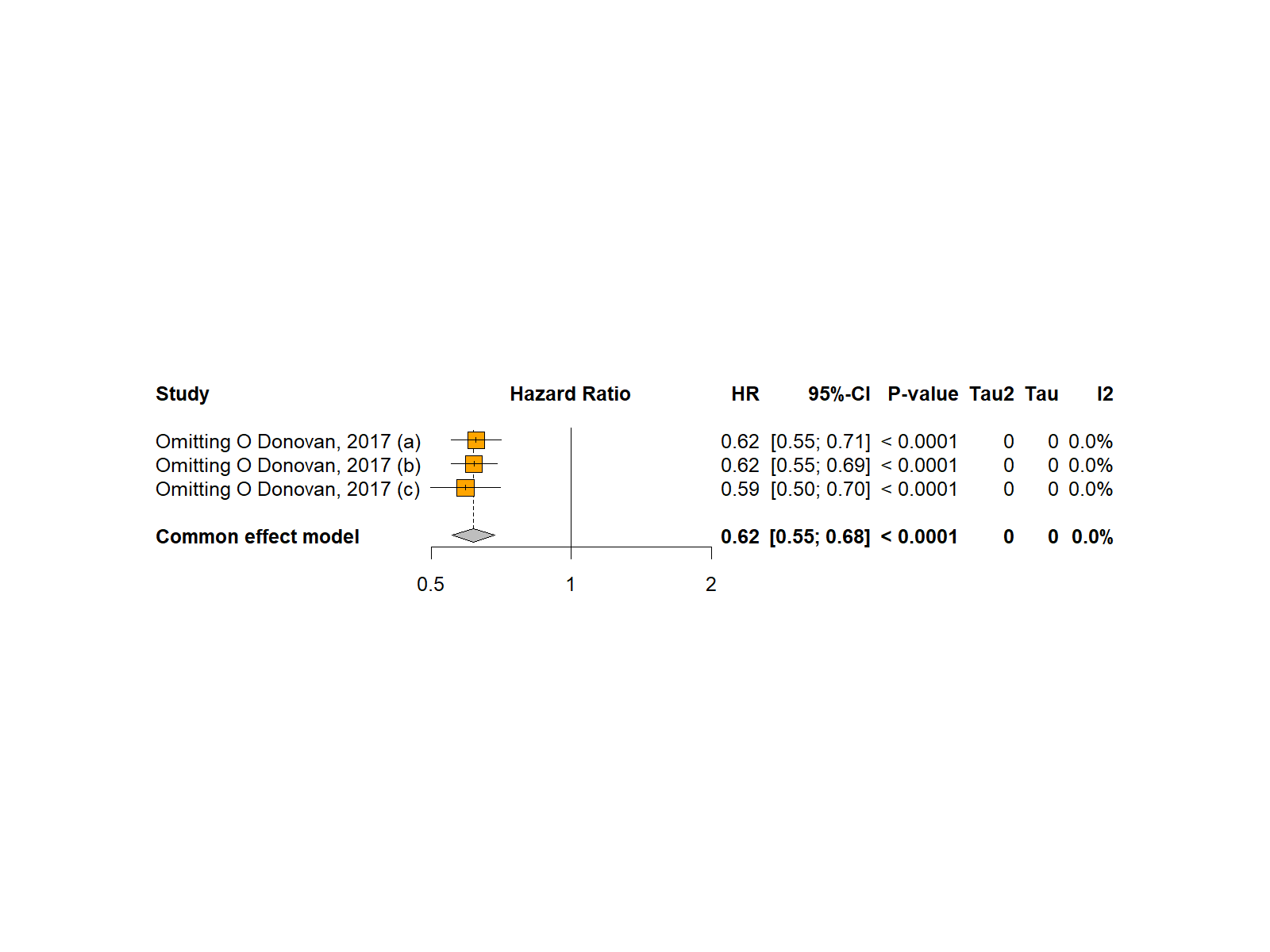


# **Figure 44.** Sensitivity analysis for PA and cancer mortality in pooled analysis studies.

1. Forest plot


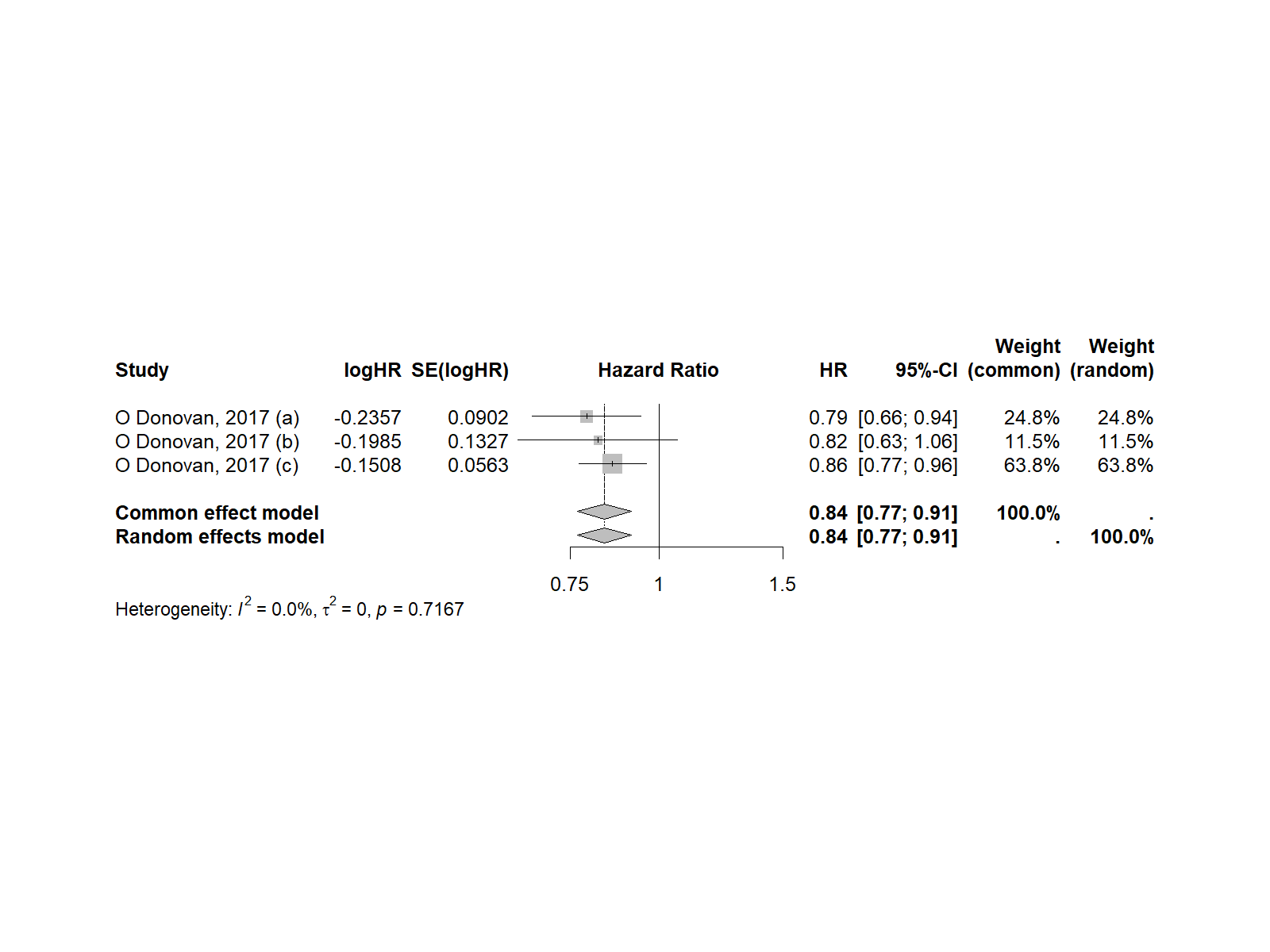


1. Influence diagnostics


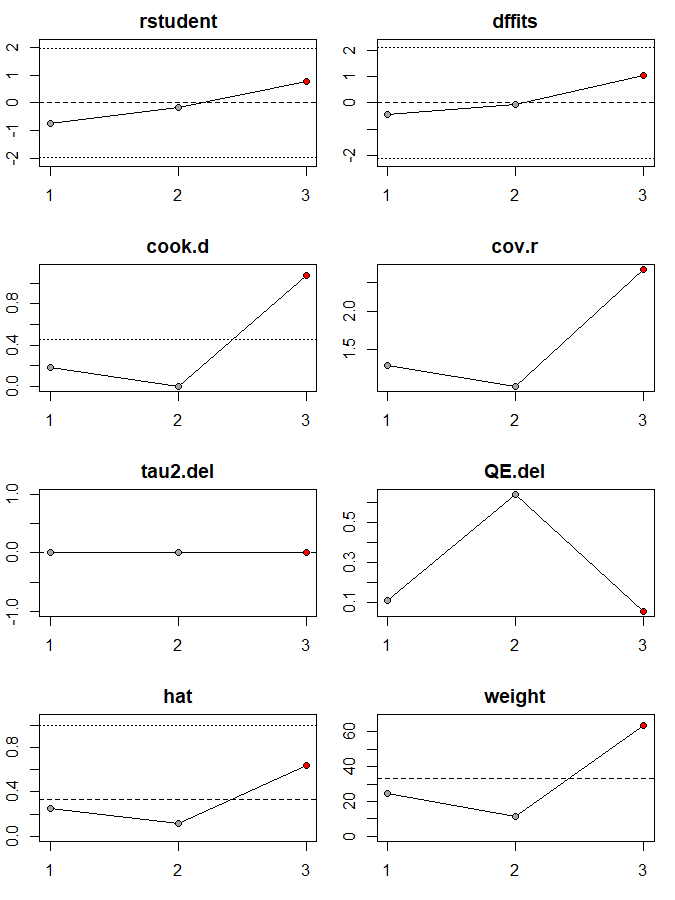


1. Forest plot (sensitivity analysis)


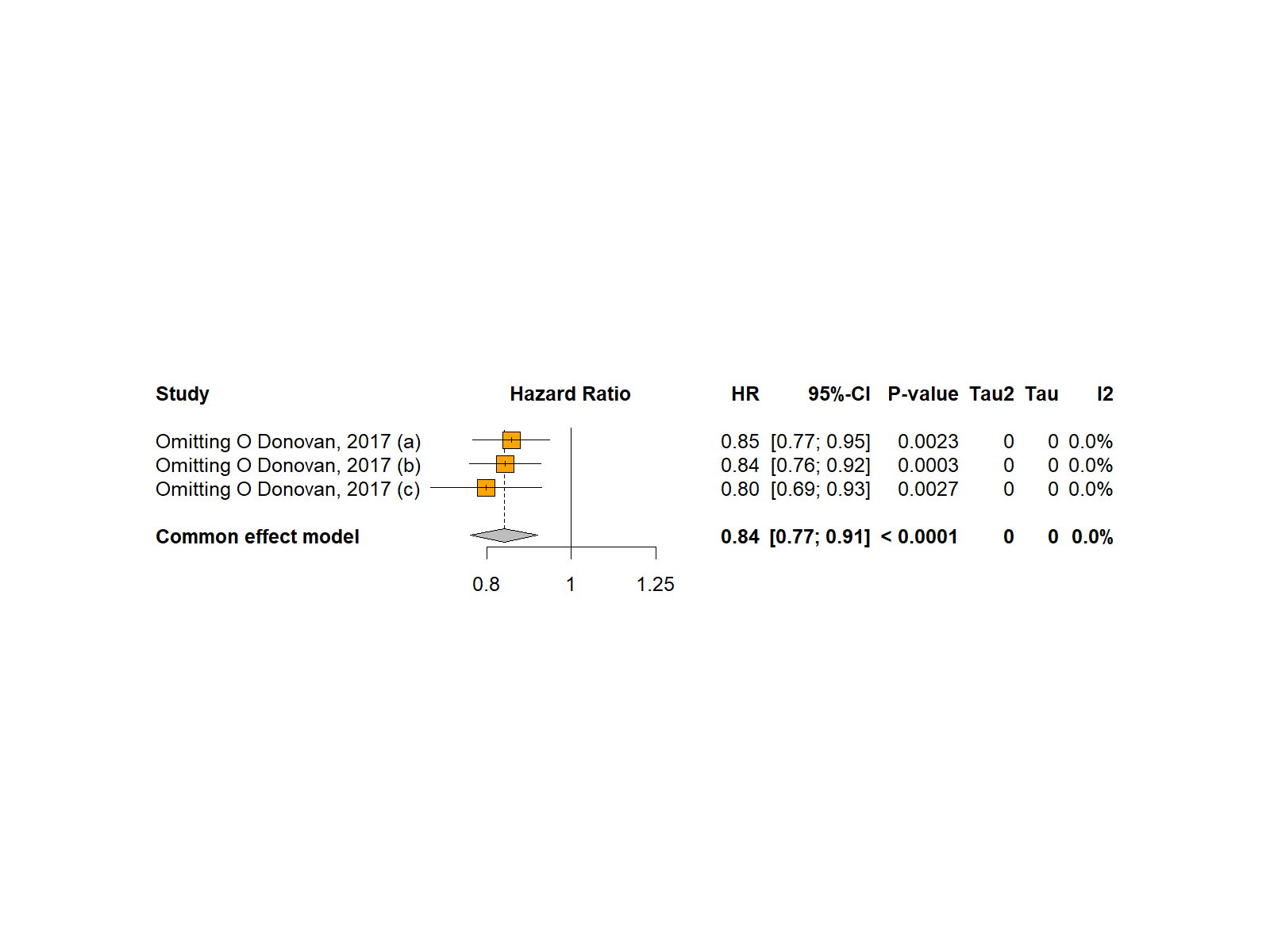


# **Figure 45.** Sensitivity analysis for resistance training and all-cause mortality.

1. Forest plot


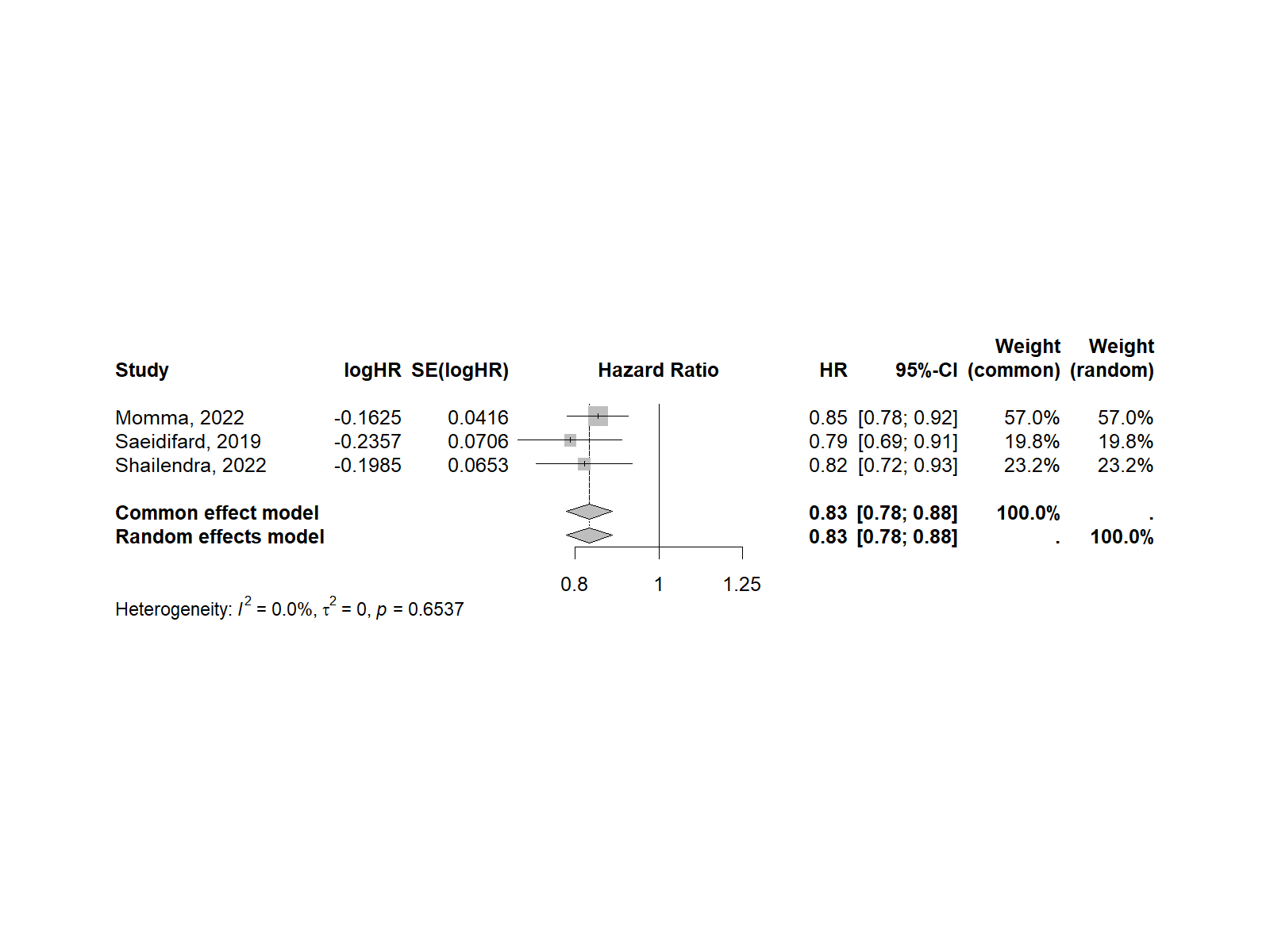


1. Influence diagnostics


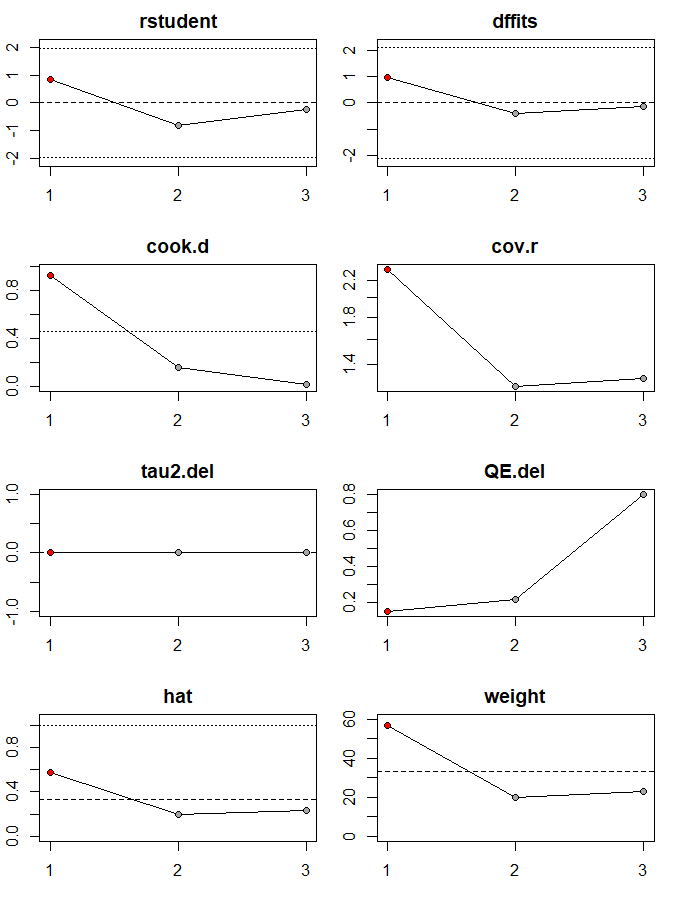


1. Forest plot (sensitivity analysis)


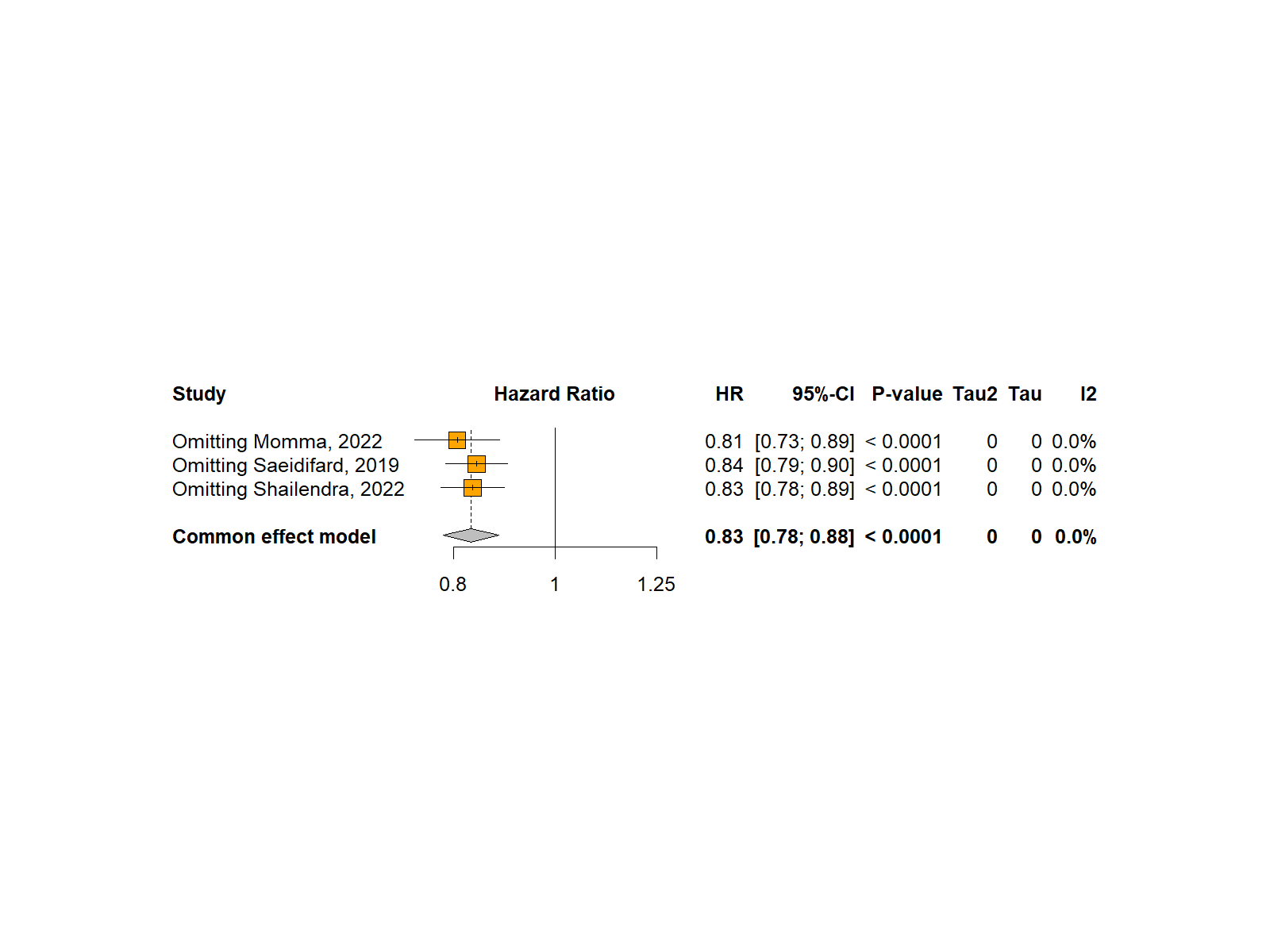


# **Figure 46.** Sensitivity analysis for resistance training and cancer mortality.

1. Forest plot


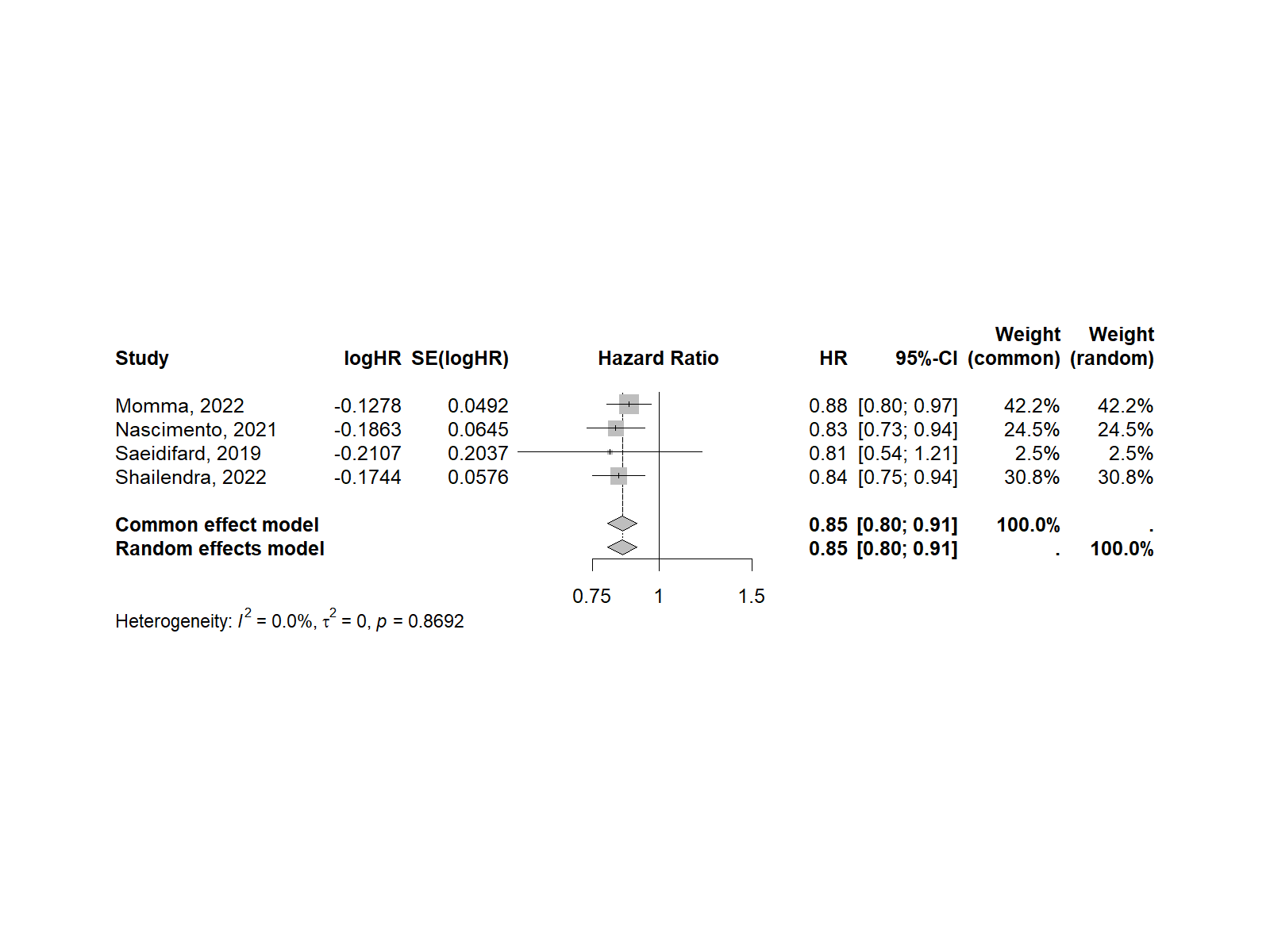


1. Influence diagnostics


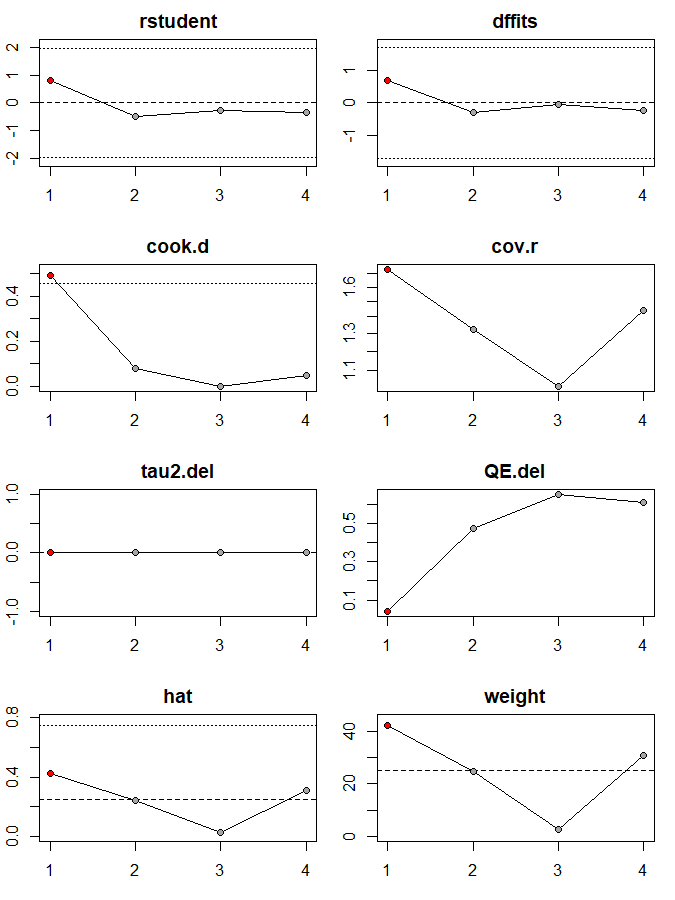


1. Forest plot (sensitivity analysis)


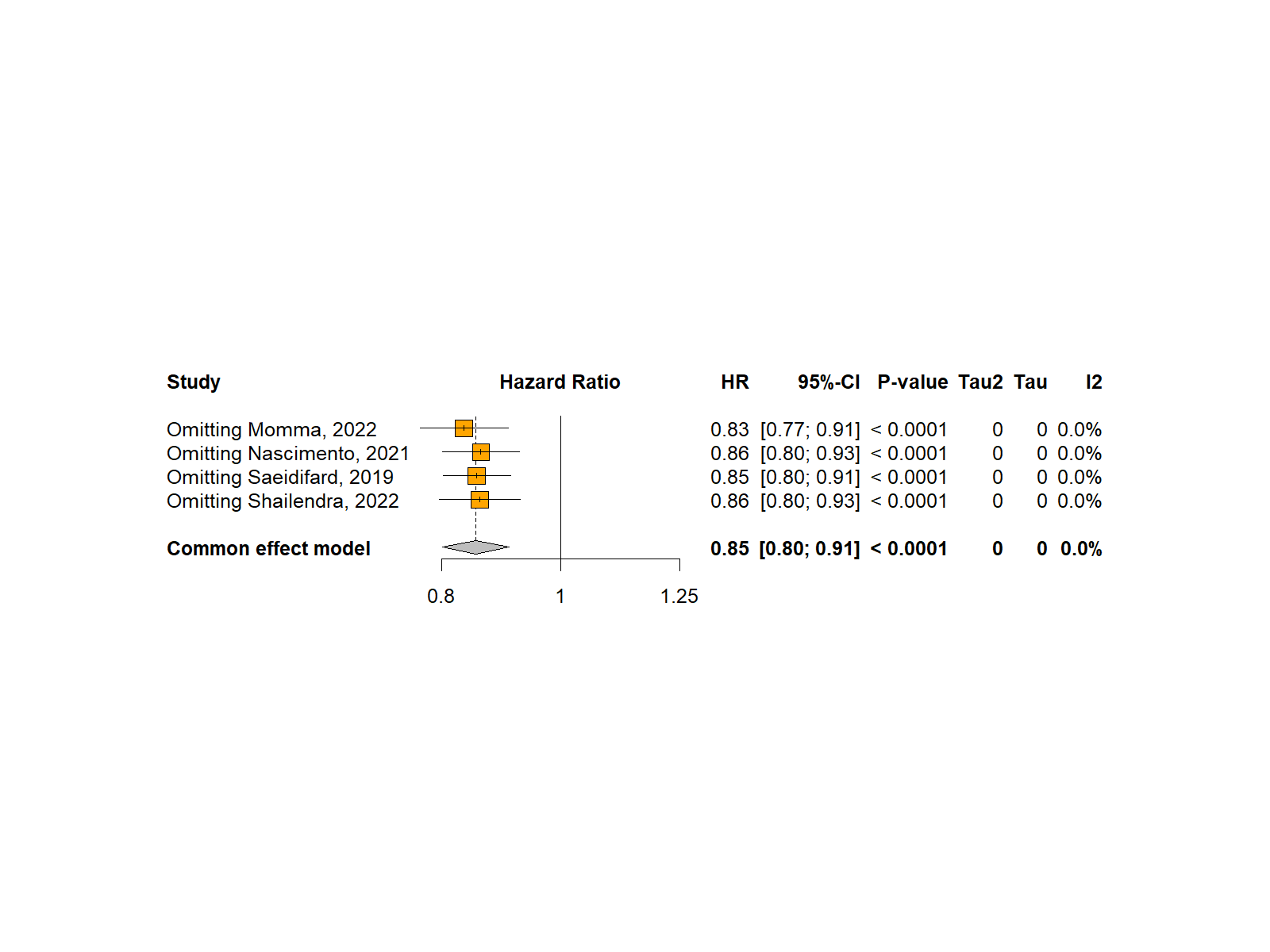


# **Figure 47.** Sensitivity analysis for self-reported TPA and all-cause mortality.

1. Forest plot


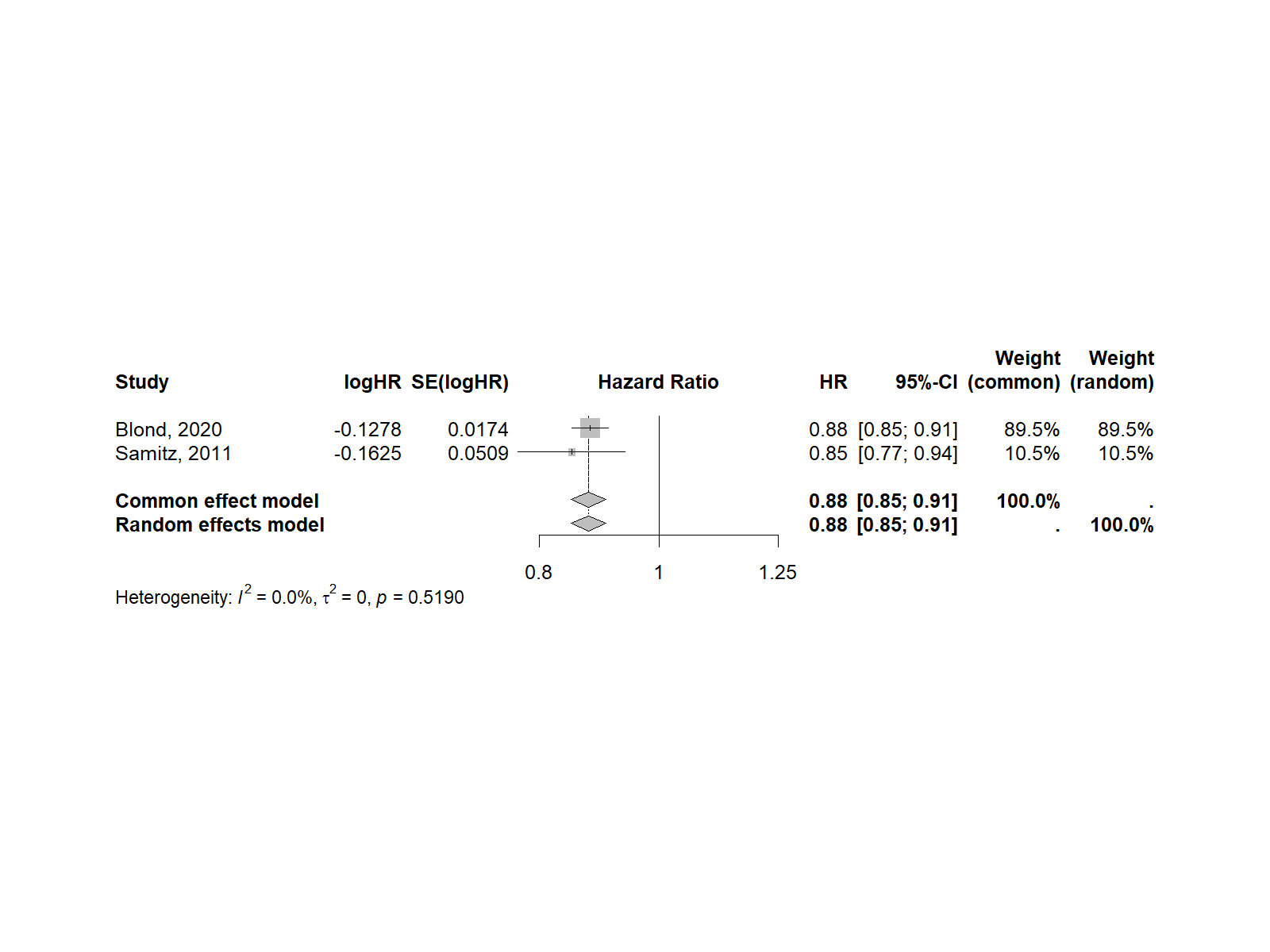


1. Influence diagnostics


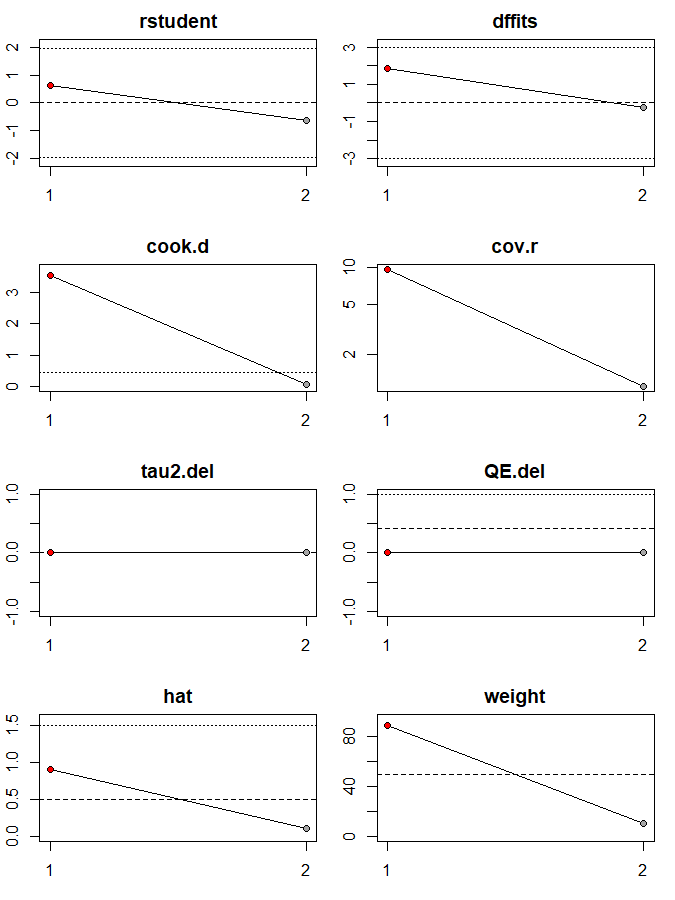


1. Forest plot (sensitivity analysis)


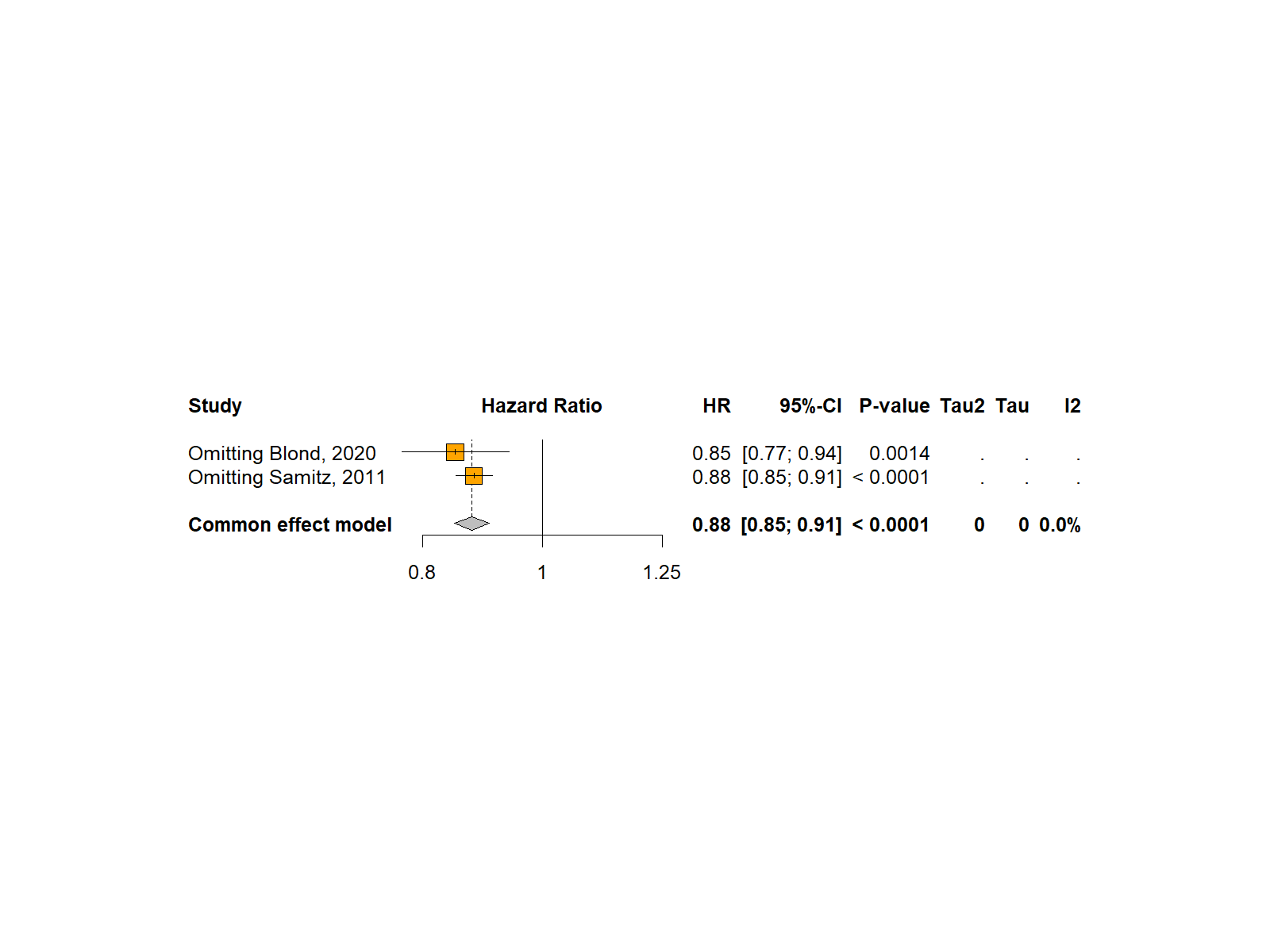


# **Figure 48.** Sensitivity analysis for self-reported SB and CVD mortality.

1. Forest plot


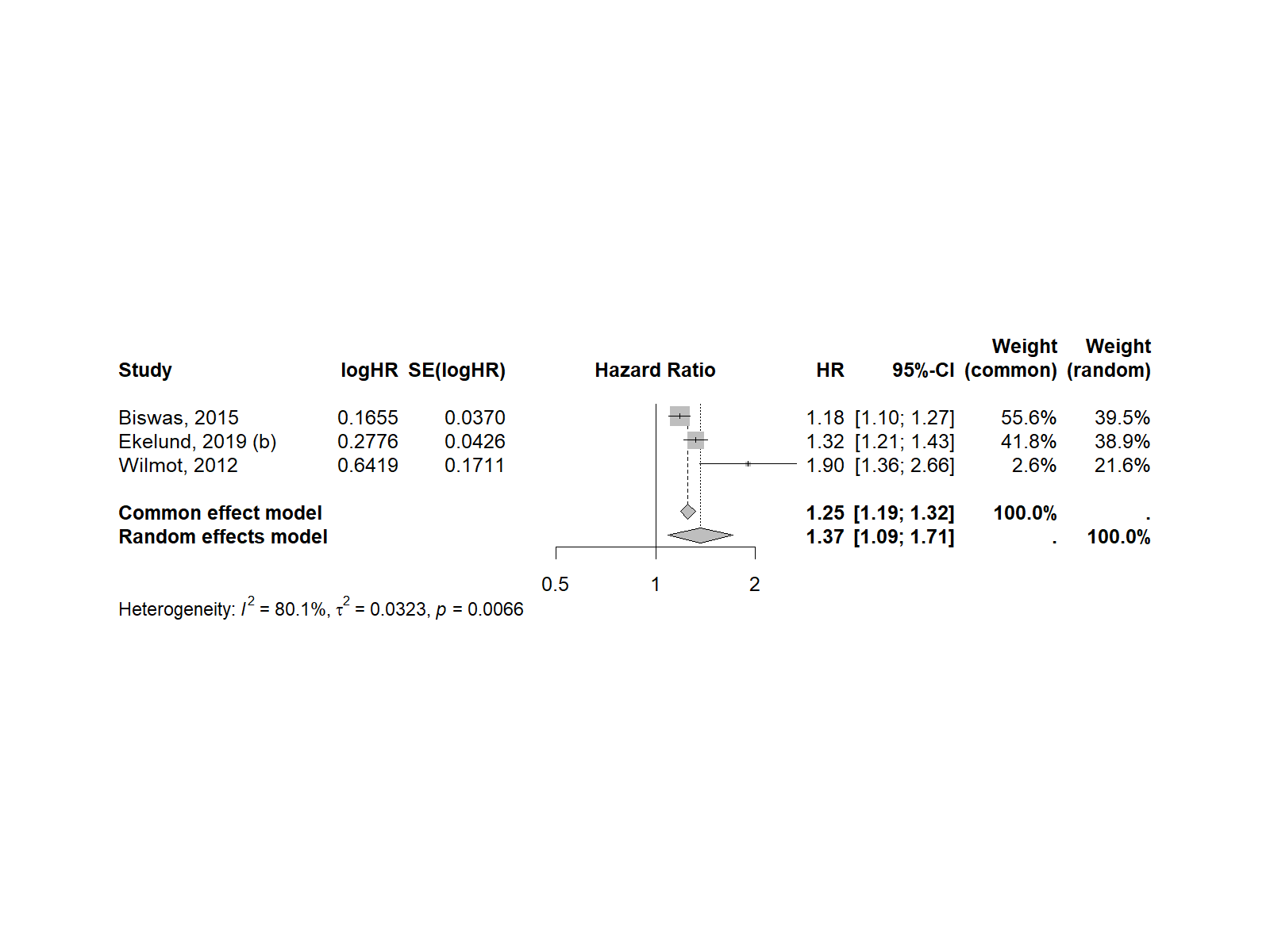


1. Influence diagnostics


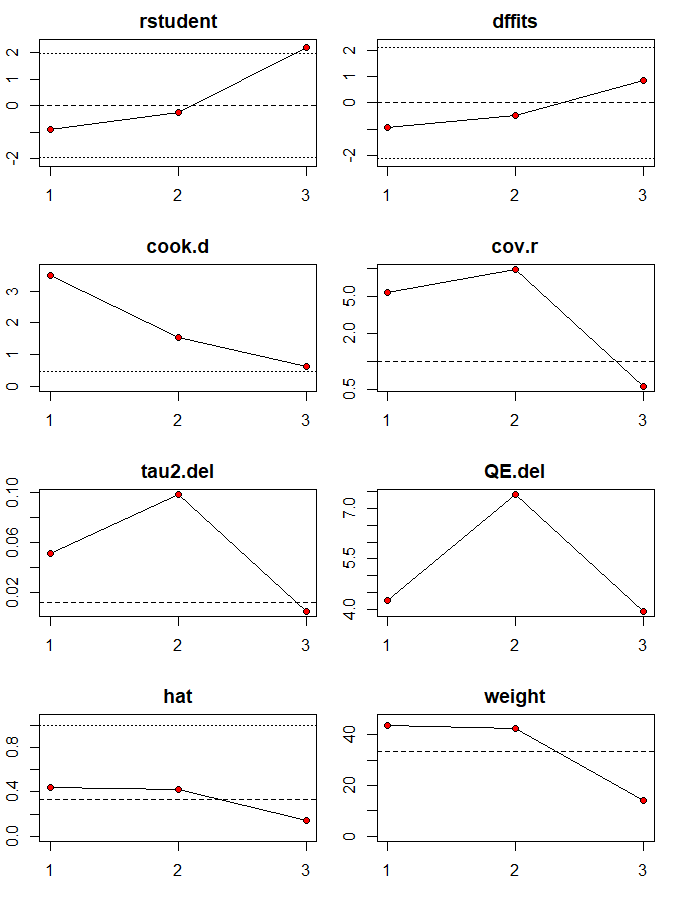


1. Forest plot (sensitivity analysis)


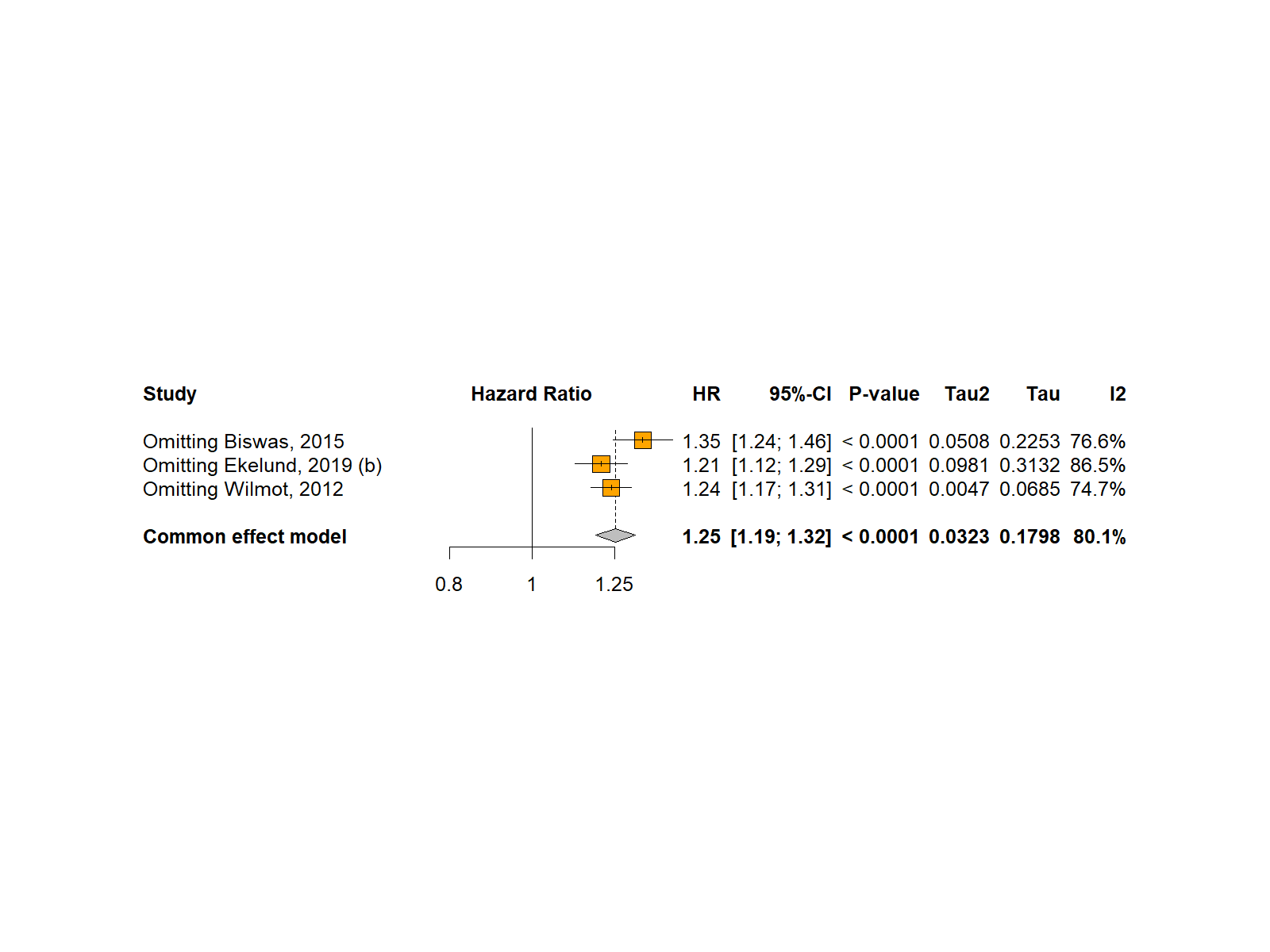


# **Figure 49.** Sensitivity analysis for self-reported SB and cancer mortality.

1. Forest plot


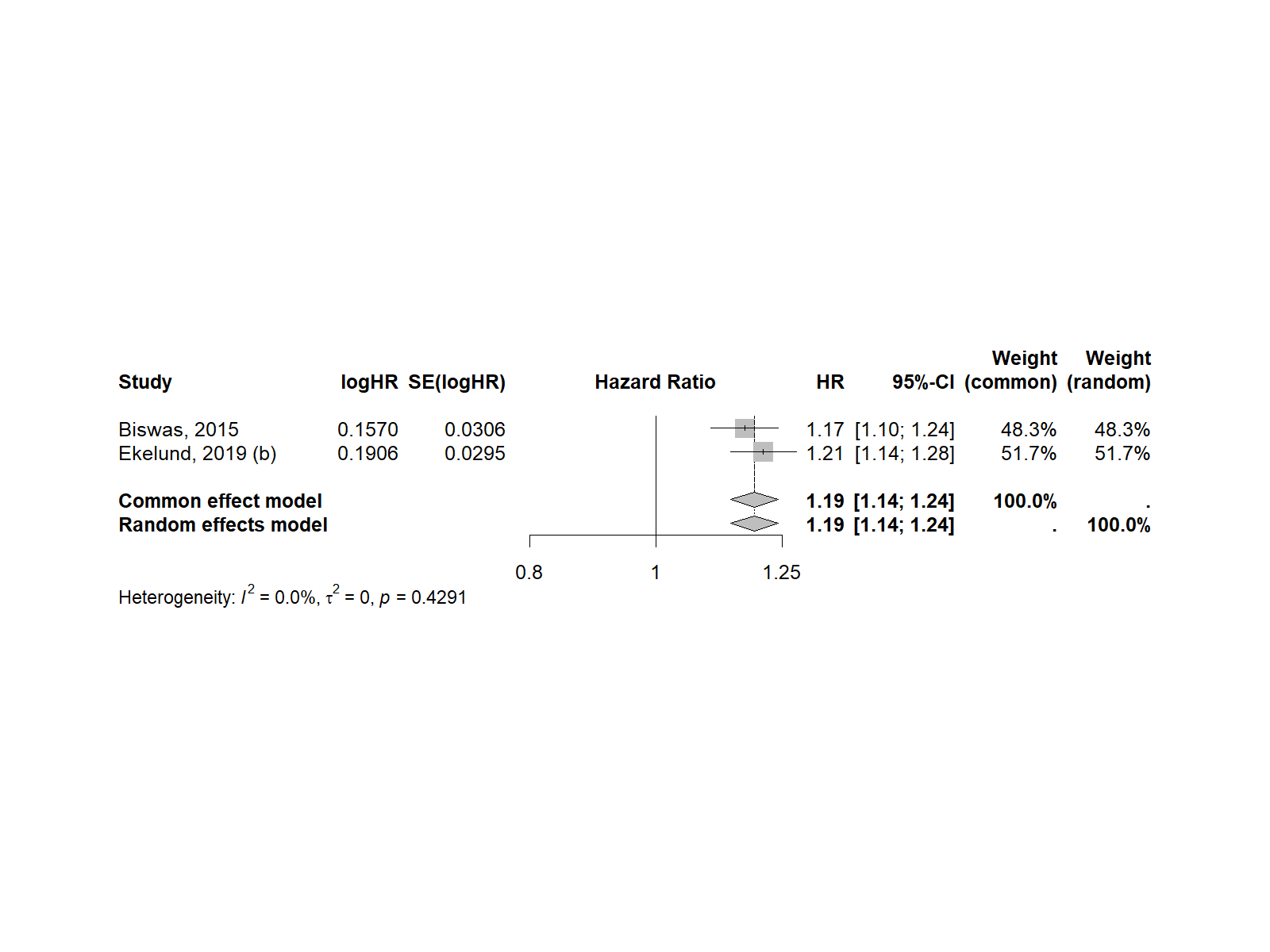


1. Influence diagnostics


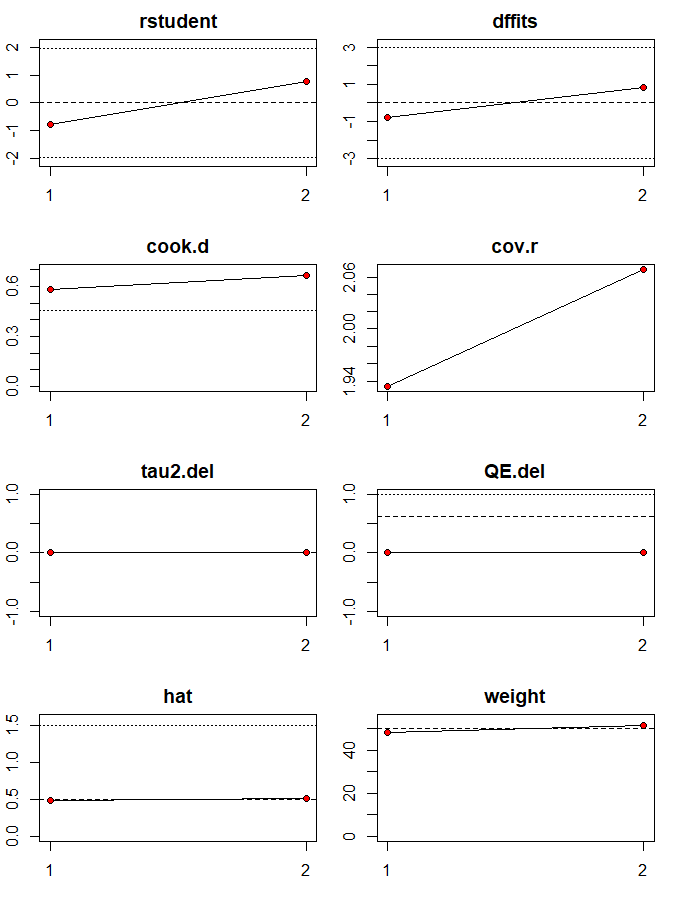


1. Forest plot (sensitivity analysis)


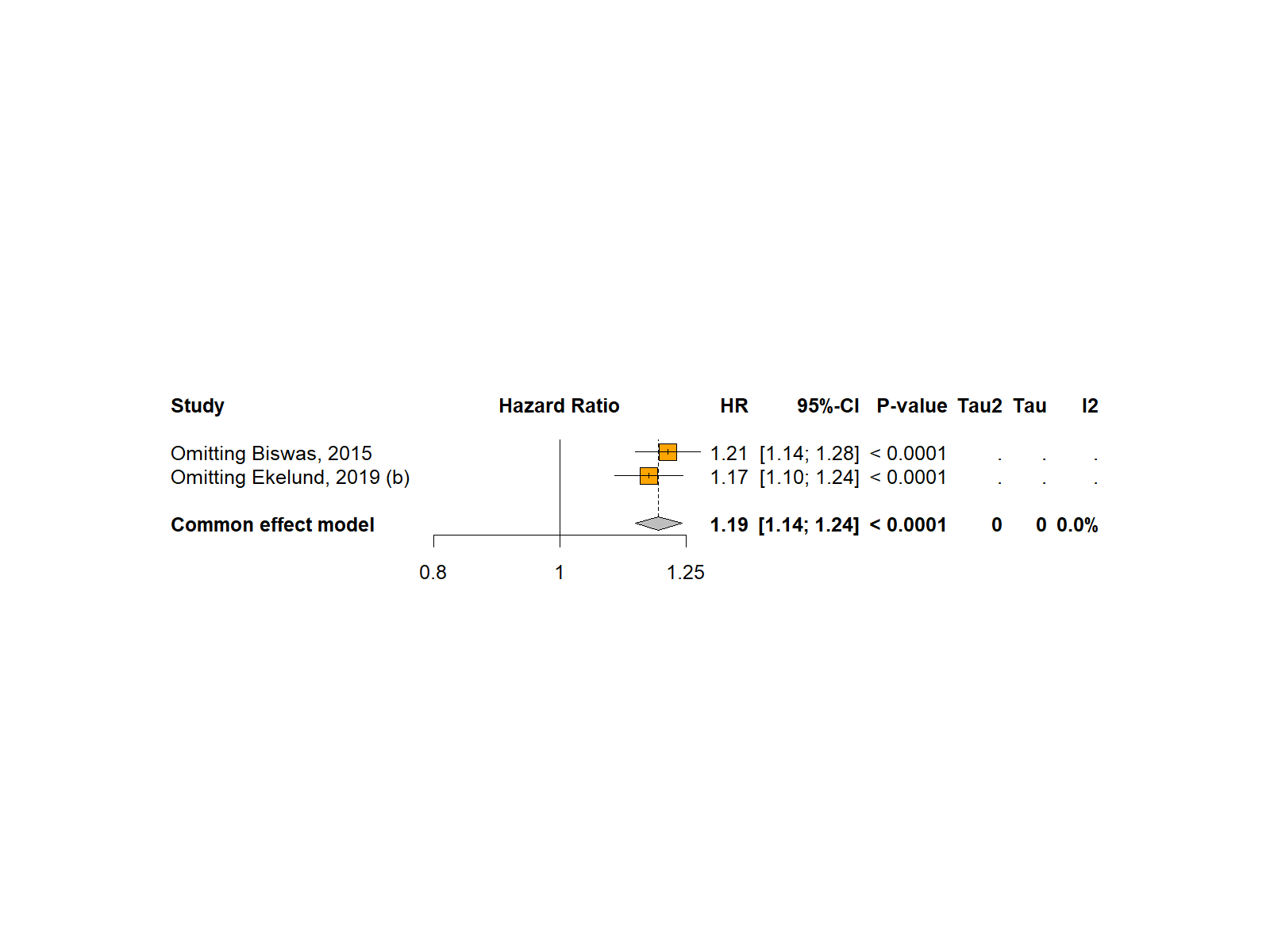


# **Figure 50.** Sensitivity analysis for self-reported walking time and all-cause mortality.

1. Forest plot


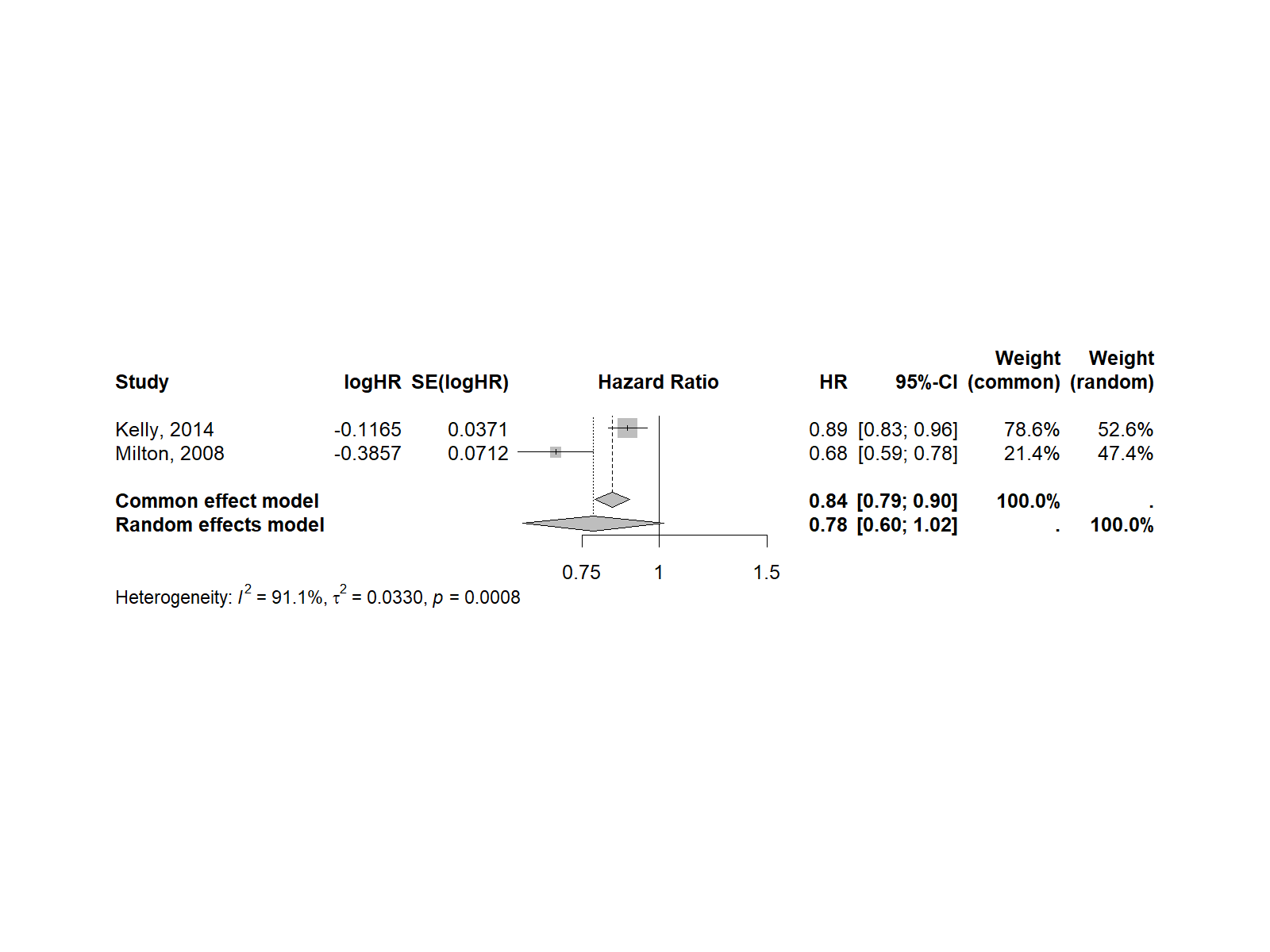


1. Influence diagnostics


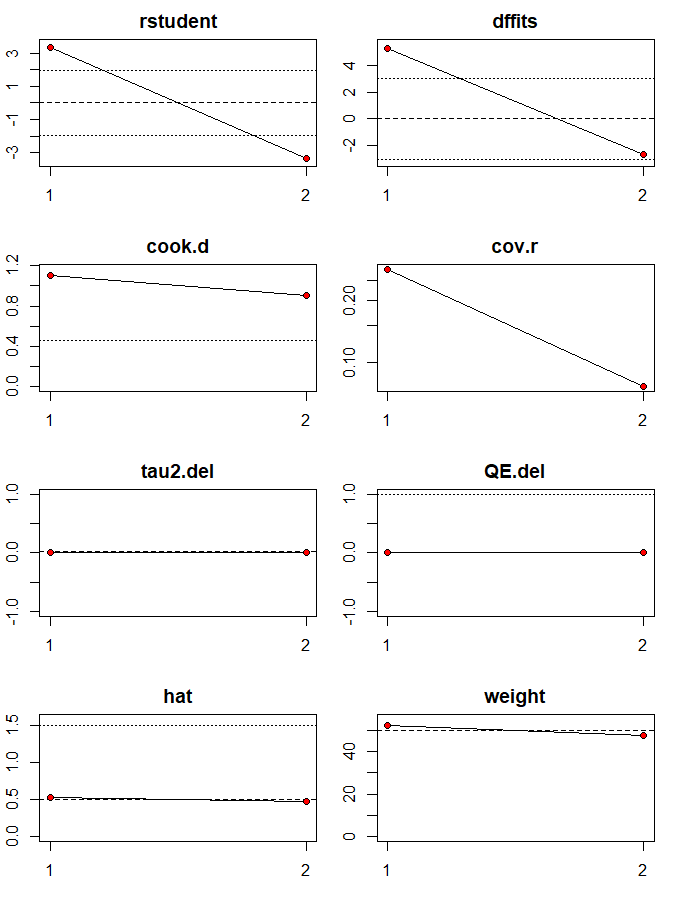


1. Forest plot (sensitivity analysis)


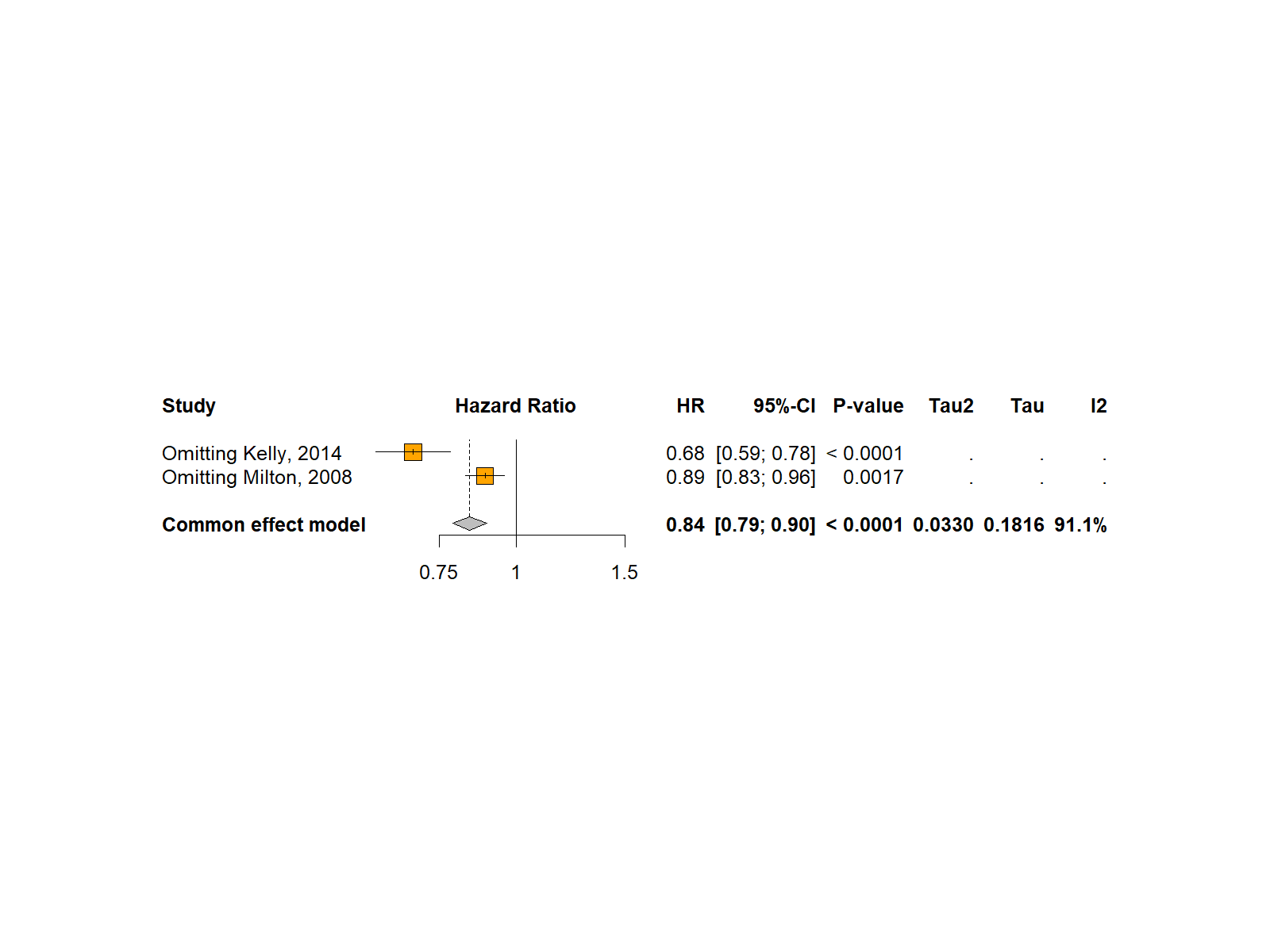


# **Figure 51.** Baujat plot for self-reported LTPA and all-cause mortality.


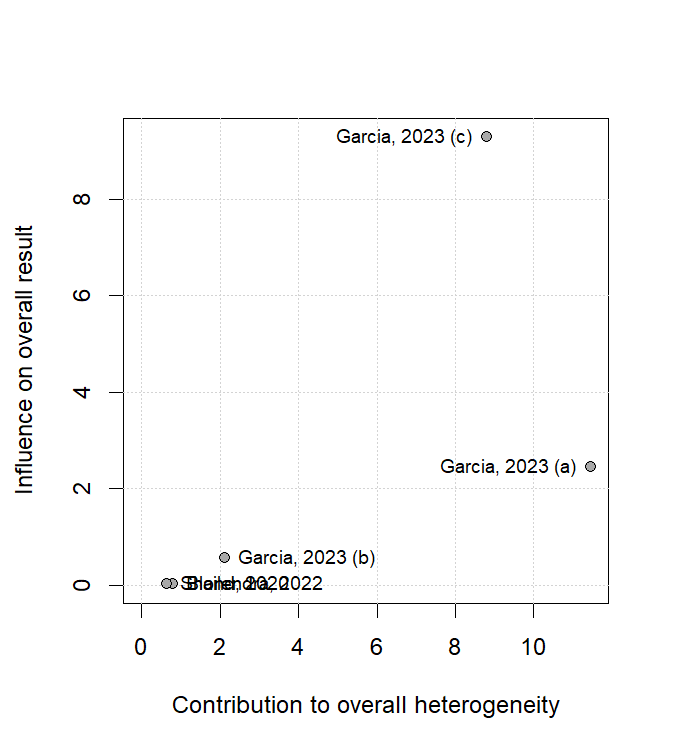


# **Figure 52.** Baujat plot for device-measured TPA and all-cause mortality.

#
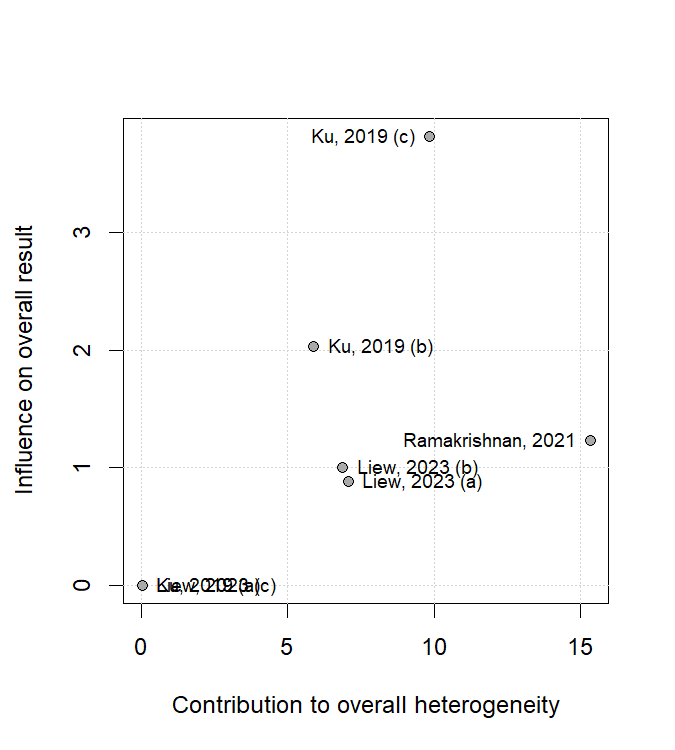


# **Figure 53.** Baujat plot for self-reported SB and all-cause mortality.

# **Figure 54.** Baujat plot for device-measured SB and all-cause mortality.

# **Figure 55.** Baujat plot for PA and all-cause mortality in pooled analysis studies.

# **Figure 56.** Baujat plot for PA and CVD mortality in pooled analysis studies.

# **Figure 57.** Baujat plot for PA and cancer mortality in pooled analysis studies.

# **Figure 58.** Baujat plot for resistance training and all-cause mortality.

# **Figure 59.** Baujat plot for resistance training and cancer mortality.

# **Figure 60.** Baujat plot for self-reported TPA and all-cause mortality.

# **Figure 61.** Baujat plot for self-reported SB and CVD mortality.

# **Figure 62.** Baujat plot for self-reported SB and cancer mortality.

# **Figure 63.** Baujat plot for self-reported walking time and all-cause mortality.
